# Slow Magnetic Relaxation and Luminescent Properties of Mononuclear Lanthanide-Substituted Keggin-Type Polyoxotungstates with Compartmental Organic Ligands

**DOI:** 10.1021/acs.inorgchem.1c03214

**Published:** 2022-01-27

**Authors:** Estibaliz Ruiz-Bilbao, Markel Pardo-Almanza, Itziar Oyarzabal, Beñat Artetxe, Leire San Felices, José A. García, José Manuel Seco, Enrique Colacio, Luis Lezama, Juan M. Gutiérrez-Zorrilla

**Affiliations:** † Departamento de Química Inorgánica, Facultad de Ciencia y Tecnología, Universidad del País Vasco UPV/EHU, P.O. Box 644, 48080 Bilbao, Spain; ‡ Quantum Materials Science Unit, Okinawa Institute of Science and Technology Graduate University, 1919-1 Tancha, Onna, Okinawa 904-0495, Japan; § BCMaterials, Basque Center for Materials, Applications, and Nanostructures, UPV/EHU Science Park, Leioa 48940, Spain; ∥ IKERBASQUE, Basque Foundation for Science, Bilbao 48009, Spain; ⊥ Servicios Generales de Investigación SGIker, Facultad de Ciencia y Tecnología, Universidad del País Vasco UPV/EHU, P.O. Box 644, Bilbao 48080, Spain; # Departamento de Física Aplicada II, Facultad de Ciencia y Tecnología, Universidad del País Vasco UPV/EHU, P.O. Box 644, Bilbao 48080, Spain; ∇ Departamento de Química Aplicada, Facultad de Química, Universidad del País Vasco UPV/EHU, 20018 San Sebastián, Spain; ○ Departamento de Química Inorgánica, Facultad de Ciencias, Universidad de Granada, 18071 Granada, Spain

## Abstract

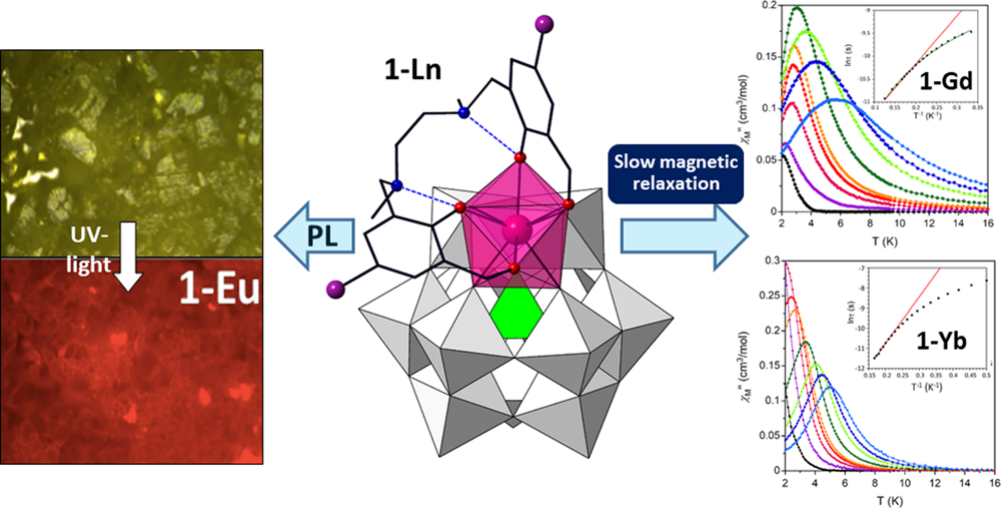

The reaction of mid to late lanthanide ions with the *N*,*N*′-dimethyl-*N*,*N*′-bis(2-hydroxy-3-formyl-5-bromobenzyl)ethylene-diamine organic ligand and monolacunary Keggin type [α-SiW_11_O_39_]^8–^ anion affords a series of isostructural compounds, namely, K_5_[Ln^III^(α-SiW_11_O_39_)(C_20_H_22_Br_2_N_2_O_4_)]·14H_2_O (**1**-Ln, Ln = Sm to Lu). The molecular structure of these sandwich-type complexes is formed by the Ln^III^ ion in a biaugmented trigonal prismatic geometry, which occupies the external O_4_ site of the organic ligand and the vacant site of the lacunary polyoxometalate (POM) unit. The empty N_2_O_2_ coordination site of the organic ligand allows its unprecedented folding, which displays a relative perpendicular arrangement of aromatic groups. Weak Br···Br and π–π interactions established between adjacent molecular units govern the crystal packing, which results in the formation of assemblies containing six hybrid species assembled in a chairlike conformation. **1**-Gd and **1**-Yb display slow relaxation of the magnetization after the application of an external magnetic field with maxima in the out-of-phase magnetic susceptibility plots below ∼5–6 K, which is ascribed to the presence of various relaxation mechanisms. Moreover, photoluminescent emission is sensitized for **1**-Sm and **1**-Eu in the visible region and **1**-Er and **1**-Yb in the NIR. In contrast, the quenching of metal-centered luminescence in the **1**-Tb derivative has been attributed to the out-of-pocket coordination mode of the lanthanide center within the POM fragment. It is demonstrated that the **1**-Yb dual magneto-luminescent material represents the first lanthanide-containing POM reported to date with simultaneous slow magnetic relaxation and NIR emission. Solution stability of the hybrid molecular species in water is also confirmed by ESI-mass spectrometry experiments carried out for **1**-Tb and **1**-Tm.

## Abstract 2

A series of solution stable, molecular polyoxometalate hybrids, namely, [α-SiW_11_O_39_Ln^III^(C_20_H_22_N_2_Br_2_O_4_)]^5−^ (Ln^III^ = Sm to Lu) have been synthesized from the combination of mid-to-late lanthanides, lacunary Keggin-type anions, and a compartmental organic ligand. Metal-centered luminescence in both the visible and NIR region as well as slow relaxation of magnetization was found for different members of this family, including the Yb derivative, which represents the first POM-based system with slow magnetic relaxation and sensitized emission in the NIR region.

## Introduction

Their high solution, thermal, and chemical stability together with remarkable redox properties enables the growing family of polyoxometalates (POMs)^1,2^ to be useful for a wide range of applications in areas like catalysis,^3^ biomedicine,^4^ and material science.^5^ One of the most studied fields within the chemistry of these molecular anionic metal-oxo clusters involves the insertion of electrophiles, either organometallic groups (including organo p-block derivatives)^6^ or metal cations,^7^ into the vacancies of lacunary POM units. This strategy allows the incorporation of additional properties into the system, and therefore, it paves the way for the design of new functional materials.^8^

In the past few years, the combination of lacunary polyoxotungstates that can act as multidentate oxygen donor inorganic ligands with lanthanide (Ln) cations has resulted in some of the most spectacular architectures in terms of size and complexity.^9^ The high oxophilicity and coordination numbers of 4f metal ions allows the formation of giant POMs with more than 100 W centers, as exemplified by the disc-shaped [Ce_16_As_12_(H_2_O)_36_W_148_O_524_]^76−^ anion,^10^ the crown-shaped [K⊂K_7_Ln_24_Ge_12_W_120_O_456_(OH)_12_(H_2_O)_64_]^52–^ (Ln = Ce, Pr, Nd) dodecamers,^11,12^ the elongated [Gd_8_As_12_W_124_O_432_(H_2_O)_22_]^60–^ species,^13^ and the series of elliptic nanoclusters^14^ [Ln_27_Ge_10_W_106_O_406_(OH)_4_(H_2_O)_24_]^59–^ (Ln = La and Ce). In spite of their structural simplicity, small mono- or dimeric species have also attracted enormous interest because of their outstanding properties.^15^ For instance, Ln-containing POMs with accessible coordination sites can behave as effective and recoverable Lewis acid catalysts with enhanced selectivity.^16,17^ Some recent studies take advantage of the Lewis acid character of 4f metals with the Lewis base behavior of oxygen-rich POM surfaces to design efficient bifunctional catalysts.^18^ In addition, their catalytic role in hydrolysis reactions with strong biological implications such as phosphoesterase or protease activity have also been addressed.^19,20^ Nonetheless, similar to that observed for classical coordination complexes, the study of optical and magnetic properties dominates the field of 4f-metal-containing metal-oxo clusters.

With regard to optical properties, parity forbidden 4f–4f transitions of Ln ions can result in bright photoluminescence in the visible to near-infrared (NIR) region if suitable antenna ligands are used. It is well-known that energy transfer from strongly absorbing ligands to emitting centers can populate excited states giving rise to intense and sharp emission bands. Analogous to the behavior of coordination complexes bearing ligands which usually contain aromatic groups,^21^ POMs can sensitize 4f metal ions via O → M (M = Mo, W) ligand-to-metal charge-transfer (LCMT) states.^22^ These multidentate organic moieties or lacunary POM fragments can also block coordination sites to avoid the emission quenching originating from coordinated aqua ligands. Luminescent POMs have been employed as biolabeling agents or sensors as well as incorporated into solid matrixes for the fabrication of switches.^23,24^ It is worth mentioning the [Eu(W_5_O_18_)_2_]^9–^ anion reported by Yamase and Sugeta,^25^ which constitutes the most applied POM in the construction of functional materials.^26,27^

Another field of research with high dynamism within the family of Ln-based compounds is molecular magnetism. The strong magnetic anisotropy together with the large ground-state magnetic moments make 4f ions behave as single-molecule magnets (SMM) under certain crystal-field effects.^28^ These nanomagnets display slow relaxation of the magnetization and quantum effects at low temperatures, and thus, they are suitable candidates for being applied in molecular spintronics, data-storage systems, and quantum computing.^29,30^ The use of low nuclearity 4f metal complexes represents a suitable approach to design systems with slow relaxation of magnetization, in which magnetization reversal is retained by the presence of an energy barrier. In this context, the first mononuclear complex exhibiting SMM behavior was reported in 2003 by Ishikawa’s group.^31^ The double-decker, sandwich-type (Bu_4_N)[LnPc_2_] (Ln = Tb, Dy; Pc = phthalocyanine) complexes are formed by two rigid and multidentate phthalocyanine ligands which enclose a central 4f metal with square antiprismatic geometry (*D*_4*d*_). Among the tens if not hundreds of examples reported since then,^32^ some of us prepared a series of SMMs with aminophenol Mannich base derivatives as ligands.^33^ Reaction between secondary amines, paraformaldehyde, and phenol moieties can easily afford multidentate O- and N-donor ligands with two different potential coordination sites. For example, the *N*,*N*′-dimethyl-*N*,*N*′-bis(2-hydroxy-3-formyl-5-bromobenzyl)ethylene-diamine (H_2_L) displays an outer O_4_ site which can easily accommodate large oxophilic 4f metals and the inner N_2_O_2_ pocket, which is available for the coordination of smaller 3d metal centers. In some cases, heterometallic 3d–4f complexes^34−36^ have been identified as a convenient way to improve the SMM properties of a given complex, because (i) the use of diamagnetic 3d metal ions can attenuate the intermolecular magnetic interactions responsible for quantum tunneling of magnetization (QTM) and, consequently, the loss of magnetization and (ii) strong magnetic exchange interactions can fully or partially quench QTM.

When it comes to Ln-substituted POMs with SMM behavior,^37^ they exhibit some advantages in comparison to classical coordination complexes: (i) lacunary POM ligands show higher thermal and chemical stability both in solution and in the solid state; (ii) the rigidity of the ligand can result in highly symmetric environments for the 4f centers, or even force unusual geometries such as the 5-fold *C*_5_ symmetry;^38^ (iii) their large size and diamagnetism ensures magnetic isolation over the neighboring species. In this regard, different series of Peacock–Weakley-type assemblies in which Ln centers with square antiprismatic geometry are trapped between lacunary fragments, i.e., [Ln(W_5_O_18_)_2_]^9–^ (Ln^III^ = Tb, Dy, Ho, and Er), [Er(β_2_-GeW_11_O_39_)(α-GeW_11_O_39_)]^13–^, and [Ln(β_2_-SiW_11_O_39_)_2_]^13–^ (Ln^III^ = Dy, Ho, Er, and Yb), have displayed slow relaxation of the magnetization.^39−41^ The high coherence of the [Ho(W_5_O_18_)_2_]^9–^ molecular qubit should be mentioned here.^42^ The chemically controlled reversible switching of the SMM behavior has also been achieved for POM-based systems.^43^

Despite this potential, the simultaneous coordination of both lacunary POMs and multidentate aromatic ligands to 4f metal centers in quest of optical and magnetic properties has been comparatively less examined. In fact, very recent studies on compounds [*n*-NBu_4_]_3_[LnH(PW_11_O_39_)(phen)_2_]·H_2_O (Ln = Dy, Er, phen = phenantroline) represent the first examples in the literature of mononuclear hybrid organic–inorganic complexes with SMM behavior.^44^ The incorporation of organic ligands to the system has proven to be a suitable approach to dramatically lower magnetic relaxation times in comparison to the purely inorganic analogues. In the particular case of compartmental ligands, coordination of the [CuTbL_Schiff_(H_2_O)_3_Cl_2_]Cl complex (L_Schiff_ = N,N′-bis(3-methoxysalicylidene)ethylenediamine) to the oxygen-rich surface of a POM anion induced SMM behavior in a precursor that did not exhibit slow relaxation of the magnetization by itself.^45^ Moreover, efficient sensitization of 4f-metal-containing POMs has also been achieved by the simultaneous coordination of aromatic antenna ligands (e.g., picolinate, benzoate, phenantroline) to the emitting centers.^46−48^ Considering all of the above, herein we report on the synthesis, structure, and solution stability of a series of 10 hybrid anions formed by mid-to-late lanthanide-containing Keggin-type polyoxotungstates and the compartmental organic ligand H_2_L, namely, K_5_[Ln(α-SiW_11_O_39_)(H_2_L)]·14H_2_O (**1**-Ln, Ln = Sm to Lu). It is worth noting that they represent the first examples in the literature of mononuclear lanthanide complexes with this specific organic ligand. A complete solid state photophysical analysis has shown the efficient emission of different **1**-Ln derivatives in the visible and NIR regions, whereas magnetic studies have revealed slow relaxation of magnetization for **1**-Gd and **1**-Yb analogues under the presence of an external field. It is demonstrated that the **1**-Yb derivative can be regarded as the first POM-based system with simultaneous slow magnetic relaxation and NIR emission. It is worth noting that some luminescent SMMs reported in the literature^49^ have displayed emission switching under magnetic fields,^50^ and more specifically, Yb derivatives can also show great potential as luminescent thermometers,^51−53^ which confirms the high level of interest in this research field.

## Experimental Section

### Materials and Methods

The monolacunary Keggin-type K_8_[α-SiW_11_O_39_]·13H_2_O precursor^54^ and the *N*,*N*′-dimethyl-*N*,*N*′-bis(2-hydroxy-3-formyl-5-bromobenzyl)ethylene-diamine (H_2_L) ligand (Figure S1)^55^ were synthesized following reported procedures and identified by FT-IR and ^1^H NMR, respectively. All other chemicals were purchased from commercial sources and used without further purification. Carbon, hydrogen, and nitrogen (CHN) contents were determined on a PerkinElmer 2400 CHN analyzer. Metal analyses were performed using a Q-ICP-MS ThermoXSeries II analyzer. Fourier transformed infrared (FT-IR) spectra were obtained as KBr pellets on a Shimadzu FTIR-8400S spectrometer. PXRD patterns were recorded from 2θ = 5 to 50° (0.03° step size, 30 s per step) using a Philips X’PERT PRO diffractometer operating at 40 kV/40 mA in θ–θ configuration with monochromated Cu Kα radiation (λ = 1.5418 Å) and a PIXcel detector. Magnetic susceptibilities were measured in the 2–300 K range using a Quantum Design MPMS3 SQUID magnetometer under an applied field of 0.1 T (diamagnetic corrections were estimated from Pascal’s constants). Magnetization and alternating current (ac) susceptibility measurements were performed on a PPMS (Physical Property measurement System)–Quantum Design Model 6000 and the SQUID magnetometers in the 2–10 K temperature range up to a 7 T magnetic field.

Diffuse reflectance UV–vis spectra were recorded on a UV-2600 Shimadzu spectrophotometer. Photoluminescence (PL) emission spectra were recorded for powdered samples from 10 K to room temperature using a close cycle helium cryostat contained in an Edinburgh Instruments FLS920 spectrometer equipped with a Müller-elektronik-Optik SVX1450 Xe lamp and a Kimmon IK3552R-G He:Cd continuous laser (325 nm). The lifetime measurements were performed using a μF1 pulsed microsecond flashlamp as an excitation source. Photographs were taken in a micro-PL system included in an Olympus optical microscope (Color View III camera) illuminated with a Hg lamp.

Electrospray ionization mass spectra (ESI-MS) were obtained on aqueous solutions of solid samples that were diluted to 10^–5^ M approximately with a mixture of H_2_O/CH_3_CN (1:1) and introduced at a flow rate of 10 μL min^–1^ using Waters SYNAPT G2 HDMS QTOF instrument with an orthogonal Z-spray electrospray interface operating with capillary voltage of 3.3 kV in the negative scan mode (V mode) and N_2_ as desolvation (300 L h^–1^) and cone gas (30 L h^–1^). Typical desolvation (200 °C) and source block (120 °C) temperatures were used, and the cone voltage (*U*_c_) was set to 15 V.

### General Synthetic Procedure

A mixture of the H_2_L ligand (0.051 g, 0.1 mmol) and the corresponding lanthanide salt (0.1 mmol) in 5 mL of MeOH was added dropwise to a solution of K_8_[α-SiW_11_O_39_]·13H_2_O precursor (0.322 g, 0.1 mmol) in 25 mL of 0.5 M aqueous KAc/HAc buffer (pH = 4.6) at 90 °C. The resulting solution was heated for 30 min, filtered, and left to evaporate at room temperature in an open container. Powders generated over 12 h were filtered off, and yellow single crystals of K_5_[Ln^III^(α-SiW_11_O_39_)(H_2_L)]·14H_2_O (Ln^III^ = Sm to Lu, **1**-Ln; H_2_L = C_20_H_22_Br_2_N_2_O_4_) were obtained from the resulting clear solutions in less than 1 week.

#### K_5_[Sm(α-SiW_11_O_39_)(C_20_H_22_Br_2_N_2_O_4_)]·14H_2_O (**1**-Sm)

Sm(NO_3_)_3_·6H_2_O (0.044 g) was used as the 4f metal source. Yield: 19 mg, 5% based on W. IR: ν 1625 (vs), 1544 (s), 1443 (m), 1419 (w), 1380 (w), 1204 (w), 1162 (w), 1001 (m), 941 (s), 885 (vs), 766 (w), 748 (w) 790 (m), 701 (m), 507 (w). Elem Anal. calcd (%) for C_20_H_50_Br_2_K_5_N_2_O_57_SiSmW_11_: C, 6.34%; H, 1.33%; K, 5.53%; N, 0.74%; Si, 0.80%; Sm, 4.25%. Found: C, 6.03%; H, 1.68%; K, 5.57%; N, 0.79%; Si, 0.77%; Sm, 4.20%.

#### K_5_[Eu(α-SiW_11_O_39_)(C_20_H_22_Br_2_N_2_O_4_)]·14H_2_O (**1**-Eu)

EuCl_3_·6H_2_O (0.026 g) was used as the 4f metal source. Yield: 16 mg, 4% based on W. IR: ν 1624 (vs), 1540 (s), 1441 (m), 1422 (w), 1381 (w), 1207 (w), 1163 (w), 1003 (m), 940 (s), 885 (vs), 767 (w), 748 (w) 792 (m), 702 (m), 506 (w). Elem Anal. calcd (%) for C_20_H_50_Br_2_EuK_5_N_2_O_57_SiW_11_: C, 6.34%; H, 1.33%; Eu, 4.30%; K, 5.53%; N, 0.74%; Si, 0.79%. Found: C, 6.11%; H, 1.57%; Eu, 4.17%; K, 5.62%; N, 0.62%; Si, 0.75%.

#### K_5_[Gd(α-SiW_11_O_39_)(C_20_H_22_Br_2_N_2_O_4_)]·14H_2_O (**1**-Gd)

Gd(NO_3_)_3_·6H_2_O (0.045 g) was used as the 4f metal source. Yield: 21 mg, 6% based on W. IR: ν 1627 (vs), 1545 (s), 1444 (m), 1423 (w), 1384 (w), 1209 (w), 1165 (w), 1007 (m), 940 (s), 884 (vs), 769 (w), 746 (w), 795 (m), 702 (m), 505 (w). Elem Anal. calcd (%) for C_20_H_50_Br_2_GdK_5_N_2_O_57_SiW_1_1: C, 6.33%; H, 1.33%; Gd, 4.44%; K, 5.52%; N, 0.74%; Si, 0.79%. Found: C, 6.18%; H, 1.71%; Gd, 4.36%; K, 5.48, N, 0.79%; Si, 0.76%.

#### K_5_[Tb(α-SiW_11_O_39_)(C_20_H_22_Br_2_N_2_O_4_)]·14H_2_O (**1**-Tb)

Tb(NO_3_)_3_·5H_2_O (0.044 g) was used as the 4f metal source. Yield: 17 mg, 4% based on W. IR: ν 1622 (vs), 1541 (s), 1437 (m), 1421 (w), 1383 (w), 1206 (w), 1159 (w), 1002 (m), 944 (s), 880 (vs), 768 (w), 749 (w), 789 (m), 702 (m), 503 (w). Elem Anal. calcd (%) for C_20_H_50_Br_2_K_5_N_2_O_57_SiTbW_11_: C, 6.33%; H, 1.33%; K, 5.52%; N, 0.74%; Si, 0.79%; Tb, 4.49%. Found: C, 6.20%; H, 1.64%; K, 5.55%; N, 0.81%; Si, 0.80%; Tb, 4.43%.

#### K_5_[Dy(α-SiW_11_O_39_)(C_20_H_22_Br_2_N_2_O_4_)]·14H_2_O (**1**-Dy)

Dy(NO_3_)_3_·6H_2_O (0.045 g) was used as the 4f metal source. Yield: 21 mg, 6% based on W. IR: ν 1621 (vs), 1546 (s), 1441 (m), 1420 (w), 1378 (w), 1201 (w), 1162 (w), 1003 (m), 940 (s), 883 (vs), 766 (w), 747 (w), 792 (m), 702 (m), 501 (w). Elem Anal. calcd (%) for C_20_H_50_Br_2_GdK_5_N_2_O_57_S iW_11_: C, 6.32%; H, 1.33%; Gd, 4.44%; K, 5.52%; N, 0.74%; Si, 0.79%. Found: C, 6.12%; H, 1.62%; Gd, 4.36%; K, 5.48, N, 0.77%; Si, 0.76%.

#### K_5_[Ho(α-SiW_11_O_39_)(C_20_H_22_Br_2_N_2_O_4_)]·14H_2_O (**1**-Ho)

Ho(NO_3_)_3_·5H_2_O (0.044 g) was used as the 4f metal source. Yield: 22 mg, 6% based on W. IR: ν 1623 (vs), 1547 (s), 1444 (m), 1419 (w), 1381 (w), 1208 (w), 1162 (w), 1001 (m), 940 (s), 885 (vs), 768 (w), 748 (w), 790 (m), 70 2 (m), 505 (w). Elem Anal. calcd (%) for C_20_H_50_Br_2_HoK_5_N_2_O_57_SiW_11_: C, 6.32%; H, 1.33%; Ho, 4.65%; K, 5.51%; N, 0.74%; Si, 0.79%. Found: C, 6.18%; H, 1.67%; Ho, 4.71%; K, 5.69%; N, 0.74%; Si, 0.78%.

#### K_5_[Er(α-SiW_11_O_39_)(C_20_H_22_Br_2_N_2_O_4_)]·14H_2_O (**1**-Er)

Er(NO_3_)_3_·5H_2_O (0.044 g) was used as the 4f metal source. Yield: 14 mg, 4% based on W. IR: ν 1623 (vs), 1544 (s), 1443 (m), 1421 (w), 1381 (w), 1207 (w), 1162 (w), 1001 (m), 940 (s), 885 (vs), 769 (w), 747 (w), 790 (m), 702 (m), 505 (w). Elem Anal. calcd (%) for C_20_H_50_Br_2_ErK_5_N_2_O_57_SiW_11_: C, 6.32%; H, 1.32%; Er, 4.71%; K, 5.51%; N, 0.74%; Si, 0.79%. Found: C, 6.38%; H, 1.89%; Er, 4.60%; K, 5.59%; N, 0.84%; Si, 0.72%.

#### K_5_[Tm(α-SiW_11_O_39_)(C_20_H_22_Br_2_N_2_O_4_)]·14H_2_O (**1**-Tm)

Tm(NO_3_)_3_·5H_2_O (0.045 g) was used as the 4f metal source. Yield: 19 mg, 5% based on W. IR: ν 1623 (vs), 1544 (s), 1444 (m), 1419 (w), 1381 (w), 1207 (w), 1162 (w), 1001 (m), 942 (s), 885 (vs), 767 (w), 750 (w),790 (m), 702 (m), 505 (w). Elem Anal. calcd (%) for C_20_H_50_Br_2_K_5_N_2_O_57_SiTmW_11_: C, 6.31%; H, 1.32%; K, 5.50%; N, 0.74%; Si, 0.79%; Tm, 4.75%. Found: C, 6.22%; H, 1.34%; K, 5.53%; N, 0.68%; Si, 0.80%; Tm, 4.72%.

#### K_5_[Yb(α-SiW_11_O_39_)(C_20_H_22_Br_2_N_2_O_4_)]·14H_2_O (**1**-Yb)

Yb(NO_3_)_3_·5H_2_O (0.045 g) was used as the 4f metal source. Yield: 18 mg, 5% based on W. IR: ν 1623 (vs), 1547 (s), 1444 (m), 1420 (w), 1381 (w), 1207 (w), 1162 (w), 1003 (m), 940 (s), 885 (vs), 766 (w), 747 (w), 790 (m), 702 (m), 505 (w). Elem Anal. calcd (%) for C_20_H_50_Br_2_K_5_N_2_O_57_SiW_11_Yb: C, 6.31%; H, 1.32%; K, 5.50%; N, 0.74%; Si, 0.79%; Yb, 4.86%. Found: C, 6.28%; H, 1.52%; K, 5.61%; N, 0.67%; Si, 0.83%; Yb, 4.94%.

#### K_5_[Lu(α-SiW_11_O_39_)(C_20_H_22_Br_2_N_2_O_4_)]·14H_2_O (**1**-Lu)

Lu(NO_3_)_3_·5H_2_O (0.046 g) was used as the 4f metal source. Yield: 20 mg, 5% based on W. IR: ν 1623 (vs), 1546 (s), 1444 (m), 1419 (w), 1381 (w), 1205 (w), 1162 (w), 1001 (m), 940 (s), 885 (vs), 765 (w), 748 (w), 790 (m), 702 (m), 505 (w). Elem Anal. calcd (%) for C_20_H_50_Br_2_K_5_LuN_2_O_57_SiW_11_: C, 6.30%; H, 1.32%; K, 5.49%; Lu, 4.92%; N, 0.74%; Si, 0.79%. Found: C, 6.34%; H, 1.47%; K, 5.59%; Lu, 4.89%; N, 0.75%; Si, 0.77%.

### Thermal Analyses

Thermogravimetric analyses (TGA) were performed on a Mettler Toledo TGA/SDTA851^e^ thermobalance under a 50 cm^3^ min^–1^ flow of synthetic air from room temperature to 800 °C at a rate of 5 °C min^–1^. Solid crystalline samples were filtered from mother solutions and left to dry overnight before their analysis. All compounds **1**-Ln show similar TGA curves (Figure S2). The dehydration process is observed as a continuous mass loss, which extends from room temperature up to 120 °C and accounts for 5.9% (**1**-Sm and **1**-Ho) to 7.2% (**1**-Lu) of the initial mass. Thus, a total number of 14 hydration molecules was established per molecular formula of **1**-Ln on the basis of these calculations. The combustion of the organic ligand and breakdown of the POM architecture take place in a second thermal event, which lead to the final residues at temperatures in the 650–750 °C range. Two major phases have been identified in these residues by X-ray diffraction, which correspond to hexagonal K_0.33_WO_3.16_ (PDF: 00–020–0940) and analogues of the monoclinic *C*2/*c* KLn(WO_4_)_2_ (Ln = Sm to Lu; PDF for Ln = Dy: 00–023–0479) phase (Figure S3). Table S1 in the SI compiles decomposition temperatures (*T*_d_) together with the experimental and calculated mass losses (Δ*m*) for the dehydration step and final residues.

### Single-Crystal X-ray Crystallography

Crystallographic data for compounds **1**-Ln (Ln = Sm to Lu) are summarized in Table 1. Intensity data were collected at 100(2) K on an Agilent Technologies SuperNova diffractometer equipped with monochromated Cu Kα radiation (λ = 1.5406 Å) and an Atlas detector for **1**-Eu to **1**-Tb and Mo Kα radiation (λ = 0.71073 Å) and an Eos CCD detector for the rest of the samples. Data frames were processed (unit cell determination, analytical absorption correction with face indexing, intensity data integration, and correction for Lorentz and polarization effects) with the CrysAlis Pro software package.^56^ The structures were solved using the OLEX2^57^ program and refined by full-matrix least-squares using SHELXL-2014/6 and SHELXH-97.^58,59^ Final geometrical calculations were carried out with PLATON^60^ as integrated in WinGX,^61^ and their visualization was performed using CrystalMaker,^62^ whereas SHAPE^63^ was employed to perform continuous shape measurements.

**Table 1:** Crystallographic Data for **1**-Ln (Ln = Sm–Lu)

	**1**-Sm	**1**-Eu	**1**-Gd	**1**-Tb	**1**-Dy
empirical formula	C_20_H_50_Br_2_K_5_N_2_O_57_SiSmW_11_	C_20_H_50_Br_2_EuK_5_N_2_O_57_SiW_11_	C_20_H_50_Br_2_GdK_5_N_2_O_57_SiW_11_	C_20_H_50_Br_2_K_5_N_2_O_57_SiTbW_11_	C_20_H_50_Br_2_DyK_5_N_2_O_57_SiW_11_
fw (g mol^–1^)	3786.73	3788.34	3793.63	3795.30	3798.88
cryst syst	triclinic	triclinic	triclinic	triclinic	triclinic
space group (number)	*P*1̅ (2)	*P*1̅ (2)	*P*1̅ (2)	*P*1̅ (2)	*P*1̅ (2)
*a* (Å)	21.8698(5)	21.7085(3)	21.9076(3)	21.8324(2)	21.8829(3)
*b* (Å)	24.7133(7)	24.6560(4)	24.7469(5)	24.6742(4)	24.7289(7)
*c* (Å)	28.4487(5)	28.2290(4)	28.4996(5)	28.2891(5)	28.3578(6)
α (deg)	113.136(2)	113.8651(14)	113.1773(17)	112.7352(15)	112.833(2)
β (deg)	95.4719(18)	95.3806(12)	95.4645(13)	95.2612(11)	95.4831(15)
γ (deg)	103.874(2)	103.7301(13)	103.8326(14)	104.1432(11)	104.0587(19)
*V* (Å^3^)	13413.7(5)	13112.8(3)	13478.1(4)	13330.7(3)	13406.7(5)
ρ_calcd_ (g cm^–3^)	2.813	2.878	2.804	2.837	2.823
μ (mm^–1^)	15.956	35.113	33.938	33.400	16.143
reflns collected	103153	98099	103890	98895	92085
unique reflns (*R*_int_)	48790 (0.099)	49575 (0.090)	50920 (0.077)	50232 (0.057)	51216 (0.052)
observed reflns [*I* > 2σ(*I*)]	27923	37493	39632	39221	33784
params (restraints)	1503 (0)	1444 (0)	1478 (6)	1538 (6)	1508 (6)
*R*(*F*)^a^ [*I* > 2σ(*I*)]	0.091	0.100	0.081	0.060	0.074
*wR*(*F*^2^)^b^ [all data]	0.274	0.279	0.226	0.171	0.191
GoF	1.047	1.037	1.051	1.034	1.057

	**1**-Ho	**1**-Er	**1**-Tm	**1**-Yb	**1**-Lu
empirical formula	C_20_H_50_Br_2_HoK_5_N_2_O_57_SiW_11_	C_20_H_50_Br_2_ErK_5_N_2_O_57_SiW_11_	C_20_H_50_Br_2_K_5_N_2_O_57_SiTmW_11_	C_20_H_50_Br_2_K_5_N_2_O_57_SiW_11_Yb	C_20_H_50_Br_2_K_5_LuN_2_O_57_SiW_11_
fw (g mol^–1^)	3801.31	3803.64	3805.31	3809.42	3811.35
cryst syst	triclinic	triclinic	triclinic	triclinic	triclinic
space group (number)	*P*1̅ (2)	*P*1̅ (2)	*P*1̅ (2)	*P*1̅ (2)	*P*1̅ (2)
*a* (Å)	21.8093(5)	21.8169(4)	21.6072(5)	21.8183(5)	21.7850(6)
*b* (Å)	24.6894(4)	24.6880(6)	24.5873(7)	24.6679(5)	24.6164(7)
*c* (Å)	28.3007(5)	28.3880(8)	28.1327(6)	28.2720(8)	28.1935(7)
α (deg)	113.5373(18)	113.204(3)	113.784(3)	113.330(2)	113.292(3)
β (deg)	95.5146(17)	95.439(2)	95.1376(19)	95.463(2)	95.557(2)
γ (deg)	104.1725(18)	103.9133(19)	103.855(2)	104.2692(19)	104.228(2)
*V* (Å^3^)	13219.6(4)	13329.7(6)	12984.0(5)	13219.5(5)	13137.5(6)
ρ_calcd_ (g cm^–3^)	2.865	2.843	2.920	2.871	2.890
μ (mm^–1^)	16.422	16.340	16.831	16.585	16.748
reflns collected	99941	102581	88278	91644	92261
unique reflns (*R*_int_)	50681 (0.070)	51191 (0.098)	49686 (0.065)	50619 (0.058)	50321 (0.071)
observed reflns [*I* > 2σ(*I*)]	36150	28546	29139	32826	31503
params (restraints)	1557 (0)	1489 (18)	1523 (18)	1514 (12)	1543 (6)
*R*(*F*)^a^ [*I* > 2σ(*I*)]	0.066	0.097	0.082	0.063	0.064
*wR*(*F*^2^)^b^ [all data]	0.186	0.277	0.229	0.175	0.178
GoF	1.047	1.063	1.044	1.038	1.055

a *R*(*F*) = Σ||*F*_o_ – *F*_c_||/Σ|*F*_o_|.

b *wR*(*F*^2^) = {Σ[*w*(*F*_o_^2^ – *F*_c_^2^)^2^]/Σ[*w*(*F*_o_^2^)^2^]}^1/2^.

Thermal vibrations for heavy atoms (W, Ln, Br, K, and Si) were treated anisotropically. Hydrogen atoms of organic H_2_L ligands were placed in calculated positions using standard SHELXL parameters. Some of the anisotropic thermal ellipsoids from potassium atoms and the silicon atom in **1**-Tm were normalized using ISOR-type restraints from SHELXL. All compounds display significant disorder between potassium counterions and lattice water molecules, which prevents us from modeling all of the cation/solvent network. For the isostructural compounds **1**-Ln, 11 to 17 sites with appropriate geometries for K cations were located in Fourier maps. These occupancies were initially refined without restrictions and fixed to the first decimal in the last cycle, which results in a total number of 9.0 (**1**-Eu) to 12.3 (**1**-Tb) potassium atoms per each asymmetric unit containing three hybrid polyanions. Analogously, only 27 to 36 lattice water molecules were determined in the crystal structures of **1**-Ln. Large solvent accessible voids accounting for 17 to 25% of the unit cell volume can be found in the final structural solutions due to the severe structural disorder. According to PLATON, the largest voids are located at (i) *x*, *y*, *z* = 0, 0, 0 and occupy a volume of 3130 (**1**-Sm), 3028 (**1**-Dy), 2207 (**1**-Ho), 3277 (**1**-Er), 2432 (**1**-Tm), and 2737 Å^3^ (**1**-Yb); (ii) *x*, *y*, *z* = 0, 0.5, 0 and occupy a volume of 3423 (**1**-Gd) and 2293 Å^3^ (**1**-Lu); (iii) *x*, *y*, *z* = 0, 0.5, 0.5 and occupy a volume of 3335 Å^3^ (**1**-Eu); and (iv) *x*, *y*, *z* = 0, 1, 0.5 and occupy a volume of 2814 Å^3^ (**1**-Tb). Elemental and thermal analyses were essential to unequivocally determine the presence of 15 K ions and 42 hydration water molecules, that are five potassium and 14 water molecules per formula. The remaining cations and solvent molecules could well be located in these structural voids. It is worth noting that the presence of methanol solvent molecules was dismissed on the basis of elemental analyses. Furthermore, all the structures show large maxima of residual electron density, which are located close to the W atoms according to the final difference density map. Large residual maxima in the final Fourier map are a common fact found in the refinement of polyoxotungstate structures due to the high level of absorption of heavy atoms such as W.

## Results and Discussion

### Synthetic Aspects

Encouraged by the SMM behavior exhibited by both the Peacock–Weakley-type [Dy(β_2_-SiW_11_O_39_)_2_]^13–^ anion^40^ and the heterometallic [Zn(μ-L)(μ-OAc)Dy(NO_3_)_2_] complex^35^ based on the H_2_L ligand, we first explored the reactivity of Dy^III^ salts in our Ln^III^/H_2_L/POM synthetic system. Due to the low solubility of the organic ligand in water, a mixture of Dy(NO_3_)_3_ and H_2_L dissolved in methanol was reacted with a hot solution of K_8_[SiW_11_O_39_]·13H_2_O in aqueous 1 M NaOAc buffer (1:1:1 molar ratio). Taking into account the role that alkaline cations played as crystallizing species in some of our previous works on lanthanide-containing POMs,^64^ aqueous 1 M CsCl was also tested as a structural directing agent. The addition of Cs^+^ cations led to the formation of yellow single crystals (**2**-Dy) that were initially characterized by FT-IR spectroscopy. Vibrational bands originating from both the H_2_L and POM precursors can be observed in its IR spectrum which establishes the hybrid nature of the compound. Weak- to medium-intensity peaks in the 1100–1800 cm^–1^ range confirm the presence of the organic H_2_L ligand, whereas bands belonging to the POM framework can be observed in the inorganic region below 1000 cm^–1^ (Figure S4). The POM domain strongly resembles that of the precursor with small variations which consist of red shifts of 10 and 25 cm^–1^ of signals associated with ν_as_(W–O_a_–W) and ν_as_(W–O_t_) modes at ca. 705 and 950 cm^–1^, respectively. This fact indicates that FT-IR spectroscopy represents a straightforward tool to establish the functionalization of the parent cluster. Single-crystal X-ray diffraction experiments revealed the presence of hybrid [Dy(α-SiW_11_O_39_)(H_2_L)]^5–^ anions in the crystal structure of **2**-Dy.^65^ Unfortunately, the severe disorder in the cation/solvent network as a result of the simultaneous presence of (a) Na^+^ coming from the buffer, (b) K^+^ from the lacunary POM precursor, and (c) Cs^+^ from the crystallizing agent, did not allow us to crystallographically determine the amount of each species. We tried to ascertain this issue by performing ICP-MS analyses on different crystal batches, but the low reproducibility of results precluded us from reporting this compound as a pure phase.

To avoid the presence of so many different alkaline cations, we opted for selecting similar reaction conditions but using aqueous 1 M KOAc buffer instead. In this case, the solubility of the synthetic system drastically decreases in such a way that a significant amount of yellow precipitate is created and a colorless solution is obtained after filtering out this solid. These drawbacks were overcome by reducing the concentration of the buffer to 0.5 M. Although some yellow solid is still formed in the reaction, slow evaporation of the final solution at room temperature affords crystals of **1**-Dy. The FT-IR spectra recorded for crystals of **1**-Dy and yellow precipitates are virtually identical to that observed for the **2**-Dy phase (Figure S5). In contrast, the low reaction yield obtained for crystals of **1**-Dy encouraged us to make use of powder X-ray diffraction (PXRD) experiments to determine whether both powdered and crystalline samples correspond to the same phase.

The experimental pattern collected for freshly filtered crystals of **1**-Dy compares well with the pattern-matching procedure carried out with single-crystal X-ray diffraction data, which suggests that samples are constituted by a single crystalline phase. Unfortunately, yellow precipitates display diffraction patterns with only a couple of poorly resolved diffraction maxima, which evidence the nearly amorphous nature of the solid (Figure S6). Although efficient functionalization can be inferred from FT-IR spectroscopy, this fact does not allow us to crystallographically characterize the powdered sample, and thus, only crystals of **1**-Dy were used in further studies. Additional experiments revealed that the counterion of the Dy^III^ salt (nitrate vs chloride) does not affect either the nature of the final product or the reaction yield.

Synthetic studies were later extended to other lanthanide(III) cations. It is well-known that lanthanide-containing POM assemblies are highly dependent on the size of the 4f metal. Frequently, architectures obtained for early lanthanides considerably differ from those comprising smaller 4f metals.^14^ In our case, the use of mid-to-late lanthanides affords the isostructural **1**-Ln (Ln = Sm to Lu) salts (Figure S7). Nevertheless, early lanthanides (La to Nd) yield Peacock–Weakley-type [Ln^III^(SiW_11_O_39_)_2_]^13–^ anions under similar synthetic conditions as determined by FT-IR spectroscopy^48^ (Figures S8 and S9).

### Crystal Structure

**Figure 1:**
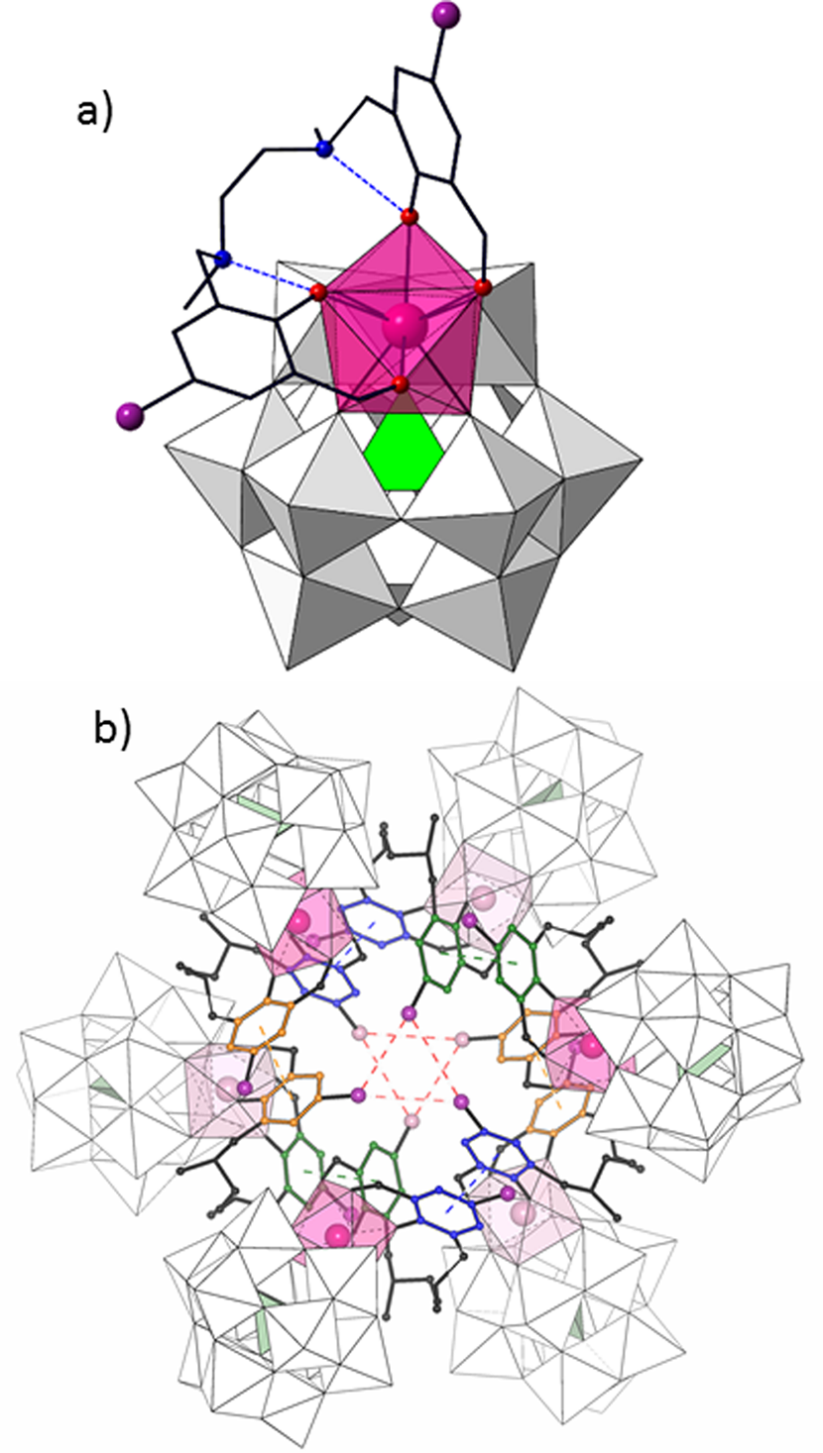
(a) Molecular structure of hybrid [Ln(H_2_L)(α-SiW_11_O_39_)]^5–^ ({Ln}) POM found in compounds **1**-Ln. Intramolecular N–H···O hydrogen bonds are represented as blue dashed lines. (b) Hexameric chairlike supramolecular assemblies in **1**-Ln. Aromatic rings involved in π–π stacking interactions that belong to contiguous {Ln} units are depicted in the same color. Intermolecular Br···Br contacts are represented as red dashed lines Color code: WO_6_, white; SiO_4_, green; LnO_8_, pink; C, black, O, red; N, blue; Br, purple.

All compounds **1**-Ln are isostructural and crystallize in the triclinic *P*1̅ space group containing three crystallographic independent molecular [Ln(H_2_L)(α-SiW_11_O_39_)]^5–^ ({Ln}) clusters in each asymmetric unit (*Z* = 6). Hybrid species are composed of a trivalent Ln^III^ cation coordinated by four oxygen atoms that belong to two aldehyde (O_a_) and two phenoxy (O_p_) groups from the H_2_L ligand and the four oxygen atoms delimiting the vacant site of the monolacunary [α-SiW_11_O_39_]^8–^ Keggin-type fragment (O_POM_). The organic ligand adopts a tetradentate-O_4_ mode leaving the inner N_2_O_2_ site available for the incorporation of additional metal cations (Figures 1a and S10). Coordination of phenoxydo oxygen atoms to the lanthanide center promotes the migration of protons to amine groups, and the resulting zwitterionic form is stabilized by a pair of intramolecular N–H···O-type hydrogen bonds established between protonated amine groups and deprotonated phenolate oxygen atoms (Table S2). Although related monolanthanide complexes of aminophenolic Mannich-base compartmental ligands are scarcely found in the literature,^66^ they show a similar zwitterionic arrangement.

Close inspection to the Cambridge Structural Database (CSD)^67^ reveals that this coordination mode makes the H_2_L ligand adopt an unusual conformation in comparison to all the metal complexes reported to date, and it confirms that species reported herein represent the first examples of mononuclear lanthanide complexes with this specific ligand. Geometrical parameters of 59 crystallographically independent ligands belonging to 42 structures have been determined. These can be classified into three groups: (i) heterometallic dinuclear Ln–M^II^ complexes (M^II^ = Cu, Zn); (ii) heterometallic dinuclear Ln–M^II^ complexes (M^II^ = Co, Ni); and (iii) sandwich-type M^II^–Ln–M^II^ species (M^II^ = Cu, Zn) with more than one H_2_L ligand. The scatter plot of intramolecular centroid···centroid distances between aromatic rings from the same ligand versus angles between planes which contain those rings (Figure S11 and Table S3) allows these three groups to be easily distinguished. Dihedral angles in the 155–173° range are found for the heterometallic complexes belonging to the first group, whereas this angle slightly decreases in the case of sandwich-type complexes (145–164°). Thus, it can be concluded that members from both families display quasi-coplanar aromatic rings. In contrast, smaller dihedral angles of ca. 110° are found when 3d metal ions are either Ni or Co, but intramolecular centroid···centroid distances are similar in all three groups (ca. 8 Å). For comparison, compounds **1**-Ln exhibit even smaller angles ranging from 84 to 95°, in such a way that both rings are no longer coplanar, because the empty N_2_O_2_ pocket allows the ligand to be considerably folded. This makes the aromatic rings close to each other with intramolecular centroid···centroid distances of only ca. 6.5 Å. It is worth noting that one of the aromatic groups is near (average twisting angle of ca. 15°) the ideal mirror plane of the Keggin-type monolacunary anion with *C*_*s*_ point symmetry (Figure S12). This configuration might be promoted by supramolecular interactions established between contiguous {Ln} units.

**Figure 2:**
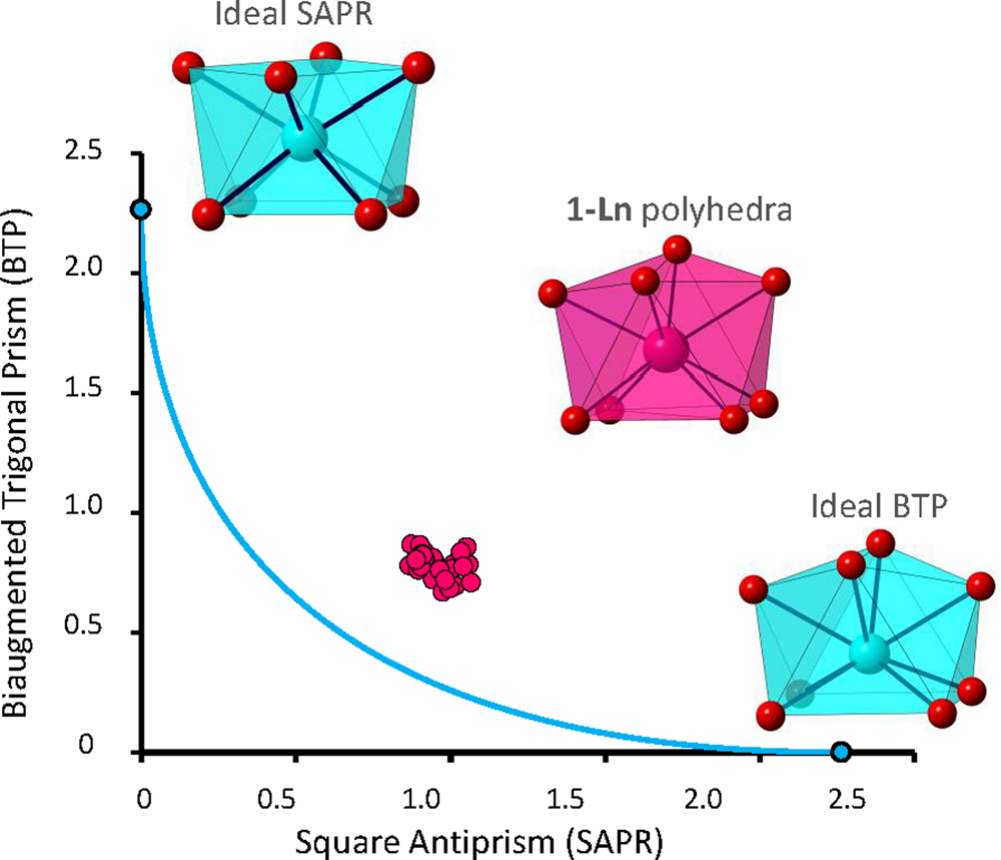
Biaugmented trigonal prism (BTP) vs square antiprism (SAPR) shape map for the LnO_8_ coordination polyhedra of 4f ions in **1**-Ln. Solid line: minimal distortion pathway between reference shapes.

Lanthanide centers exhibit distorted eight-coordinated geometries that have been analyzed by continuous shape measures (CShM). Similar CShM values have been obtained in all cases using both a biaugmented trigonal prism (BTP, *C*_2*v*_: 0.67–0.87 range) and square antiprism (SAPR, *D*_4*d*_: 0.87–1.06 range) as reference shapes (Table S4). Comparison with other eight-coordinated reference polyhedra affords higher CShM values (above 1.4). All of the lanthanide coordination polyhedra reported in this work have been scattered in the BTP vs SAPR shape map (Figure 2) to determine whether they follow the trend marked by the minimal distortion pathway between the two reference polyhedra. Low path deviation values in the 0.19–0.29 range, far from the upper limit of 0.5 selected by Casanova et al.,^63^ confirm the best description of coordination geometries as biaugmented trigonal prisms distorted toward square antiprismatic. This type of geometry arises from the out-of-pocket coordination mode of 4f metals toward the Keggin-type monolacunary skeleton. The Ln–O bond lengths (Table S5) follow the order Ln–O_p_ < Ln–O_POM_ < Ln–O_a_, with average values in the 2.21–2.36, 2.27–2.45, and 2.31–2.56 Å range, respectively. As observed in some other series of lanthanide containing POMs,^64^ a subtle linear shortening of about 0.1 Å can be observed in these bond lengths as the atomic number of the 4f cations increases, in good agreement with the well-known lanthanide contraction effect (Figure S13). This trend is more pronounced for Ln–O_a_ and Ln–O_p_ bonds, in comparison to that belonging to Ln–O_POM_ bonds.

With regard to the crystal packing, six molecular {Ln} hybrid POMs self-assemble into supramolecular structures in a chairlike conformation via π–π and Br···Br interactions (Figure S14). The virtual chair formed by lanthanide atoms displays the shortest Ln···Ln distances in the 7.981(6) to 8.244(9) Å range. Aromatic rings from contiguous units contribute to the π–π stacking, which display centroid-to-centroid distances ranging from 3.52(2) to 3.618(11) Å (Table S6). Additionally, only Br atoms that belong to aromatic groups parallel to the ideal symmetry plane are facing the interior of the hexamer. These six atoms correspond to alternate {Ln} units and are disposed in two different planes with interplanar distances of about 9 Å (Figure 1b). The 3.6465(4)–4.086(3) Å bond lengths found for the bifurcated Br···Br type contacts are in line with those observed for analogous interactions in the literature (Table S7).^68^

### Magnetic Properties

**Figure 3:**
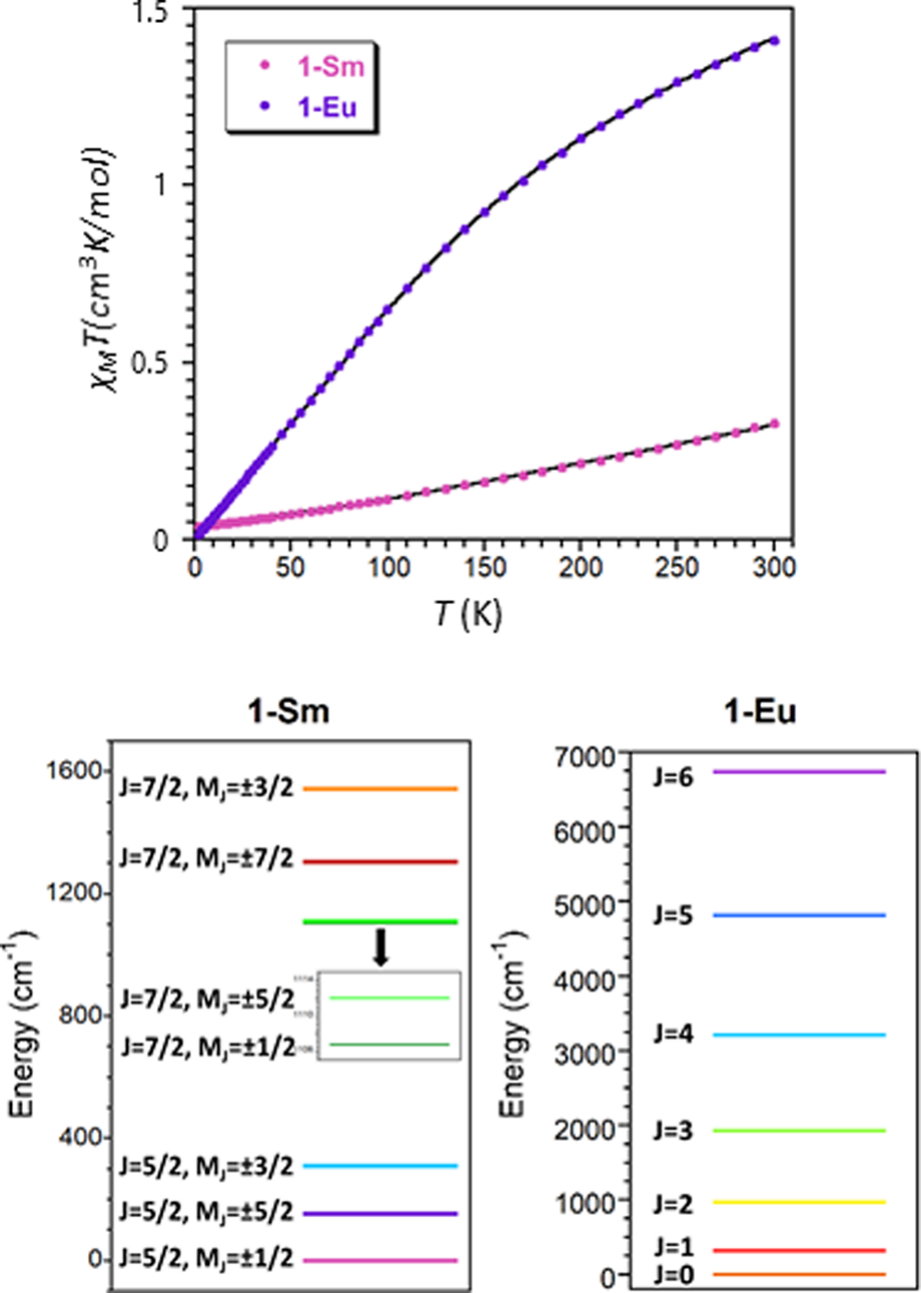
Top: temperature dependence of the χ_M_*T* product at 1000 Oe for **1**-Sm and **1**-Eu. Black solid lines represent the best fit to the magnetic data. Bottom: Energy level diagrams for **1**-Sm (left) and **1**-Eu (right).

**Figure 4:**
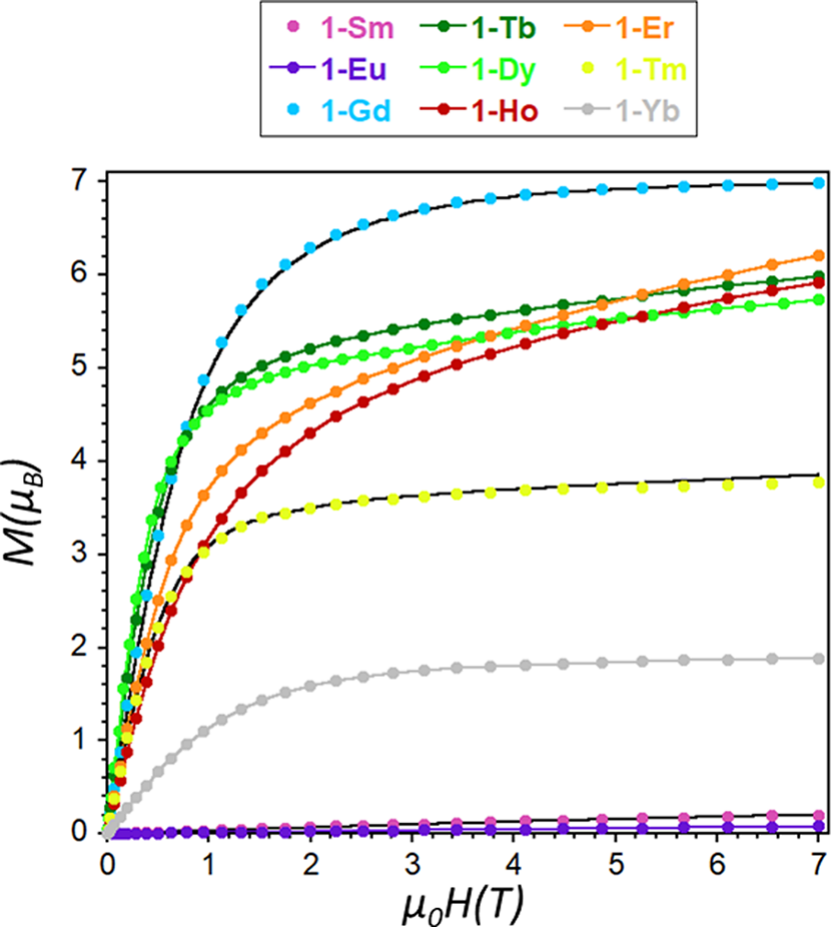
Field dependent magnetization plots at 2 K for complexes **1**-Ln. The black lines represent the fittings discussed in the text. The rest of the lines are a guide for the eye.

**Figure 5:**
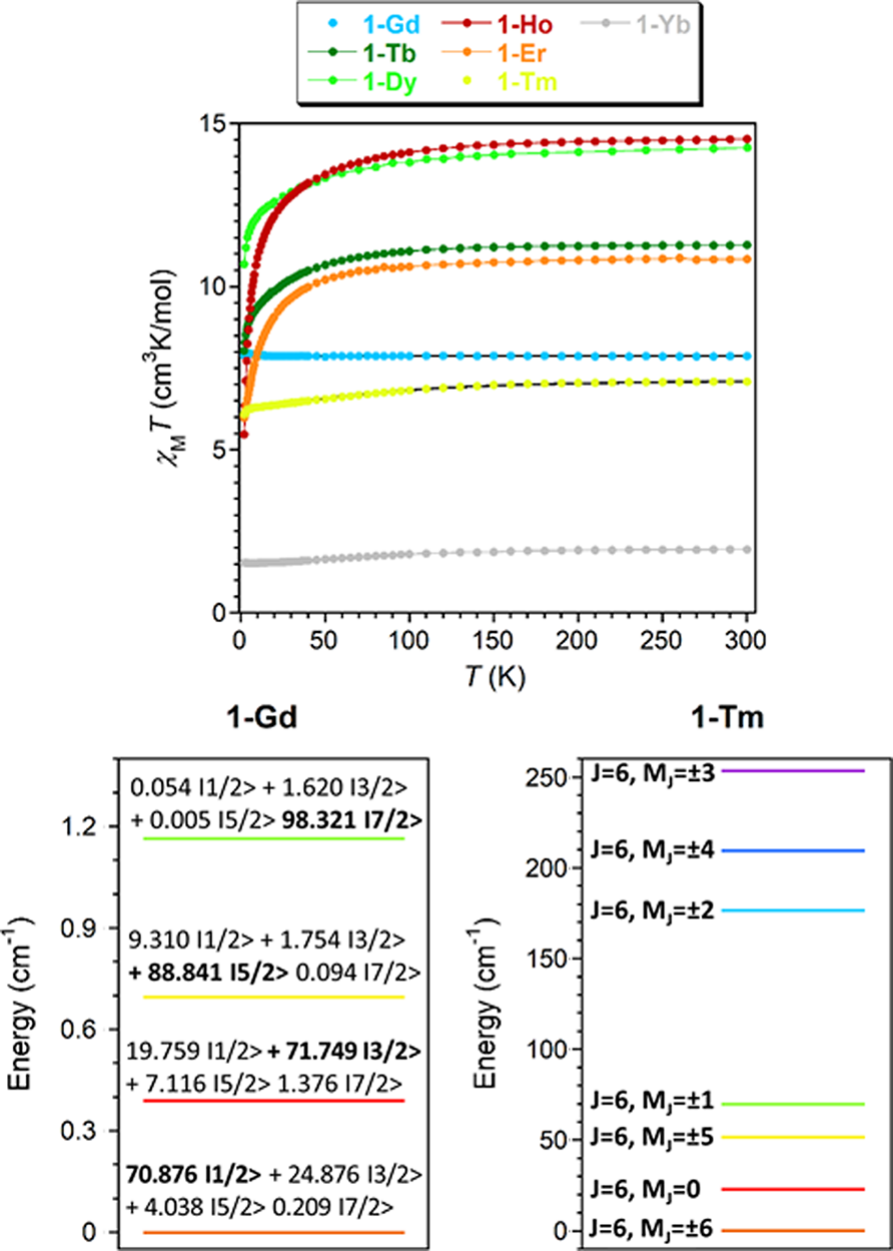
Top: temperature dependence of the χ_M_*T* product at 1000 Oe for complexes **1**-Ln. The black line represents the fitting discussed in the text, and the rest of the lines are a guide for the eye. Bottom: Energy level diagrams for **1**-Gd and for **1**-Tm.

Due to the potential of Ln-substituted POMs to behave as SMMs, the magnetic properties of **1**-Ln were studied in detail and are given in Figures 3–5 and S15–S18. First, the χ_M_*T* product (where χ_M_ is the molar susceptibility per Ln^III^ atom) for **1**-Sm is 0.33 cm^3^K/mol at 300 K, which is higher than the expected value of 0.09 cm^3^K/mol for a free ion with *J* = 5/2 and *g* = 2/7 (Figure 3). The observed behavior can be ascribed to the presence of thermally populated excited states, which contribute to the magnetic susceptibility. Upon cooling, the χ_M_*T* product decreases continuously to reach a value of 0.04 cm^3^ K/mol at 2 K. Field dependent magnetization measurements (*H* = 0–7 T, *T* = 2–10 K) reveal a clear dependence of the curves with temperature, in which the highest magnetization value is obtained at the lowest measured temperature and is 0.21 μ_B_ (Figure 4). This value is lower than the value expected for a free Sm^III^ ion (0.71 μ_B_), probably due to (a) the splitting of the ground state by crystal field effects and (b) second order Zeeman effects derived from the mixing of the ground and first excited states.^69^ The consideration of crystal field effects in the magnetic behavior of **1**-Sm leads to a Hamiltonian that contains nine crystal field parameters (*B*_2_^0^, *B*_2_^2^, *B*_4_^0^, *B*_4_^2^, *B*_4_^4^, *B*_6_^0^, *B*_6_^2^, *B*_6_^4^, and *B*_6_^6^, *C*_2*v*_ approximate symmetry),^70^ which can be simplified into the following equation to avoid overparametrization:
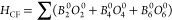
1where the *O*_*k*_^*q*^ terms are equivalent Stevens operators, which are a function of the angular moments (e.g., O_2_^0^ = 3L_*z*_^2^–L^2^). Thus, eq 1 only considers the axial distortions and neglects transversal operators.^71^ The magnetic susceptibility and magnetization data were simultaneously fitted by using the Phi program^72^ to the above equation (Figures 3 and S15 and S16), in which the spin–orbit coupling constant was fixed to the 281 cm^–1^ value estimated from luminescence studies (see Photoluminescent Properties section). The best fit of the data yielded *B*_2_^0^ = 4.997, *B*_4_^0^ = −0.243, and *B*_6_^0^ = −0.00389 cm^–1^, which suggest that *M*_J_ = ± 1/2 is the ground state and that the *M*_J_ = ± 5/2 and *M*_J_ = ± 3/2 excited states are located 153 and 310 cm^–1^ above the ground state, respectively (Figure 3). These results are comparable to those calculated from luminescence spectra, which placed the energy levels of the first excited states at 125 and 272 cm^–1^, respectively. Additionally, the first doublet of the first excited state (*J* = 7/2, *M*_J_ = ± 1/2) is located 1107 cm^–1^ above the ground state.

When it comes to **1**-Eu, similarly to **1**-Sm, the χ_M_*T* product at 300 K (1.41 cm^3^K/mol) is higher than the expected value (0 cm^3^K/mol), which is due to the presence of thermally populated excited states (Figure 3). Upon cooling, the χ_M_*T* product decreases down to 0.016 cm^3^ K/mol at 2 K, which is in good agreement with an increase in the population of the *J* = 0 state. The χ vs *T* plot displays typical Van Vleck paramagnetism below 100 K (Figure S15),^73^ but below 10 K a small paramagnetic contribution is detected, which is most likely due the presence of small quantities of Eu^II^ (*J* = 7/2). The magnetic susceptibility curves can be well modeled by an equation proposed by Khan^74^ that correlates the energy of the *J* states with the spin–orbit coupling parameter (λ), affording λ = 321 cm^–1^ and δ = 0.04% (δ being the percentage of Eu^II^ impurities). This value is in good agreement with that subtracted from luminescent measurements (λ = 310 cm^–1^, see Photoluminescent Properties section) and suggests that the first excited state is located around 321 cm^–1^ above the ground state (*J* = 0, Figure 3), which implies that the magnetic contribution of that first excited state cannot be neglected. On the other hand, magnetization measurements between 2 and 10 K reveal temperature independent curves, which reach a value that is very close to 0.084 μ_B_ at 7 T at all of the studied temperatures (Figures 4 and S16). The dependence with the field is practically linear, and no saturation is observed.

**1**-Gd displays a nearly constant χ_M_*T* value of ∼7.9 cm^3^K/mol with a maximum at around 4 K (7.98 cm^3^K/mol, Figure 5), which is most likely due to the presence of impurities (note that the shortest Gd···Gd distances are of ∼8 Å and that the metal centers are well isolated in the crystal lattice). The magnetization saturates at very low temperatures and high fields, as expected for noninteracting Gd^III^ ions (Figure 4, Table 2). In order to gain insights into this compound, we recorded the Q-band EPR spectra at room temperature (Figure S17). The best fitting to the spectrum yielded *D* = 0.0802 cm^–1^, *E* = 0.0231 cm^–1^, *g*_*x*_ = 1.990, *g*_*y*_ = 1.986 and *g*_*z*_ = 1.985. The values of *D* and *E* parameters are in good agreement with those found in the literature^75^ and reproduce fairly well the susceptibility and field dependent magnetization curves when *g* = 2 (Figures 5 and S16). Thus, according to these fittings, the ground state is fundamentally constituted by the ±1/2 doublet (71%) with a significant contribution of the ±3/2 doublet (25%) and a minor contribution of the ±5/2 doublet (4%). The first excited state is only 0.39 cm^–1^ above the ground state (Figure 5).

**Table 2:** Direct Current Magnetic Data for **1**-Ln

	ground-state of Ln^III^ ion	χ_M_*T* theor.^a^ 300 K/exptl. 300 K/exptl. 2K (cm^3^K/mol)	M theor.^b^/exptl. 2 K and 7 T (μ_B_)
**1**-Sm	^6^H_5/2_, *g*_J_ = 2/7	0.09/0.33/0.04	0.71/0.21
**1**-Eu	^7^F_0_, *g*_J_ = 0	0/1.41/0.016	0/0.08
**1**-Gd	^8^S_7/2_, *g*_J_ = 2	7.88/7.88/7.90	7/6.99
**1**-Tb	^7^F_6_, *g*_J_ = 3/2	11.82/11.28/8.04	9/5.99
**1**-Dy	^6^H_15/2_, *g*_J_ = 4/3	14.17/14.24/10.70	10/5.41
**1**-Ho	^5^I_8_, *g*_J_ = 5/4	14.07/14.53/5.48	10/5.92
**1**-Er	^4^I_15/2_, *g*_J_ = 6/5	11.48/10.85/5.99	9/6.22
**1**-Tm	^3^H_6_, *g*_J_ = 7/6	7.14/7.09/6.10	7/3.77
**1**-Yb	^2^F_7/2_, *g*_J_ = 8/7	2.57/1.95/1.54	4/1.89

a χ_M_*T* = [*N*β^2^]/[3*k*]{*g*_*j*_^2^*J*(*J* + 1)}.

b *M*_s_ = *g*_J_*JN*μ_B_.

The χ_M_*T* products of the remaining compounds are close to the expected values for independent Ln^III^ ions in the free-ion approximation, but they decrease continuously upon lowering the temperature and abruptly below ∼100 K in most cases (Figure 5, Table 2). The deviation from the Curie behavior is mainly due to the depopulation of the *M*_J_ sublevels of the Ln^III^ ions. While the statistical population of the *M*_J_ sublevels of the ground term leads to the fulfilment of the free-ion approximation at room temperature, the splitting of the ground term by the ligand field at low temperatures results in a decrease in the χ_M_*T* values. Such splitting and the resulting magnetic anisotropy are also responsible for the low magnetization values observed at 2 K and 7 T (Figures 4 and S16, Table 2).

Interestingly, the temperature dependence of the magnetic susceptibility and magnetization curves of **1**-Tm can be simultaneously fitted considering eq 1 and assuming λ = 1314 cm^–1^, as in the free-ion (note that the transversal components of the crystal field have not been considered to avoid overparametrization, as for **1**-Sm). The best fitting with the Phi program leads to *B*_2_^0^ = −1.529, *B*_4_^0^ = −0.0345, and *B*_6_^0^ = 0.000964 cm^–1^ (Figures S18 and S19). These values suggest that the ground state is *M*_J_ = ± 6 and that the *M*_J_ = 0 state is approximately 23 cm^–1^ above it, being the ±5, ±1, ±2, ±4, and ±3 states at 51.8, 69.8, 176.5, and 209.3 cm^–1^, respectively (Figure 5). Even though the use of the same equation does not lead to a satisfactory fitting for the remaining compounds, the distribution of the energy levels found for **1**-Tm is similar to those observed in the literature for other Tm-based compounds.^76^

**Figure 6:**
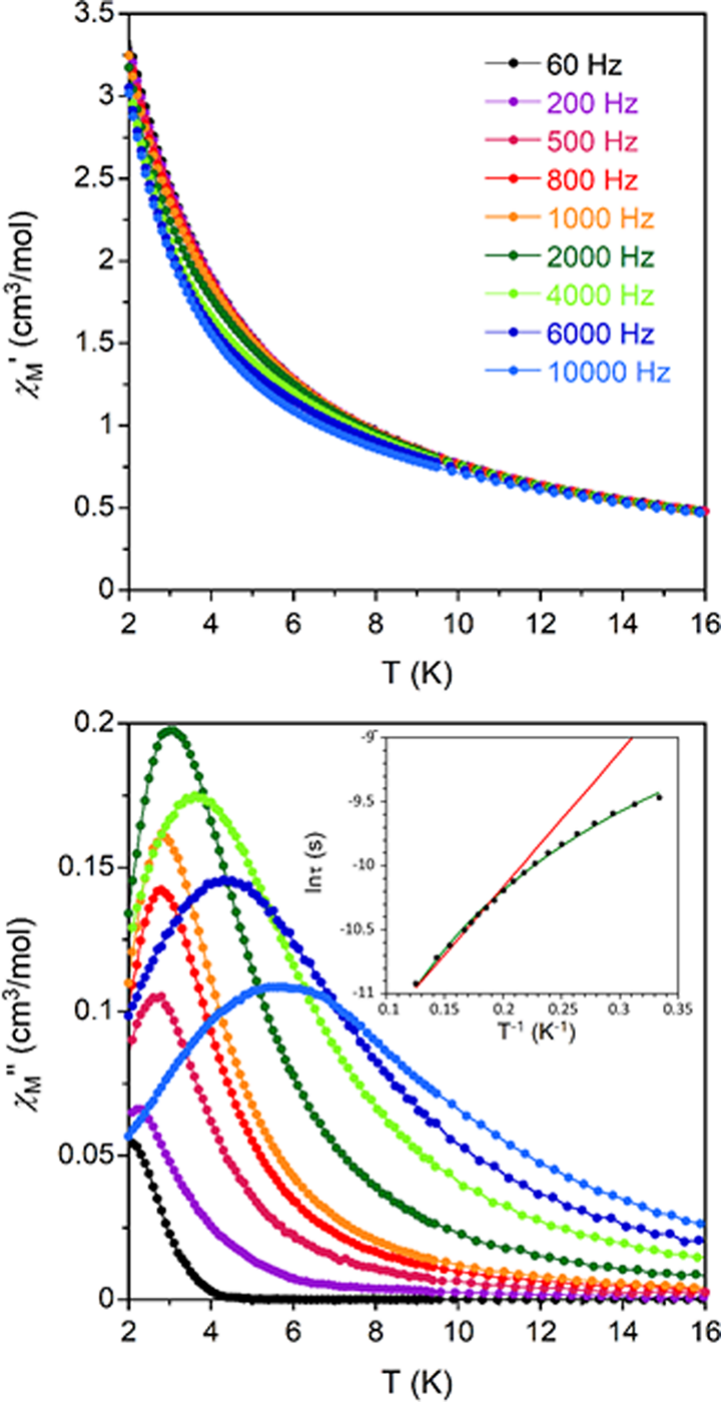
Temperature dependence of the in-phase (χ_M_′, top) and out-of-phase (χ_M_″, bottom) components of the ac susceptibility for **1**-Gd under an external field of 1000 Oe. Inset: Arrhenius plot of relaxation times of **1**-Gd (red line) and best fit to eq 4 (green line).

**Figure 7:**
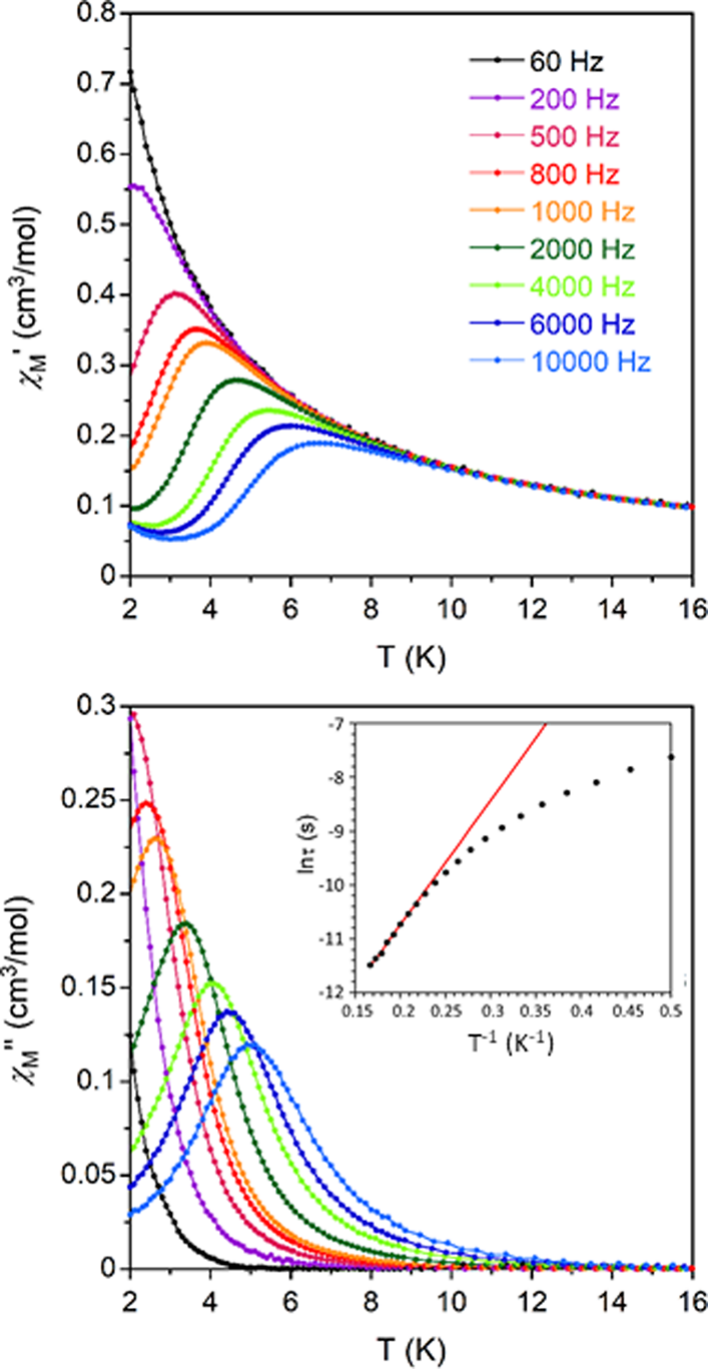
Temperature dependence of the in-phase (χ_M_′, top) and out-of-phase (χ_M_′, bottom) components of the ac susceptibility for **1**-Yb under an external field of 1000 Oe. Inset: Arrhenius plot of relaxation times of **1**-Yb (red line).

Alternating current magnetic measurements revealed that **1**-Gd and **1**-Yb display well-marked maxima in the out-of-phase susceptibility (χ_M_″) signals below 6 K in the presence of an external magnetic field of 1000 Oe (no contribution to χ_M_″ was observed at *H* = 0), implying the occurrence of slow relaxation of magnetization (Figures 6 and 7). The absence of similar peaks in **1**-Tm is not surprising, as the fact that the first excited state is *M*_J_ = 0 (Figure 5) prevents **1**-Tm from behaving as an SMM.^77^ On the other hand, there are not clear shortest Ln–O distances in the LnO_8_ coordination polyhedra, and therefore, lanthanides with oblate electron density such as Dy and Tb do not display an appropriate ligand field as to favor an axial ground state and, hence, the SMM behavior. Thus, **1**-Dy displays χ″ signals with no maxima above 2 K (Figure S20) and little frequency dependence.^34^

In order to gain insights into the relaxation mechanisms, the frequency-dependences of in-phase and out-of-phase magnetic susceptibilities and the related Argand (Cole–Cole) plots were simultaneously fitted to the Debye model (Figures S21–S23), optimizing the relaxation times (τ), the distribution of relaxation times (α), and isothermal and adiabatic susceptibilities (χ_T_ and χ_S,_ respectively).^78,79^ The Cole–Cole plots of **1**-Gd and **1**-Yb afford α values in the 0.13 (2 K)–0.03 (6 K) and 0.04 (2 K)–0.06 (4.4 K) ranges, respectively. These values together with the deviation of relaxation times from linearity indicate that the relaxation of the magnetization does not occur exclusively through an Orbach mechanism (eq 2). In fact, the *U*_eff_ (energy barrier) value obtained for **1**-Gd (7.23 cm^–1^) is too large for a rather isotropic ion where the energy difference between the ground and first excited states is ∼0.39 cm^–1^ according to the parameters extracted from EPR measurements and direct current magnetic data (see above, note that the Zeeman splitting of the ground state is even lower at 1000 Oe). Moreover, the relaxation times follow a power law (eq 3), which leads to an *n* = 1.54 value. Such a value, significantly lower than the value expected for a Raman mechanism (*n* = 7 or 9), is between the values expected for direct processes (*n* = 1) and phonon bottleneck effects (*n* = 2), suggesting that both mechanisms could be responsible for the slow magnetic relaxation observed in **1**-Gd.^80^ The application of higher external fields (*i.e.*, 2000 and 3000 Oe) leads to a shift in the maximum of the χ_M_″ vs *T* curves, in agreement with the presence of a direct mechanism (Figure S24). Additionally, the relaxation times reach saturation at lower temperatures upon increasing the external magnetic field (Figure S25 and Table S8), suggesting that QTM could be operative under certain fields. Therefore, this effect has also been considered in the final fitting of relaxation times, which follows eq 4 (inset in Figure 6). On the other hand, the *U*_eff_ value of 14.8 cm^–1^ obtained for **1**-Yb is significantly lower than the separation between the ground and first excited states, which is 261 cm^–1^ according to the energy difference of the first two peaks in the photoluminescence spectra (see below). Thus, a Raman relaxation process could take place in this compound, as already reported in the literature for related Yb^III^-based compounds.^35^
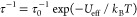
2
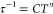
3
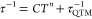
4

### Photoluminescent Properties

Both inorganic POM fragments and the H_2_L ligand have proved to act as antenna ligands to sensitize weak-emitting 4f metal centers.^11,35^ Furthermore, an efficient emission could be *a priori* expected in the case of **1**-Ln derivatives because of the absence of any aqua ligands coordinated to lanthanide centers responsible for quenching the fluorescence through deactivation of excited states via high frequency O–H oscillators. Therefore, solid state photophysical properties were evaluated for all of the **1**-Ln series: Sm to Dy and Tm derivatives in the visible region and Ho, Er, and Yb in the near-infrared region (NIR). As shown in the UV–vis diffuse reflectance spectrum of the H_2_L ligand, absorption in the 220–380 nm region displays two maxima at 250 and 360 nm which correspond to the π–π* aromatic ring and *n*–π* aldehyde group transitions, respectively, whereas the K_8_[α-SiW_11_O_39_]·13H_2_O POM precursor exhibits a strong absorption below 310 nm. The related absorption in compounds **1**-Ln, as exemplified by the **1**-Gd derivative, displays two signals centered at ca. 280 and 375 nm (Figure S26), and thus, samples were irradiated at these two different wavelengths using a Xe arc lamp as an excitation source.

**Figure 8:**
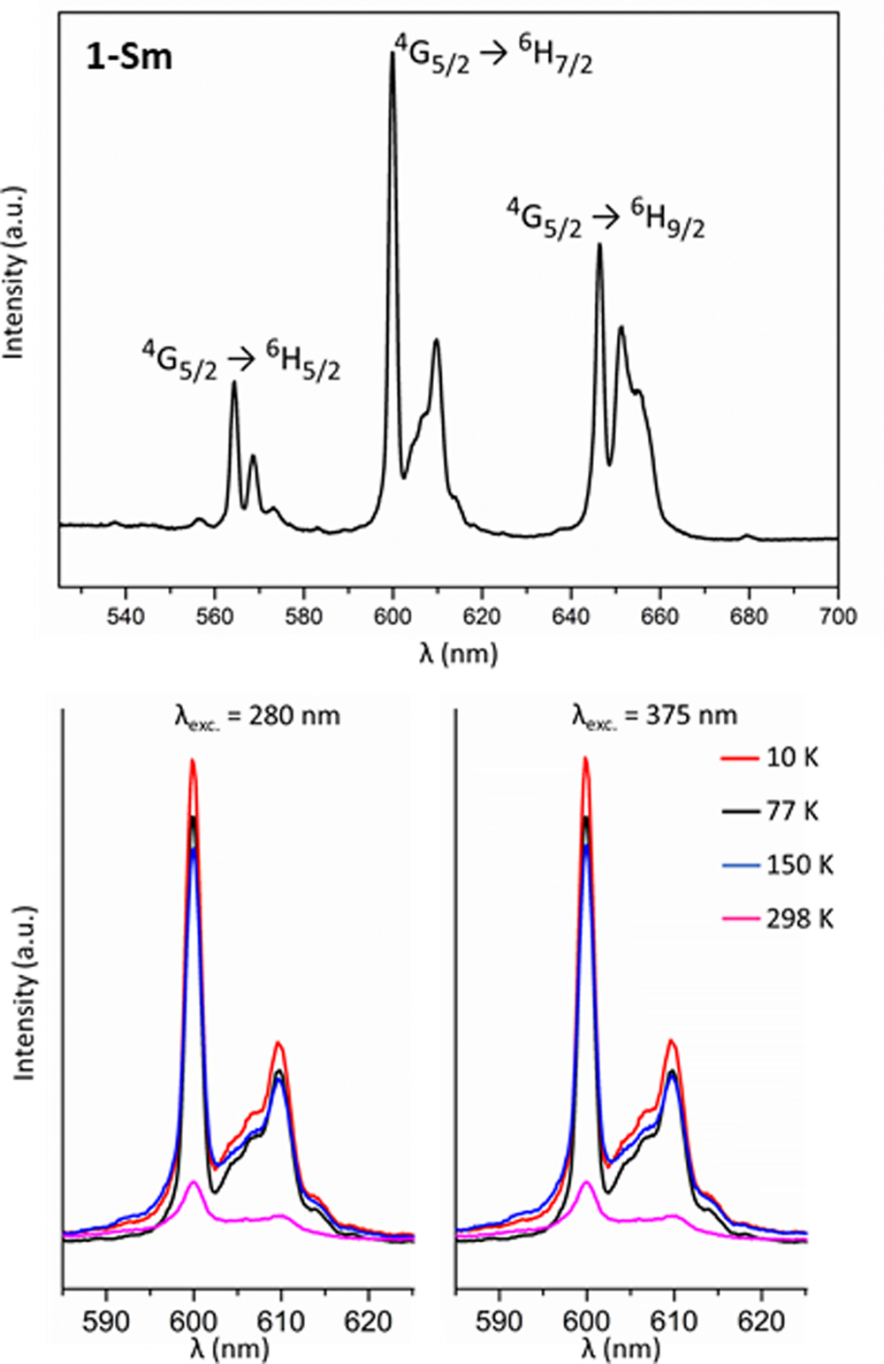
Top: Solid state photoluminescence spectrum of **1**-Sm recorded at 10 K upon excitation at 375 nm. Bottom: Thermal evolution of the most intense transition upon excitation at 280 and 375 nm.

Bright orange-reddish and red photoluminesce (Figure S27) were observed for **1**-Sm and **1**-Eu derivatives, respectively, and their emission spectra were recorded at 10 K, 77 K, 150 K, and room temperature. Low temperature excitation spectra (Figure S28) acquired for their more intense emission lines (600 nm for **1**-Sm and 614 nm for **1**-Eu) show a broad band in the 300–450 nm region, which is overlapped with some narrow bands arising from the intra-f^n^ transitions of the lanthanide ions. The high intensity of the former implies a more efficient luminescence sensitization via the excited states of the ligands, which is indicative of an antenna effect. When it comes to emission spectra, modification of the excitation wavelength does not alter either the position or the fine structure of the bands arising from the splitting of *J* levels by the crystal field. Moreover, signals are broadened and their intensity decreases as temperature increases due the higher kinetic energy, which promotes the radiationless thermal deactivation of excited states. Close inspection reveals that the intensity of the signals is similar for the spectra registered at 10 and 77 K, and in turn, it undergoes a drastic decrease above 150 K. In the case of **1**-Sm, the spectrum (Figure 8) shows three groups of signals with maxima at ca. 568, 600, and 646 nm, which correspond to ^4^G_5/2_ → ^6^H_*J*_ (*J* = 5/2, 7/2 and 9/2, respectively) transitions.^81^ At low temperatures, the spectra are dominated by the electric-dipole transition ^4^G_5/2_ → ^6^H_7/2_, whereas the ^4^G_5/2_ → ^6^H_9/2_ becomes the most intense signal at room temperature. The average spin–orbit coupling parameter (λ) can further be estimated from the energy difference between the centers of the emission bands, yielding a value of 280 cm^–1^.

**Figure 9:**
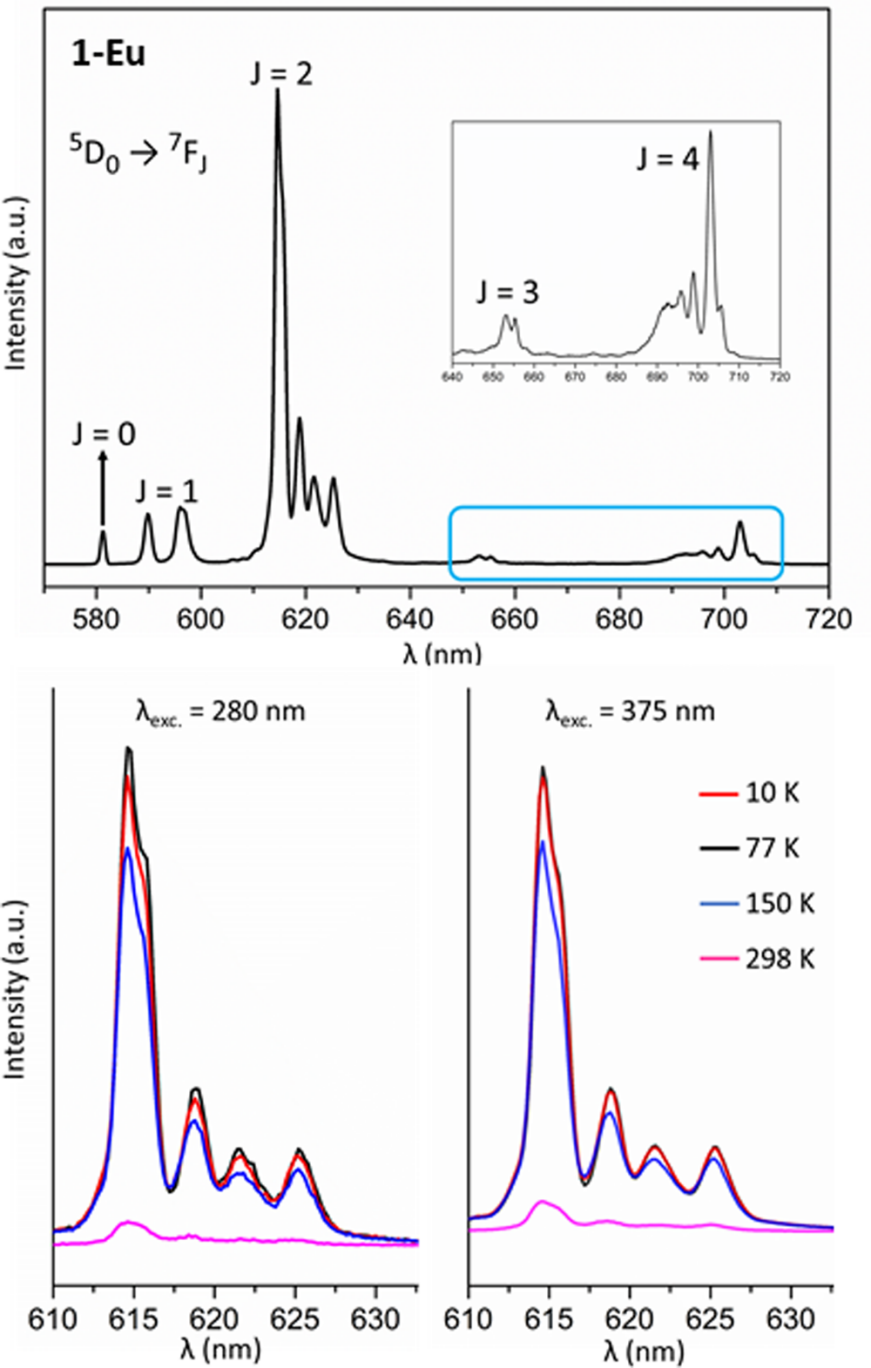
Top: Solid state photoluminescence spectrum of **1**-Eu recorded at 10 K upon excitation at 375 nm. Inset: detailed view of the weak transition bands. Bottom: Thermal evolution of the most intense transition upon excitation at 280 and 375 nm.

With respect to **1**-Eu, five groups of signals with peak maxima at 581, 596, 615, 653, and 703 nm, which can be assigned to characteristic ^5^D_0_ → ^7^F_*J*_ (*J* = 0, 1, 2, 3, and 4) transitions can be found in its spectrum (Figure 9). The crystal field effect allows the forbidden ^5^D_0_ → ^7^F_0_ and ^5^D_0_ → ^7^F_3_ transitions to be observed. The considerable intensity of the former evidences that the Eu^III^ ion occupies a low symmetry site,^82^ in good agreement with the biaugmented trigonal prismatic geometry determined by single-crystal X-ray diffraction studies for the {EuO_8_} moiety. The most dominant band corresponds to the hypersensitive electric dipole ^5^D_0_ → ^7^F_2_ transition, which displays considerably higher intensity (8:1) in comparison to the magnetic dipole allowed ^5^D_0_ → ^7^F_1_. The intensity of the former band increases with the local asymmetry. This fact can be inferred when comparing the low symmetry of the {EuO_8_} polyhedron in **1**-Eu, with the analogous square antiprismatic (*D*_4*d*_) center in the archetypic [EuW_10_O_39_]^9–^. In this line, the most intense ^5^D_0_ → ^7^F_2_ transition results in bright red luminescence for **1**-Eu, whereas the Na_9_[EuW_10_O_39_]·14H_2_O salt emits orange fluorescence^25^ due to the higher relative intensity of the ^5^D_0_ → ^7^F_1_ transition. The average λ = 310 cm^–1^ value extracted from the spectrum is close to that calculated from magnetic susceptibility curves.

Emission decay curves of the most intense lines for both compounds, that is, ^4^G_5/2_ → ^6^H_7/2_ at 600 nm (**1**-Sm) and ^5^D_0_ to ^7^F_2_ → 614 nm (**1**-Eu), were monitored as a function of temperature upon excitation at 280 and 375 nm. Experimental curves were fitted to a single exponential function *I* = *A*_0_ + *A*_1_ exp(−*t*/τ) in the case of **1**-Sm, whereas two decay components were found in the decay curves of **1**-Eu, and hence, they were fitted to a double exponential function, *I* = *A*_0_ + *A*_1_ exp(−*t*/τ_1_) + *A*_2_ exp(−*t*/τ_2_) (τ_*n*_, luminescence lifetimes; *A*_0_, background; *A*_*n*_, weighting parameters), where the second term accounts approximately for 75–80% of the total process. Luminescence lifetimes proved to be almost the same regardless of the excitation wavelength. (Table 3, Figures S29 and S30). The fitting results showed luminescence lifetimes of ca. 25 μs for **1**-Sm below 150 K, which suggest the absence of any thermally activated nonradiative deactivation process, but it drastically drops at room temperature. In contrast, lifetime values remain almost constant at 700 μs for the principal decay component of **1**-Eu at different temperatures. The observed lifetimes are in line with those found in the literature for related complexes with eight coordinated Sm^III^ and Eu^III^ ions.^21^

**Table 3:** Luminescence Lifetimes of Compounds **1**-Sm and **1**-Eu upon Excitation at 375 nm at Different Temperatures

temperature (K)	τ_exp_ **1**-Sm (μs),	τ_exp_ **1**-Eu (μs),
	Em. 600 nm	Em. 614 nm
10	25.11(6)	226(4)/735(4)
77	25.57(7)	195(3)/735(3)
150	26.61(6)	227(4)/796(3)
298	8.70(7)	181(3)/629(4)

Compounds **1**-Tb, **1**-Dy, and **1**-Tm display very weak ligand centered emission, which suggests an inefficient energy transfer from the ligands to lanthanide centers. To further characterize this phenomenon, the latter derivatives were irradiated with a 325 nm HeCd continuous laser and their spectra recorded at 20 K, together with that of the **1**-Gd derivative (Figure S31). The spectrum of **1**-Gd presents a broad band ranging from 450 to 650 nm and centered at 550 nm that could probably be ascribed to the emission from the first excited triplet level from the organic ligand to the ground level (ca. 18180 cm^–1^). The exited levels of Gd^III^ ions (ca. 315 nm) usually have higher energy than those of the ligand, and hence, the ligand-to-metal energy transfer is disabled. This allows the direct observation of ligand fluorescence.^83^ A similar profile is observed in the spectra of **1**-Tb, **1**-Dy, and **1**-Tm, which show additional peaks of small intensity almost shadowed by the ligand emission that might be originating from the intraionic transitions of the metal centers. This behavior is somehow expected for **1**-Dy and **1**-Tm based on the results reported by some of us,^35^ which showed that H_2_L does not act as a suitable antenna ligand in the case of Dy^III^ and Tm^III^ derivatives.

However, considering the intense metal-centered emission displayed by the [Zn(μ-L)(μ-OAc)Tb(NO_3_)_2_] counterpart, an efficient quenching mechanism must be operative in **1**-Tb. According to Yamase’s work on the luminesce of lanthanopolyoxotungstates,^22^ low quantum yields are usually observed for Tb^III^ derivatives owing to radiationless deactivation through cross-relaxation processes and Tb^IV^–W^V^ charge-transfer states. This effect can occur by hopping of d^1^ electrons to Ln^III^ ions as a result of fπ–pπ–dπ orbital mixing, but it is found to be favored only when bond angles are higher than 150°; that is, when LnO_*x*_ polyhedra share corners with WO_6_ octahedra. For comparison, this mixing is much less efficient when edge-sharing takes place and Ln–O–W angles are close to 100°. In the case of **1**-Ln complexes, each {LnO_8_} polyhedron shares corners with two WO_6_ units that belong to two different {W_3_O_13_} trimers and displays Ln–O–W angles in the 153–159° range. On the contrary, the other two O_POM_ oxygen atoms in the coordination environment of the 4f metal are those linked to two W centers from the defective trimer. The out-of-pocket coordination mode of the Ln ion precludes its full incorporation to the mixed {LnW_2_O_13_} trimer (*d*_Ln–Oc_ > 3.2 Å, where O_c_ is a central O atom from the Keggin skeleton), and hence, angles in the 128 to 137° range are found, which lie between the two mentioned corner- or edge-sharing modes (Figure S32 and Table S9). All in all, these effects appear to be relevant enough to completely quench the metal-centered emission in **1**-Tb but insufficient in the case of **1**-Eu and **1**-Sm (although their absolute quantum yields at room temperature have been found to be as low as 0.36% and <0.01%, respectively). More specifically, the color of the emission originating from **1**-Eu is similar to that of the [Zn(μ-L)(μ-OAc)Eu(NO_3_)_2_] dimer,^35^ whereas a significant difference can be found in CIE 1931 chromaticity diagrams of the two Sm analogues (Figure S33). The fact that **1**-Sm emits more reddish light in contrast to the pale orange of [Zn(μ-L)(μ-OAc)Sm(NO_3_)_2_] is reflected in their emission spectra, in such a way that relative intensities of ^4^G_5/2_ → ^6^H_7/2_ and ^4^G_5/2_ → ^6^H_9/2_ transitions are almost equal for the nine-coordinated Sm centers of the latter complex.

**Figure 10:**
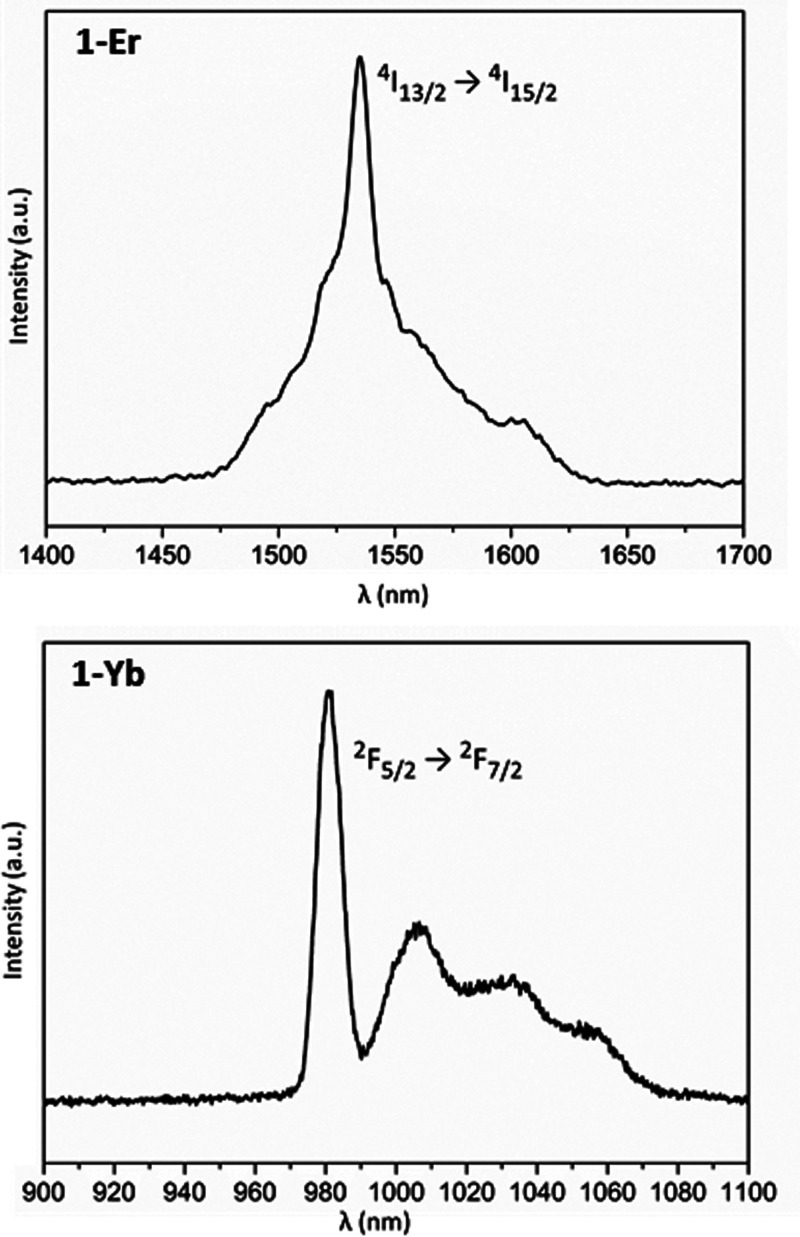
Solid state photoluminescence spectra for **1**-Er (top) and **1**-Yb (bottom) recorded at 10 K upon excitation at 325 nm.

With regard to the emission in the NIR region, this is of high interest, especially in the field of optical communications.^21^ Spectra were only acquired at 20 K upon excitation with 325 nm HeCd continuous laser, because much lower intensity is expected in comparison to those which emit in the visible region. Compound **1**-Er exhibits a broad band in the 1475–1625 nm range with maxima at 1525 nm, which can be ascribed to the ^4^I_13/2_ → ^4^I_15/2_ transition. The **1**-Yb shows a quadruplet in the 970–1060 nm region which is assigned to the ^2^F_5/2_ → ^2^F_7/2_ transition and fits well with the expected crystal field splitting of the ground state (Kramer’s doublets) for this derivative (Figure 10). Previous seminal work revealed that excitation mechanisms different from the antenna effect are possible in the case of Yb^III^ complexes, because there is a large energy gap between the triplet state of the ligand and the ^2^F_5/2_ excited level of the metal.^84^ The related [Zn(μ-L)(μ-OAc)Yb(NO_3_)_2_] dimer showed similar behavior, but no emission in the NIR region was observed for the Er^III^ derivative.^35^ In contrast, the **1**-Ho complex does not exhibit emissive properties even at 20 K. It is worth highlighting that **1**-Er and **1**-Yb represent two of the scarce examples of NIR emitting POM-based systems.^85^

### Solution Stability

**Figure 11:**
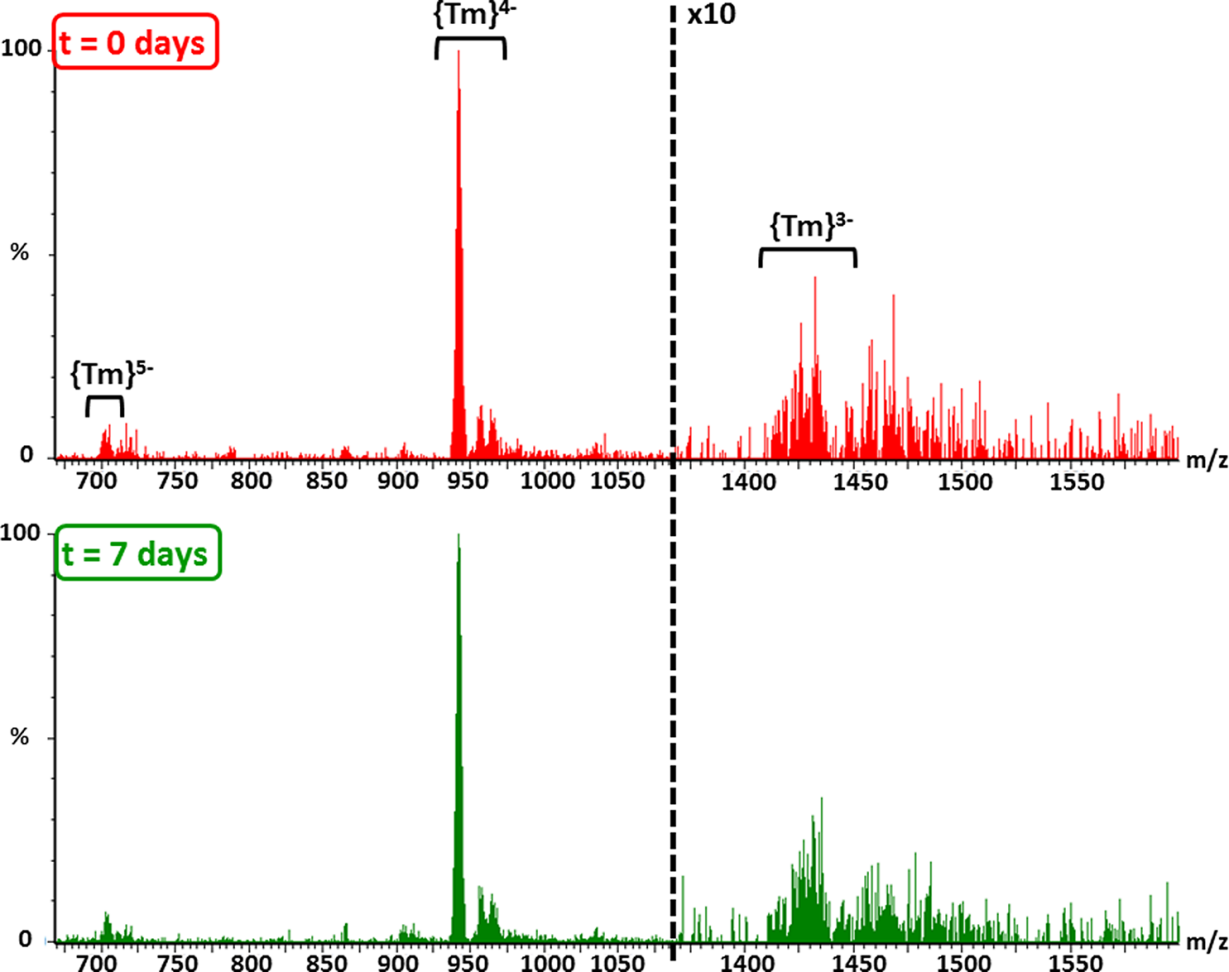
ESI-MS spectra of **1**-Tm in H_2_O/MeCN (1:1) mixtures. Top, freshly prepared solution; bottom, after 1 week. The intensity of the *m*/*z* > 1300 region is increased (×10) for its better visualization.

In order to transfer the interesting properties displayed by the title compounds **1**-Ln to a bulk functional material, it is important to determine whether the hybrid assembly maintains its integrity in solution prior to its immobilization in a solid surface/matrix. Thus, the stability of our {Ln} molecular POM in water was investigated by electrospray ionization mass spectrometry (ESI-MS) experiments carried out for the **1**-Tm derivative. Figure 11 depicts the spectra of a solution in H_2_O/MeCN (1:1) recorded in negative ion mode at low cone voltage. Three groups of signals spanning from *m*/*z* 670 to 1500 indicate the presence of the intact {Tm} anion in the freshly prepared aqueous solution. The *m*/*z* spacing between the group of signals and isotopic pattern inspection evidence that they match well with anionic species of similar composition, but −5 (*m*/*z* 704.8, {Tm}^5–^), −4 (*m*/*z* 942.7, {Tm}^4–^), and −3 (*m*/*z* 1434.6, {Tm}^3–^) charge states. Each group of signals does not correspond to a single specific species but to series of general formula [Tm(H_2_L)(SiW_11_O_39_)+ *m*K^+^ + *n*H^+^ + *x*H_2_O]^(5–*m*−*n*)–^, because the high negative charge of the anion allows different extents of protonation and a variable number of associated counterion/solvent molecules to be present. This is a common fact found in the ESI-MS spectra of POM species.^64^ Nevertheless, the experimental isotopic pattern of the most abundant group centered at *m*/*z* 942.7 compares well with that simulated for the [Tm(H_2_L)(SiW_11_O_39_) + K]^4–^ ion, which confirms our previous assignment (Figure S34). The spectrum remains virtually unchanged for 1 week, and thus, it demonstrates that hybrid molecular assemblies in **1**-Tm are stable in water solution. These results can be easily extended to the remaining **1**-Ln counterparts, as indicated by the virtually identical results obtained for the **1**-Tb derivative (Figure S35).

## Conclusions

This work nicely exemplifies the potential of lanthanide ion/lacunary POM/aromatic multidentate organic ligand synthetic systems as dynamic libraries of building blocks with the ability to construct molecular materials with interesting optical and magnetic properties. In this case, the reaction of mid-to-late lanthanide(III) ions with monolacunary α-Keggin-type polyanions and a compartmental organic ligand (H_2_L) leads to a series of 10 isostructural hybrids with the general formula K_5_[Ln(α-SiW_11_O_39_)(H_2_L)]·14H_2_O (**1**-Ln, Ln = Sm to Lu), which constitute some of the very scarce examples of mononuclear lanthanide complexes containing simultaneously organic and inorganic ligands. Unlike all of the metal complexes previously reported for this ligand, the empty N_2_O_2_ coordination site allows the folding of the organic ligand, in such a way that weak Br···Br and π–π interactions are established between adjacent molecular units and result in supramolecular chairlike assemblies of six hybrid anions.

Compounds **1**-Gd and **1**-Yb display slow relaxation of the magnetization below ∼6 K, which mechanistically takes place through a combination of different relaxation processes. Furthermore, the metal-centered luminescence is efficiently sensitized by the organic antenna ligand for **1**-Sm and **1**-Eu in the visible region, as well as **1**-Er and **1**-Yb in the NIR. In contrast, the quenching of the emission for **1**-Tb has been attributed to the out-of-pocket coordination mode of the lanthanide center within the POM fragment. To our knowledge, **1**-Yb represents the first lanthanide-containing POM-based system to exhibit simultaneous slow magnetic relaxation and NIR emission. Finally, the stability of hybrid POMs in aqueous solutions has been addressed by ESI-MS experiments.

Besides the multiple combinations that could arise from the accurate selection of both organic and inorganic components, the use of compartmental ligands allows the preparation of heterometallic 3d–4f complexes. In the near future, we plan to make use of the available N_2_O_2_ coordination site to accommodate a transition metal ion, which might enhance the anisotropy of the system and result in improved magnetic properties.

## Supporting Information Available

FT-IR spectra, PXRD patterns, TGA/DTA curves, UV/vis absorption spectra, complementary magnetisms and luminescence data, structural figures, and tables (PDF)

## Accession Codes

CCDC 2115979–2115989 contain the supplementary crystallographic data for this paper. These data can be obtained free of charge via www.ccdc.cam.ac.uk/data_request/cif, or by emailing data_request@ccdc.cam.ac.uk, or by contacting The Cambridge Crystallographic Data Centre, 12 Union Road, Cambridge CB2 1EZ, UK; fax: +44 1223 336033.
