# Supplementary material for: Slow Magnetic Relaxation and Luminescent Properties of Mononuclear Lanthanide-Substituted Keggin-Type Polyoxotungstates with Compartmental Organic Ligands
Source: Inorg Chem. 2022 Jan 27;61(5):2428–43. doi: 10.1021/acs.inorgchem.1c03214 (PMC8826278; doi:10.1021/acs.inorgchem.1c03214)
Supplement: Supplementary file 1 — ic1c03214_si_001.pdf [file ic1c03214_si_001.pdf]

Supporting information

Slow magnetic relaxation and luminescent properties of mononuclear lanthanide-substituted Keggin-type polyoxotungstates with compartmental organic ligands

Estibaliz Ruiz-Bilbao,[‡] Markel Pardo-Almanza,[‡,⊥] Itziar Oyarzabal,[§,ǁ] Beñat Artetxe,[*,‡] Leire San Felices,[†] José A. García,[§,δ] José Manuel Seco,[└] Enrique Colacio,[$] Luis Lezama,[*,‡] Juan M. Gutiérrez-Zorrilla[‡,§]

[‡] Departamento de Química Inorgánica, Facultad de Ciencia y Tecnología. Universidad del País Vasco UPV/EHU, P.O. Box 644, 48080 Bilbao, Spain.

[⊥] Quantum Materials Science Unit, Okinawa Institute of Science and Technology Graduate University, 1919-1 Tancha, Onna, Okinawa 904-0495, Japan.

[§] BCMaterials, Basque Center for Materials, Applications and Nanostructures, UPV/EHU Science Park, 48940 Leioa, Spain.

[ǁ] IKERBASQUE, Basque Foundation for Science, Bilbao, 48009, Spain.

[†] Servicios Generales de Investigación SGIker, Facultad de Ciencia y Tecnología. Universidad del País Vasco UPV/EHU, P.O. Box 644, 48080 Bilbao, Spain.

[δ] Departamento de Física Aplicada II, Facultad de Ciencia y Tecnología. Universidad del País Vasco UPV/EHU, P.O. Box 644, 48080 Bilbao, Spain.

[└] Departamento de Química Aplicada, Facultad de Química, Universidad del País Vasco UPV/EHU, 20018 San Sebastián, Spain.

[$] Departamento de Química Inorgánica, Facultad de Ciencias, Universidad de Granada, 18071 Granada, Spain.

* E-mail: benat.artetxe@ehu.eus (B.A.), luis.lezama@ehu.eus (L.L.)

TABLE OF CONTENTS...

Figures

Figure S1. Scheme of the H_2L ligand. 4

Figure S2. TGA curves of compounds **1-Ln** (Ln = Sm to Lu). 4

Figure S3. Identification of the final residue from the thermal decomposition of **1-Dy** by PXRD analyses. 5

Figure S4. FT-IR spectra of compound **2-Dy** in comparison to the starting materials (H_2L ligand, and the $K_8[\alpha\text{-}SiW_{11}O_{39}]$ POM precursor). 5

Figure S5. FT-IR spectra of **1-Dy** and the yellow precipitate formed in its synthesis compared with that of **2-Dy**. 6

Figure S6. Experimental powder X-ray diffraction pattern of the precipitate formed in the reaction of **1-Dy** (left). Pattern matching between the experimental pattern of **1-Dy** and cell parameters determined by single-crystal X-ray diffraction analyses (middle), together with the detail of the most intense diffraction maxima (right). 6

Figure S7. Experimental powder X-ray diffraction patterns of the freshly filtered crystals. 7

Figure S8. FT-IR spectra of **1-Ln** (Ln = Sm to Lu). 8

Figure S9. FT-IR spectrum of **1-Dy** compared to those recorded for crystals obtained in analogous reactions but using early lanthanide ions (La to Nd). The latter has been identified as Peacock-Weakley type $[Ln^{III}(SiW_{11}O_{39})_2]^{13-}$ sandwich POMs. 9

Figure S10. ORTEP view of the hybrid molecular $[Sm(H_2L)(\alpha\text{-}SiW_{11}O_{39})]^{5-}$ **{Sm}** anion in **1-Sm** showing 50% probability displacement ellipsoids, together with atom labelling for heavy atoms. 9

Figure S11. Plot of the average intramolecular Cg···Cg distances vs. dihedral angles between aromatic rings for all the crystallographically independent H_2L/L^{2-} fragments deposited in the CSD database. Fragments from heterometallic dinuclear (Ln^{III}/M^{II}) complexes are depicted in blue (M = Zn, Cu) and pink (M = Co, Ni), whereas those belonging to sandwich-type species with more than one H_2L ligand are colored in orange. Data points from compounds **1-Ln** reported in this work are indicated in green. 10

Figure S12. Illustration of the parallel arrangement of one of the aromatic groups with respect to the ideal mirror plane of the Keggin anion in molecular **{Ln}** units represented by a dashed orange line. Intramolecular hydrogen bonds are depicted as blue dashed lines. 10

Figure S13. Variation of average lanthanide-oxygen bond lengths (Å) with the atomic number of the 4f metal center in compounds **1-Ln.** *Abbreviations*: O_a: O atoms from the aldehyde group of the H_2L ligand; O_p: O atoms from the phenoxy group of the H_2L ligand; O_{POM}: O atoms delimiting the vacant site of the lacunary Keggin-type POM. 11

Figure S14. Representation of the chair-like conformation displayed by the **{Ln}** molecular hybrid species in **1-Ln** represented by their Ln atoms and average Ln···Ln distances (Å). 11

Figure S15. Temperature dependence of the magnetic susceptibility (χ_M) at 1000 Oe for **1-Sm** and **1-Eu**. Black solid lines represent the best fit to the magnetic data as described in the main text. 11

Figure S16. Field dependent magnetization curves at different temperatures for **1-Ln**. Black solid lines represent the best fit to the magnetic data as described in the main text. The rest of the lines are a guide to the eye. 12

Figure S16 (c*ontinuation*). Field dependent magnetization curves at different temperatures for **1-Ln**. Black solid lines represent the best fit to the magnetic data as described in the main text. The rest of the lines are a guide to the eye. 13

Figure S17. Q-band EPR spectra collected for **1-Gd** at room temperature. 13

Figure S18. Temperature dependence of the magnetic susceptibility (χ_M) at 1000 Oe for **1-Tm**. The black solid line represents the best fit to the magnetic data as described in the main text. 14

Figure S19. Energy level diagram for **1-Tm**. 14

Figure S20. Temperature dependence of the in-phase (χ_M', top) and out-of-phase (χ_M", bottom) components of the *ac* susceptibility for **1-Dy** under an external field of 1000 Oe. 15

Figure S21. Variable temperature frequency dependence of the χ_M' (top) and χ_M" (bottom) signals under 1000 Oe applied field for **1-Gd**. Solid lines represent the best fitting of experimental data to the Debye model. 16

Figure S22. Variable temperature frequency dependence of the χ_M' (top) and χ_M" (bottom) signals under 1000 Oe applied field for **1-Yb**. Solid lines represent the best fitting of experimental data to the Debye model. 17

Figure S23. Cole-Cole plots for **1-Gd** (top) and **1-Yb** (bottom). 18

Figure S24. Temperature dependence of χ_M' (top) and χ_M" (bottom) components of the *ac* susceptibility for **1-Gd** under external fields of 2000 Oe (left) and 3000 Oe (right). 19

Figure S25. Relaxation times obtained under different applied fields for **1-Gd** with the best fitting of the data to eq. 4 (Eq. 4. $\tau^{-1} = CT^n + \tau_{QTM}^{-1}$). 20

Figure S26. UV-Vis diffuse reflectance absorption spectra of H_2L ligand (left), $K_8[\alpha\text{-}SiW_{11}O_{39})]\cdot 13H_2O$ POM precursor (middle) and **1-Gd** (right). 20

Figure S27. Left: Photographs of crystals of **1-Sm** (top) and **1-Eu** (bottom) under irradiation of UV light. Right: photographs of bulk crystalline samples before and after irradiation with UV light. 21

Figure S28. Low temperature (10K) solid state excitation spectra for **1-Sm** (left) and **1-Eu** (right) recorded for their most intense emission bands (600 and 614 nm, respectively). 21

Figure S29. Luminescence decay curves for **1-Sm** at different temperatures upon excitation at 375 nm. 22

Figure S30. Luminescence decay curves for **1-Eu** at different temperatures upon excitation at 375 nm. 22

Figure S31. Solid state photoluminescence spectra of **1-Gd**, **1-Tb**, **1-Dy** and **1-Tm** recorded at 10K and upon excitation at 325 nm. 23

Figure S32. Scheme of coordination spheres of the emitting Ln centers in **1-Ln.** Labels O_c, O_i , and O_{Si} denote O atoms involved in corner sharing, those in intermediate situations between corner and edge sharing and central O atoms, respectively. 23

Figure S33. CIE 1931 x,y chromaticity coordinates as a function of the emission wavelengths for compounds **1-Ln** (Ln = Sm, Eu) compared to previously reported [Zn(μ-L)(μ-OAc)Ln$(NO_3)_2$]·MeCN (Ln = Sm, Eu, Tb) derivatives with the same H_2L ligand (Oyarzabal I. et al. *Dalton Trans.* **2016**, *45*, 9712). 24

Figure S34. Expanded region of the negative ESI mass spectrum for the freshly prepared solution of **1-Tm** which corresponds to the **{Tm}**$^{4-}$ series, compared to that recorded one week later and the simulated isotopic pattern for the [Tm(H_2L)($SiW_{11}O_{39}$) + K]$^{4-}$ species. 25

Figure S35. Negative ESI-MS spectra of **1-Tm** in H_2O/MeCN (1:1) mixture compared to that acquired for **1-Tb** derivative. The intensity of the m/z > 1300 region is increased (x10) for its better visualization. 26

Tables

Table S1. Thermal data for compounds **1-Ln**. 27

Table S2. Donor···acceptor distances (Å) for intramolecular N–H···O type hydrogen bonds in **1-Ln**. 27

Table S3. Selected geometrical parameters for all the structures containing the H_2L ligand and lanthanide ions included in the CSD database: Intramolecular centroid···centroid ($C_g \cdots C_g$) distances (Å) and dihedral angles (°) between aromatic rings. 28

Table S4. Continuous Shape Measurements (CSM) for the eight coordinated lanthanide atoms in compounds **1-Ln**.[a] 29

Table S5. Lanthanide-Oxygen bond lengths (Å) and Ln···Ln distances (Å) in compounds **1-Ln.** 30

Table S5 (*continuation*). Lanthanide-Oxygen bond lengths (Å) and Ln···Ln distances (Å) in compounds **1-Ln**. 31

Table S6. Geometrical parameters (Å, °) of intermolecular π–π interactions in **1-Ln**. 32

Table S7. Br···Br distances (Å) in compounds **1-Ln.** 33

Table S8. Values of *C, n* and τ_{QTM}^{-1} parameters obtained from the best fitting of relaxation times for different applied fields in **1-Gd.** 33

Table S9. Ln–O_{POM}–W bond angles in **1-Ln**. Note the difference between oxygen atoms involved in corner sharing (O_c) and those in intermediate situations between corner and edge sharing (O_i). 34

Figures

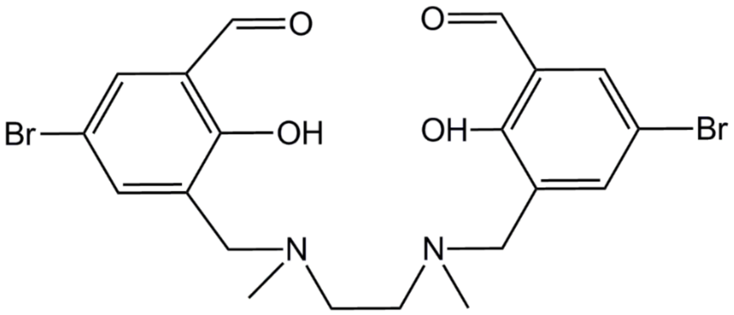

Figure S1. Scheme of the H_2L ligand.

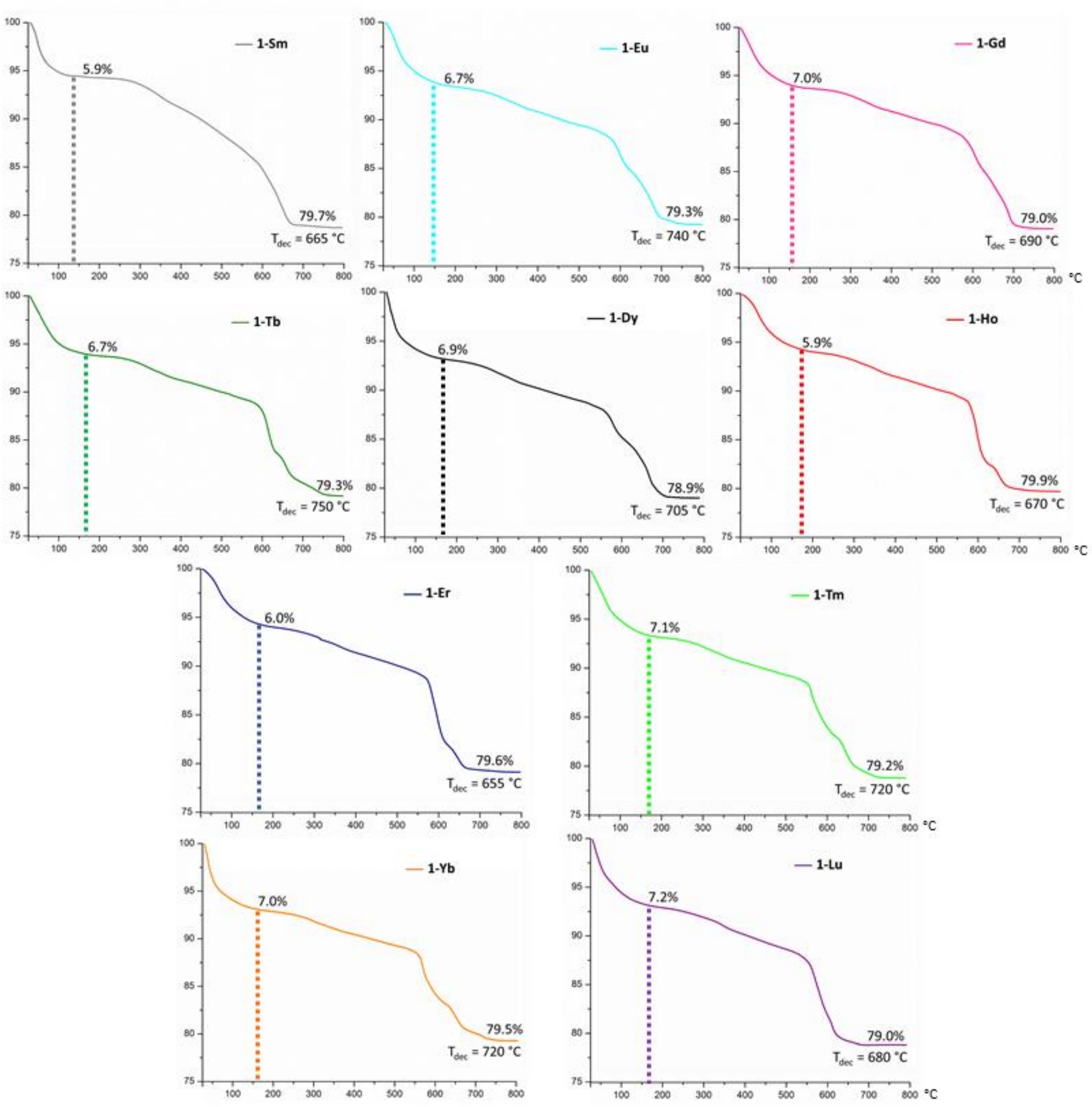

Figure S2. TGA curves of compounds **1-Ln** (Ln = Sm to Lu).

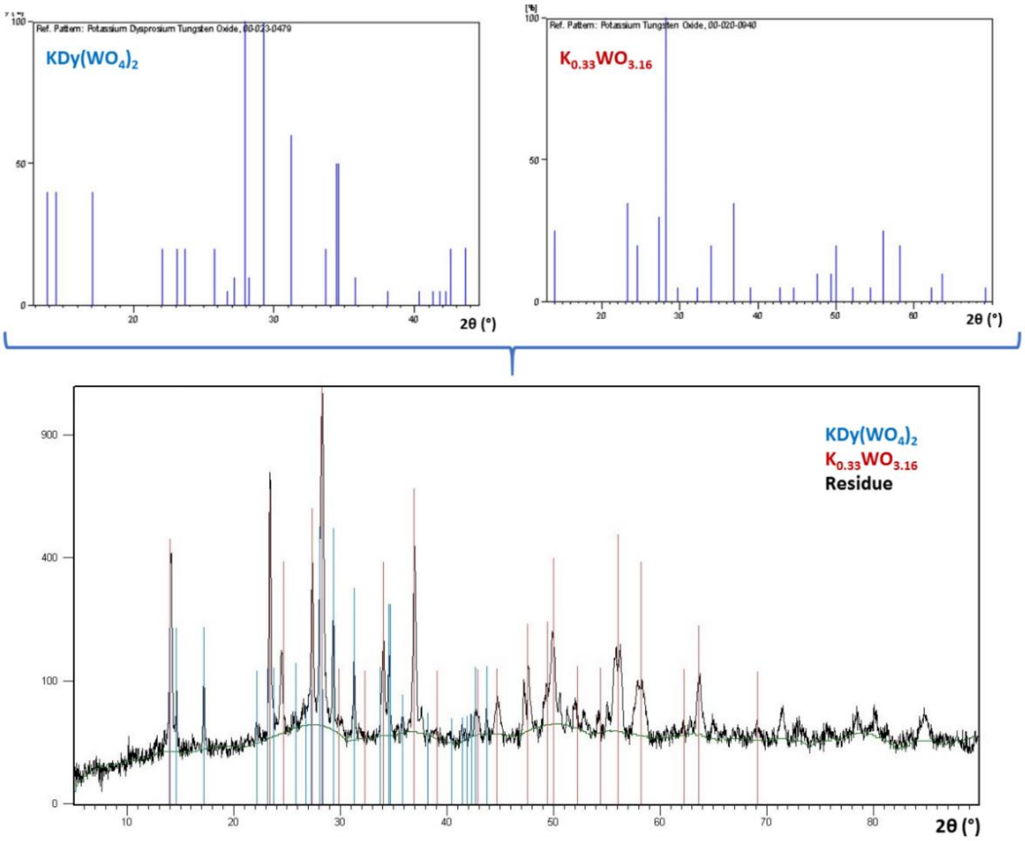

Figure S3. Identification of the final residue from the thermal decomposition of **1-Dy** by PXRD analyses.

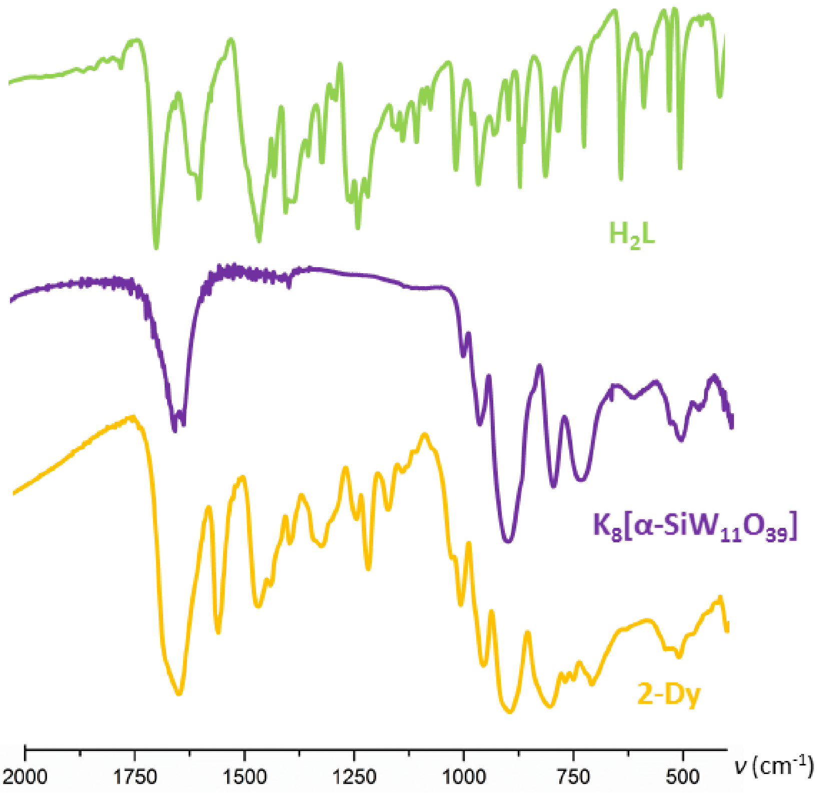

Figure S4. FT-IR spectra of compound **2-Dy** in comparison to the starting materials (H_2L ligand, and the $K_8[\alpha\text{-}SiW_{11}O_{39}]$ POM precursor).

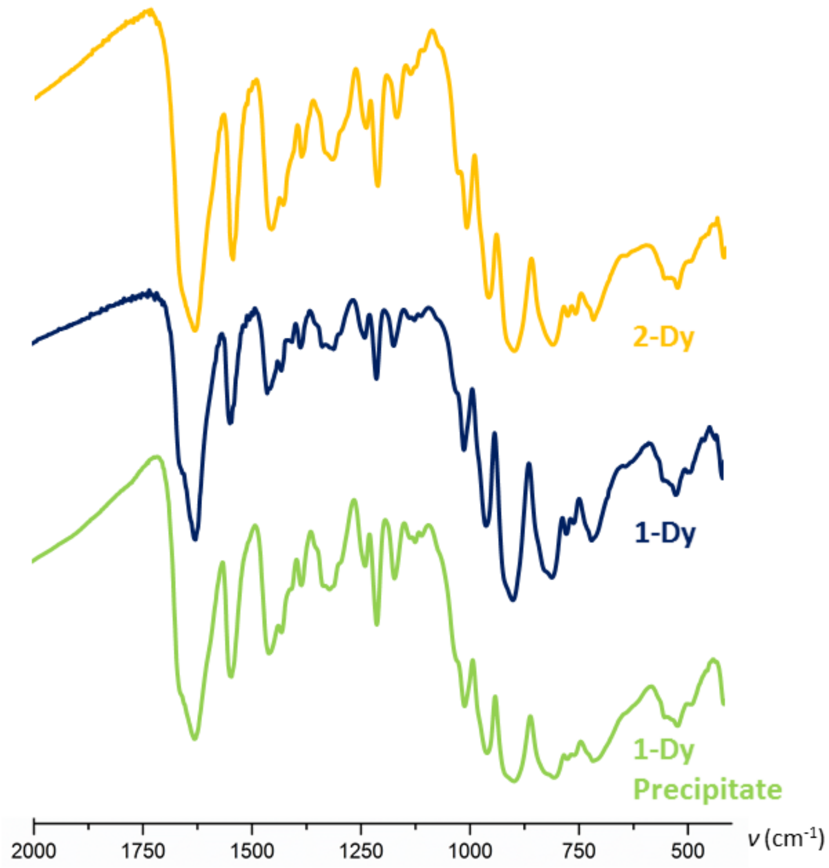

Figure S5. FT-IR spectra of **1-Dy** and the yellow precipitate formed in its synthesis compared with that of **2-Dy**.

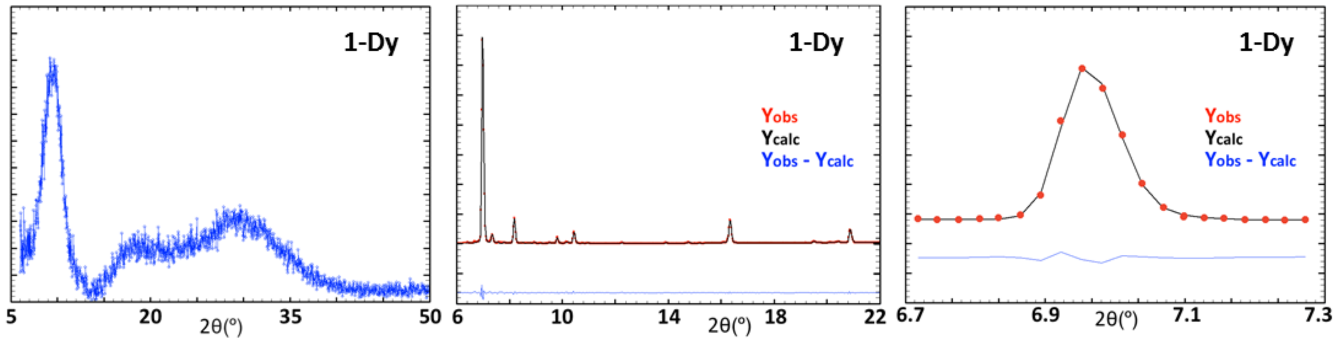

Figure S6. Experimental powder X-ray diffraction pattern of the precipitate formed in the reaction of **1-Dy** (left). Pattern matching between the experimental pattern of **1-Dy** and cell parameters determined by single-crystal X-ray diffraction analyses (middle), together with the detail of the most intense diffraction maxima (right).

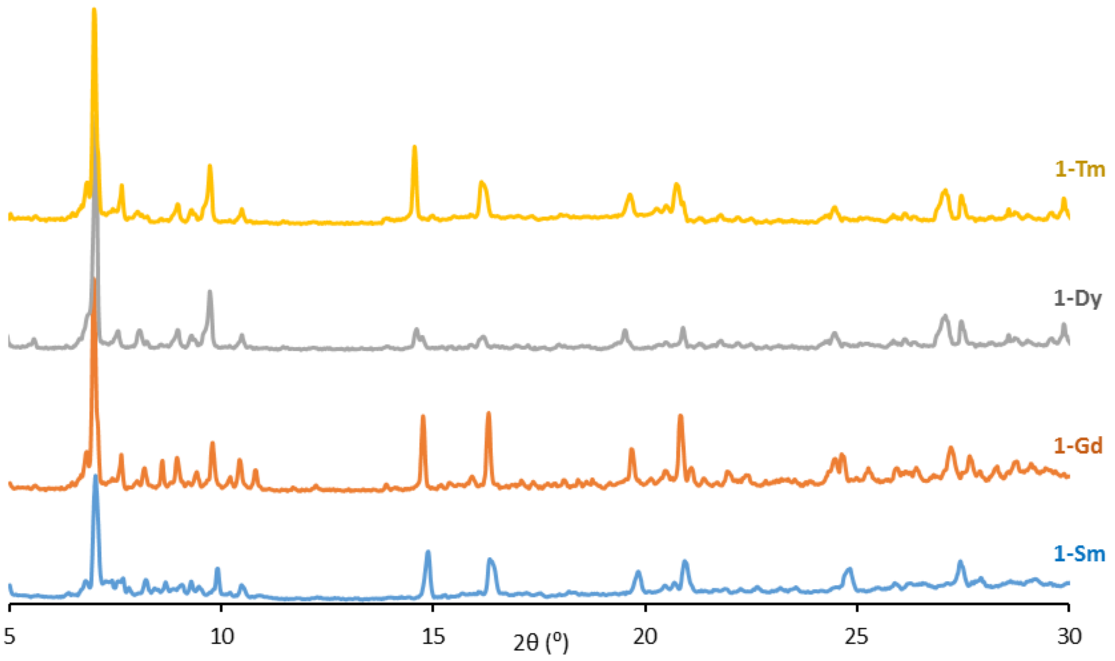

Figure S7. Experimental powder X-ray diffraction patterns of the freshly filtered crystals.

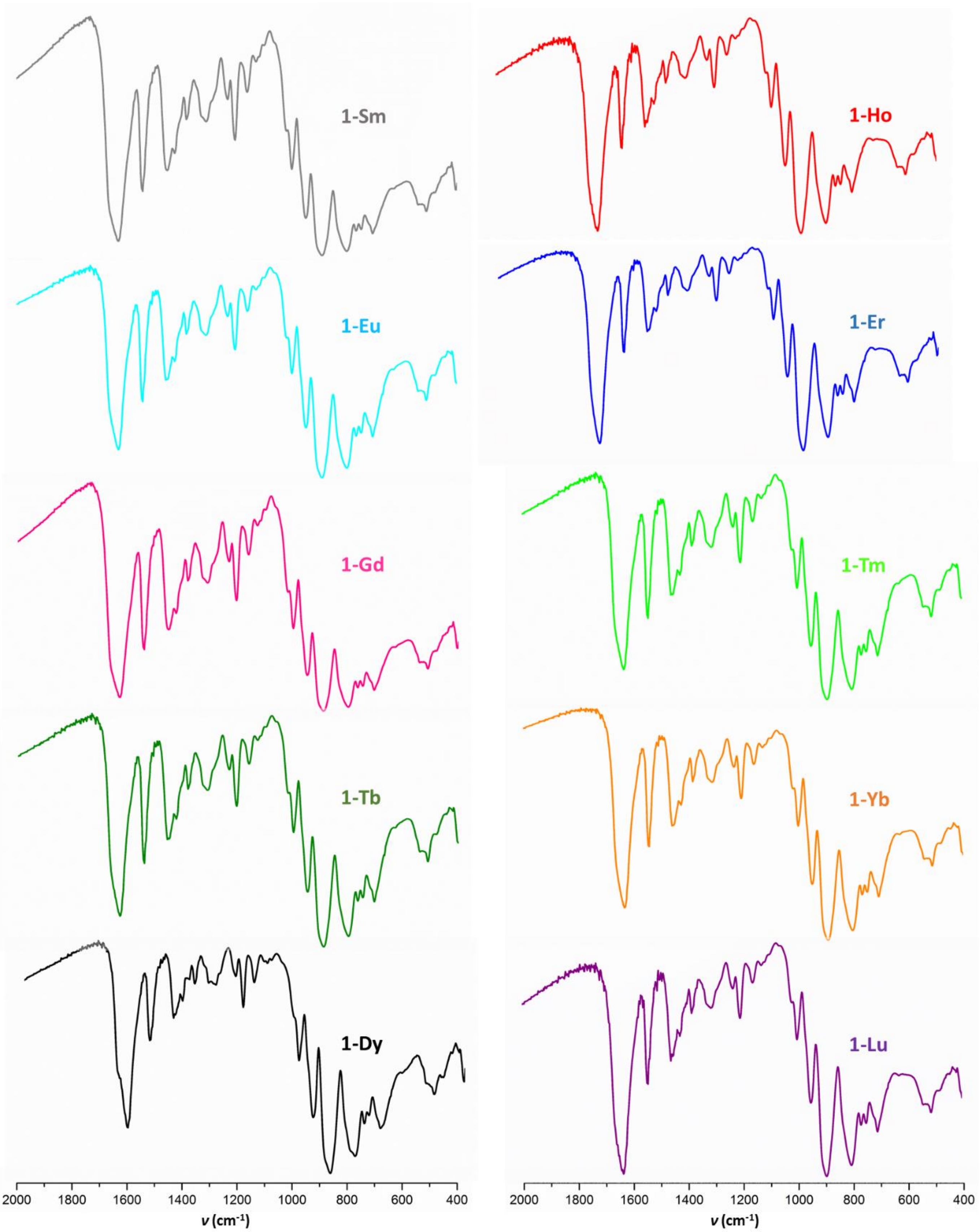

Figure S8. FT-IR spectra of **1-Ln** (Ln = Sm to Lu).

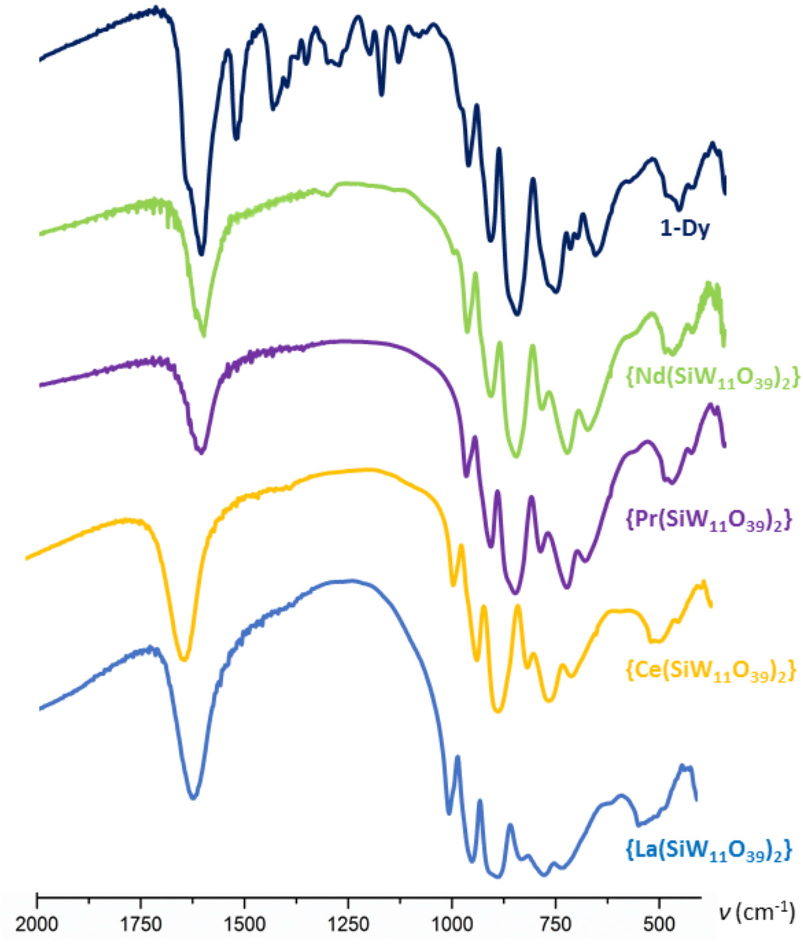

Figure S9. FT-IR spectrum of **1-Dy** compared to those recorded for crystals obtained in analogous reactions but using early lanthanide ions (La to Nd). The latter has been identified as Peacock-Weakley type $[Ln^{III}(SiW_{11}O_{39})_2]^{13-}$ sandwich POMs.

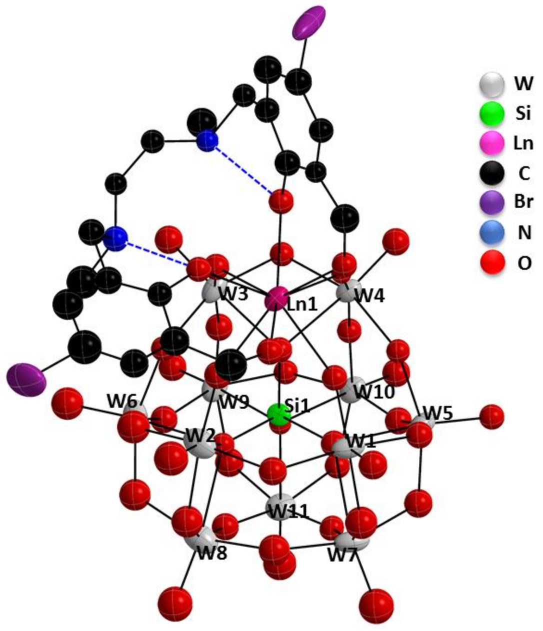

Figure S10. ORTEP view of the hybrid molecular $[Sm(H_2L)(\alpha\text{-}SiW_{11}O_{39})]^{5-}$ **{Sm}** anion in **1-Sm** showing 50% probability displacement ellipsoids, together with atom labelling for heavy atoms.

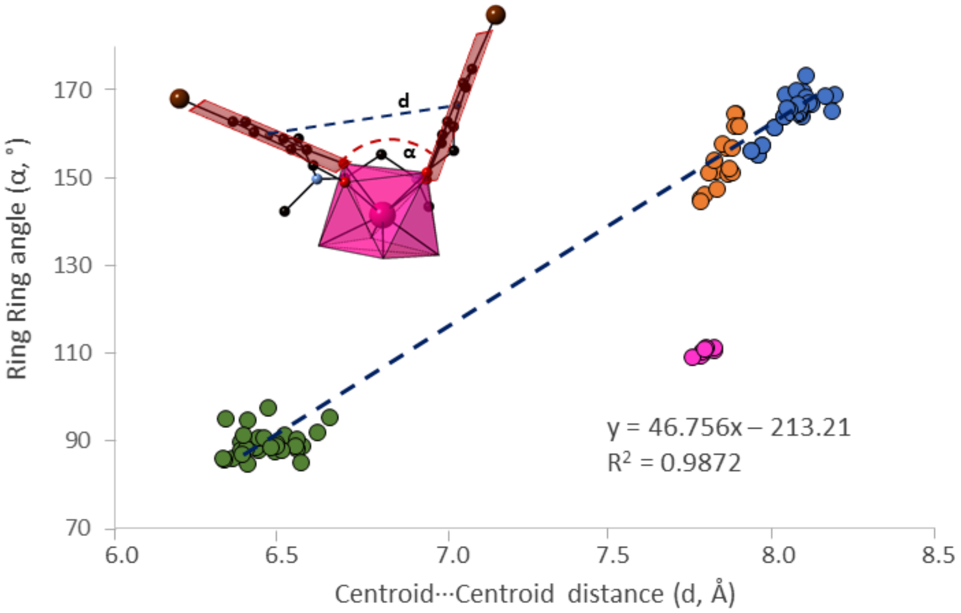

Figure S11. Plot of the average intramolecular Cg···Cg distances vs. dihedral angles between aromatic rings for all the crystallographically independent H_2L/L^{2-} fragments deposited in the CSD database. Fragments from heterometallic dinuclear (Ln^{III}/M^{II}) complexes are depicted in blue (M = Zn, Cu) and pink (M = Co, Ni), whereas those belonging to sandwich-type species with more than one H_2L ligand are colored in orange. Data points from compounds **1-Ln** reported in this work are indicated in green.

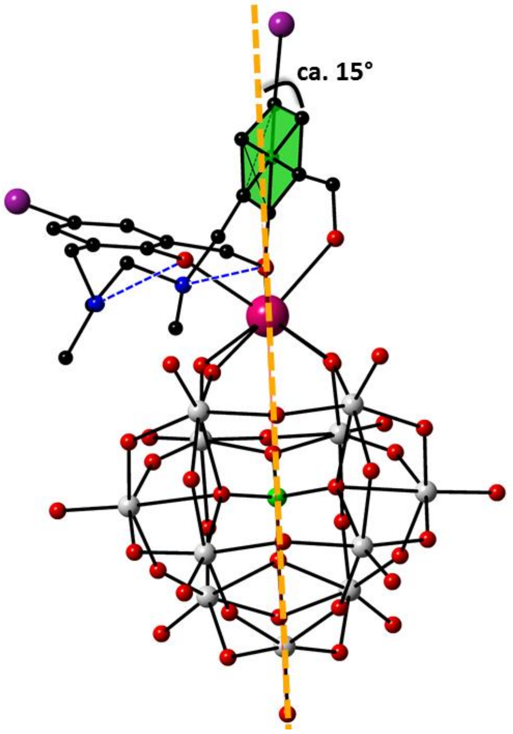

Figure S12. Illustration of the parallel arrangement of one of the aromatic groups with respect to the ideal mirror plane of the Keggin anion in molecular **{Ln}** units represented by a dashed orange line. Intramolecular hydrogen bonds are depicted as blue dashed lines.

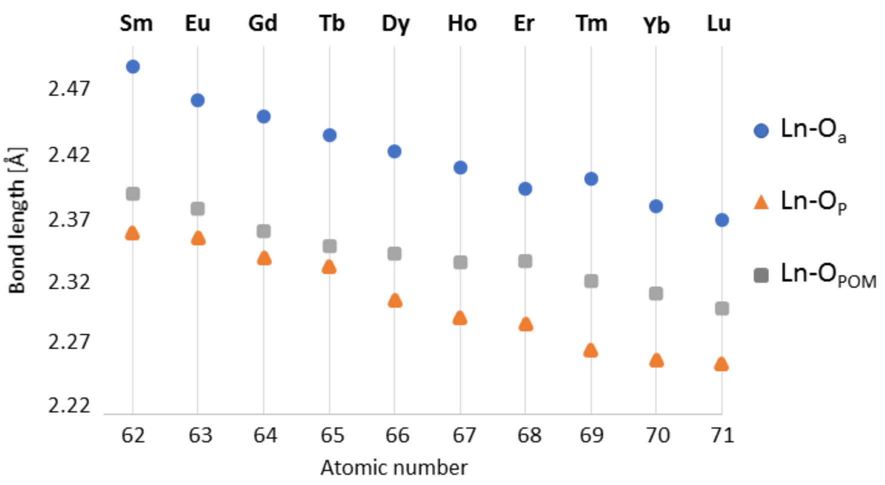

Figure S13. Variation of average lanthanide-oxygen bond lengths (Å) with the atomic number of the 4f metal center in compounds **1-Ln.** *Abbreviations*: O_a: O atoms from the aldehyde group of the H_2L ligand; O_p: O atoms from the phenoxy group of the H_2L ligand; O_{POM}: O atoms delimiting the vacant site of the lacunary Keggin-type POM.

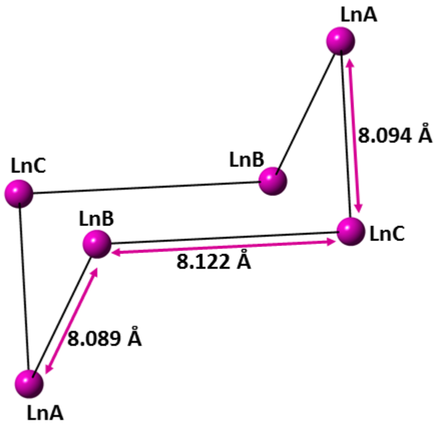

Figure S14. Representation of the chair-like conformation displayed by the **{Ln}** molecular hybrid species in **1-Ln** represented by their Ln atoms and average Ln···Ln distances (Å).

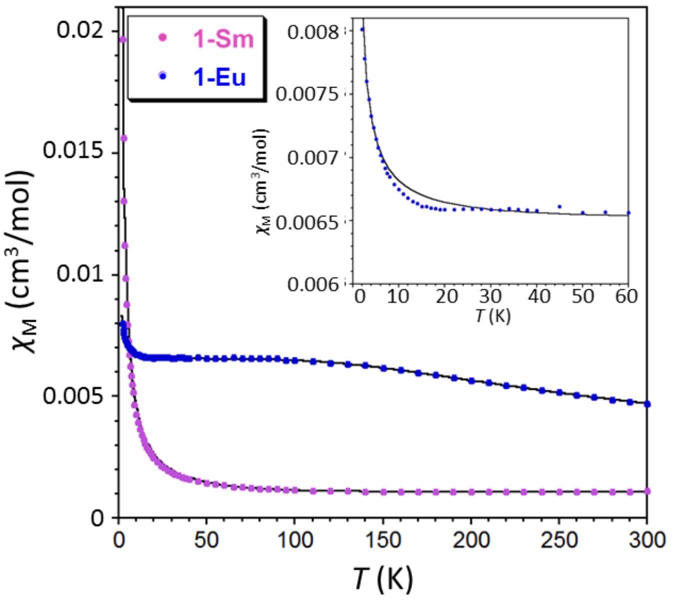

Figure S15. Temperature dependence of the magnetic susceptibility (χ_M) at 1000 Oe for **1-Sm** and **1-Eu**. Black solid lines represent the best fit to the magnetic data as described in the main text.

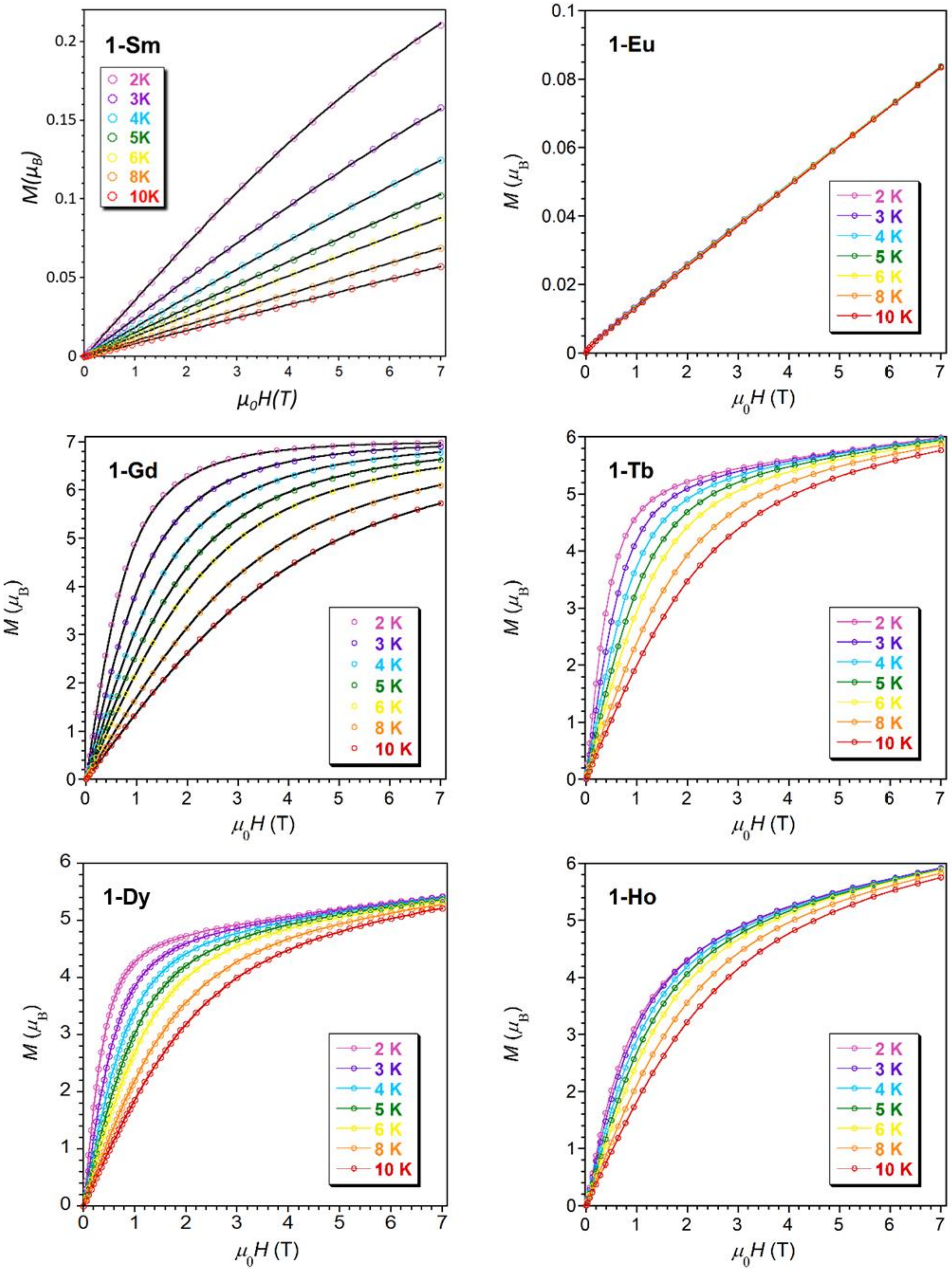

Figure S16. Field dependent magnetization curves at different temperatures for **1-Ln**. Black solid lines represent the best fit to the magnetic data as described in the main text. The rest of the lines are a guide to the eye.

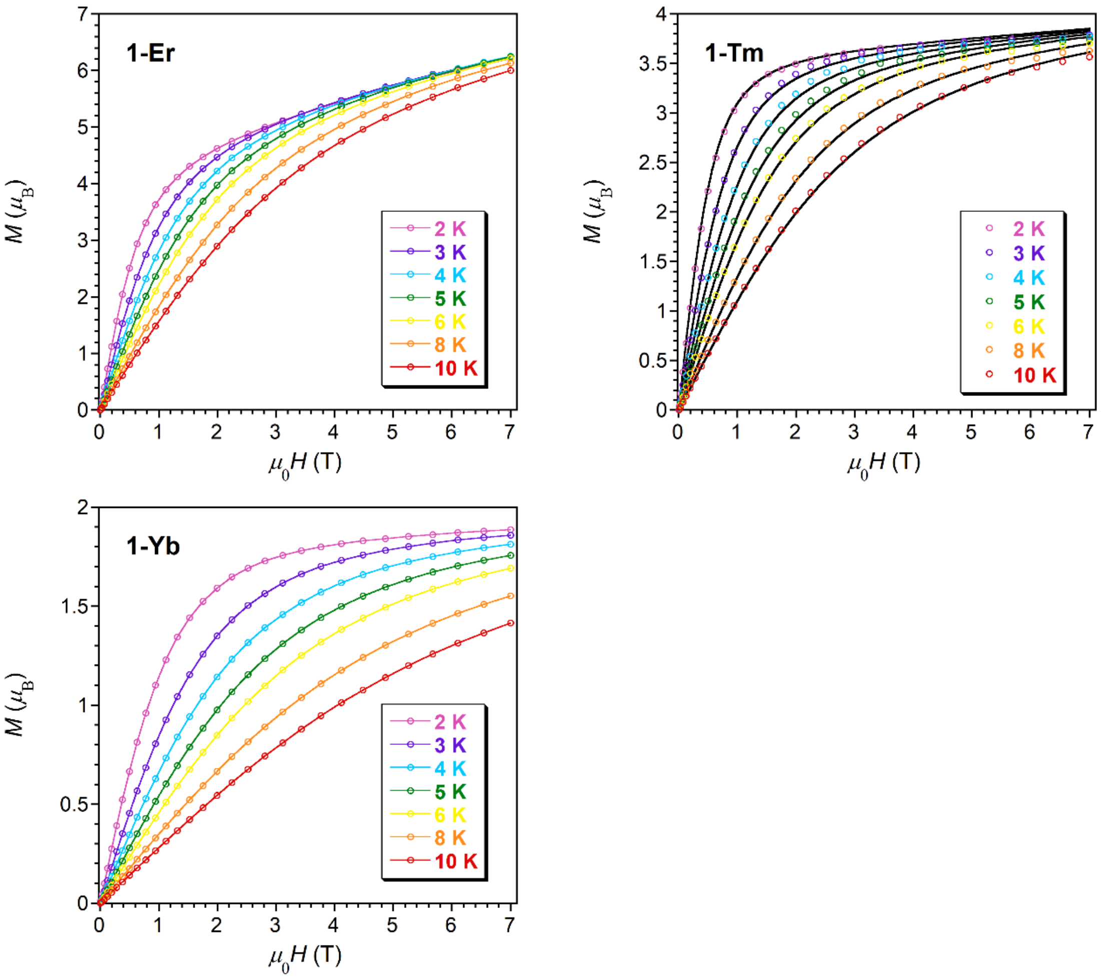

Figure S16 (*continuation*). Field dependent magnetization curves at different temperatures for **1-Ln**. Black solid lines represent the best fit to the magnetic data as described in the main text. The rest of the lines are a guide to the eye.

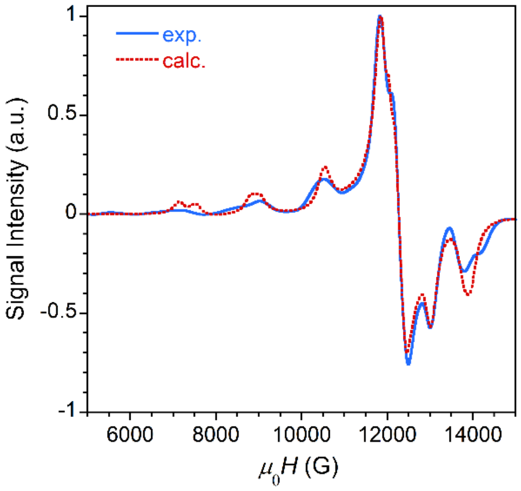

Figure S17. Q-band EPR spectra collected for **1-Gd** at room temperature.

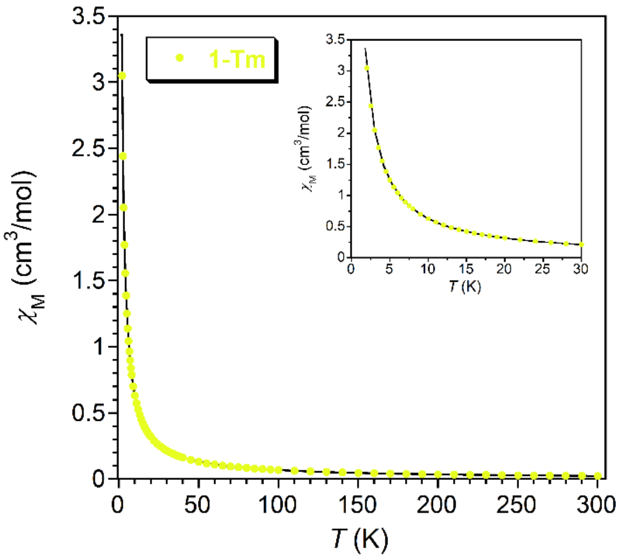

Figure S18. Temperature dependence of the magnetic susceptibility (χ_M) at 1000 Oe for **1-Tm**. The black solid line represents the best fit to the magnetic data as described in the main text.

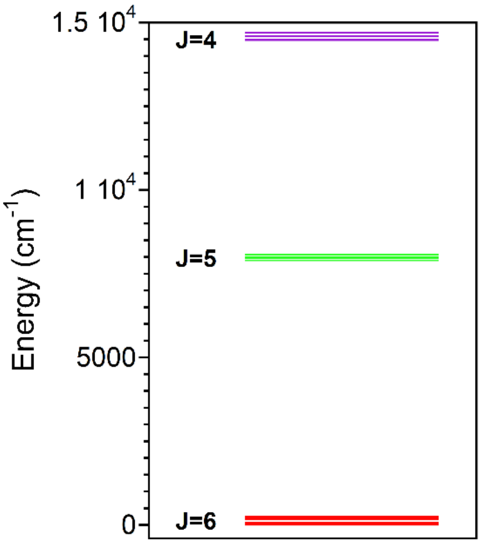

Figure S19. Energy level diagram for **1-Tm**.

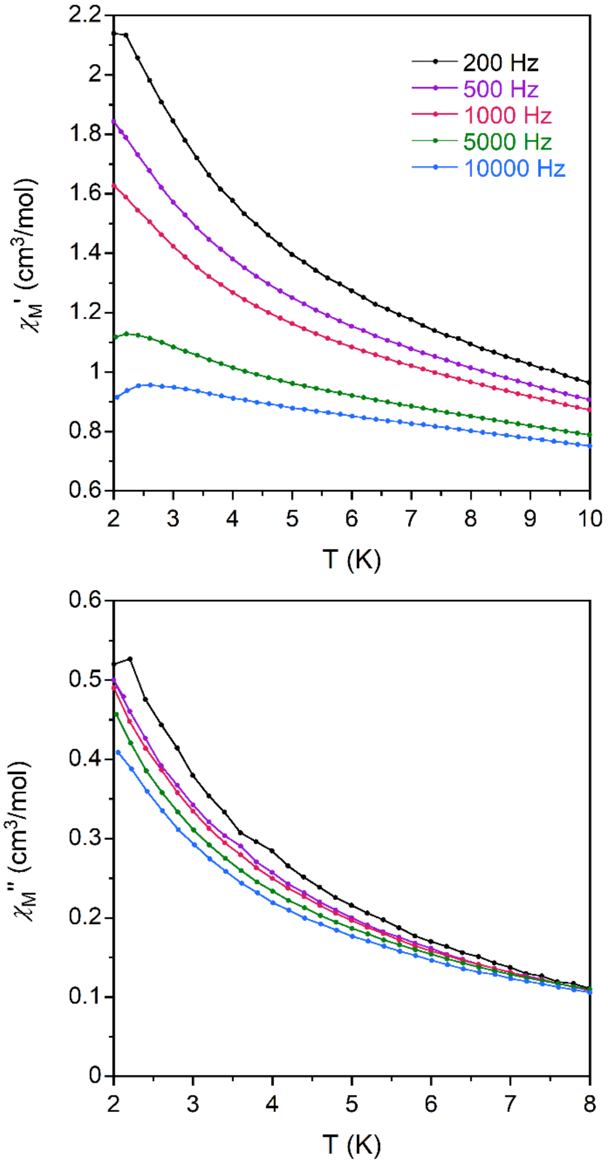

Figure S20. Temperature dependence of the in-phase (χ_M', top) and out-of-phase (χ_M'', bottom) components of the *ac* susceptibility for **1-Dy** under an external field of 1000 Oe.

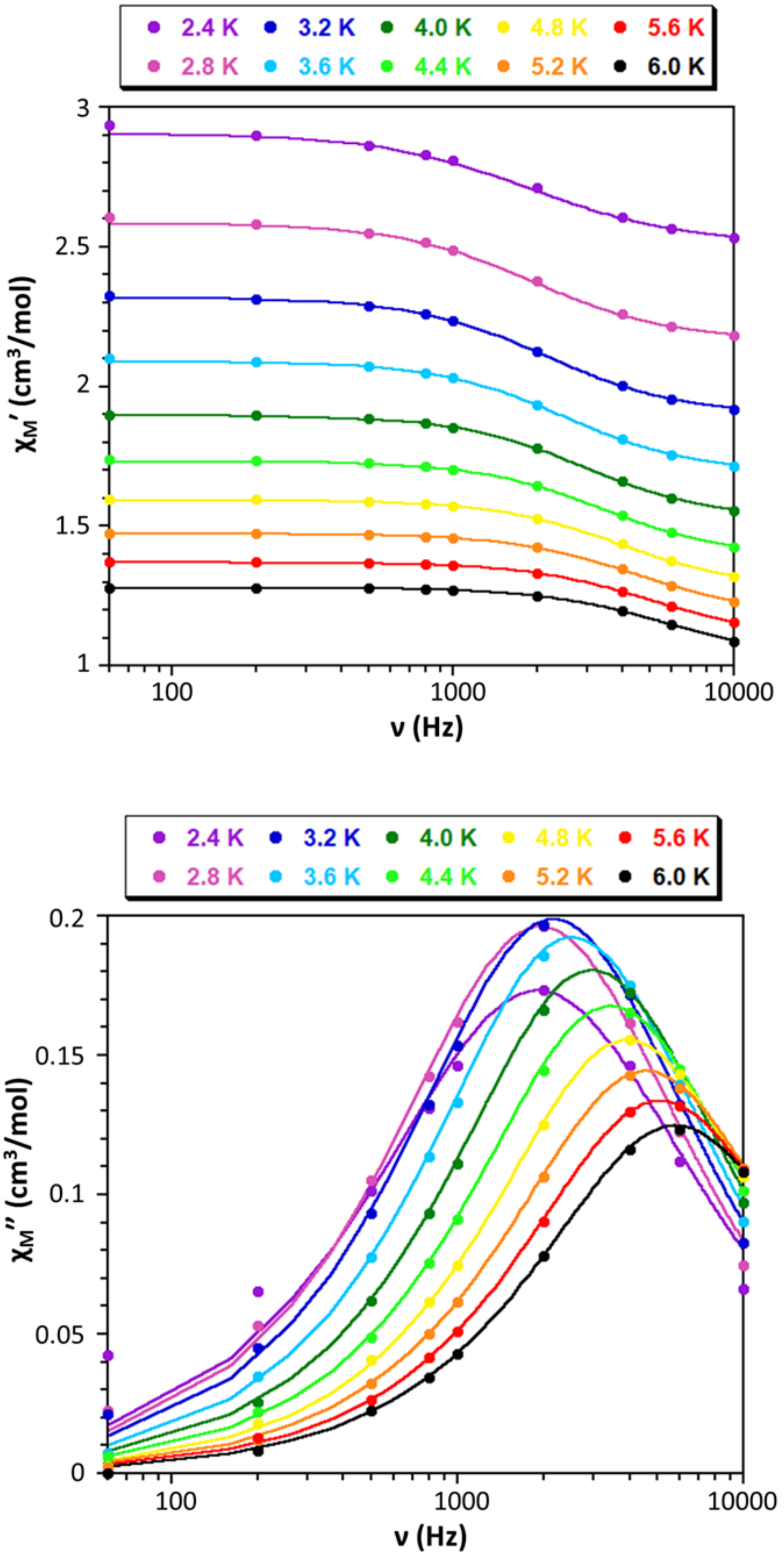

Figure S21. Variable temperature frequency dependence of the χ_M' (top) and χ_M'' (bottom) signals under 1000 Oe applied field for **1-Gd**. Solid lines represent the best fitting of experimental data to the Debye model.

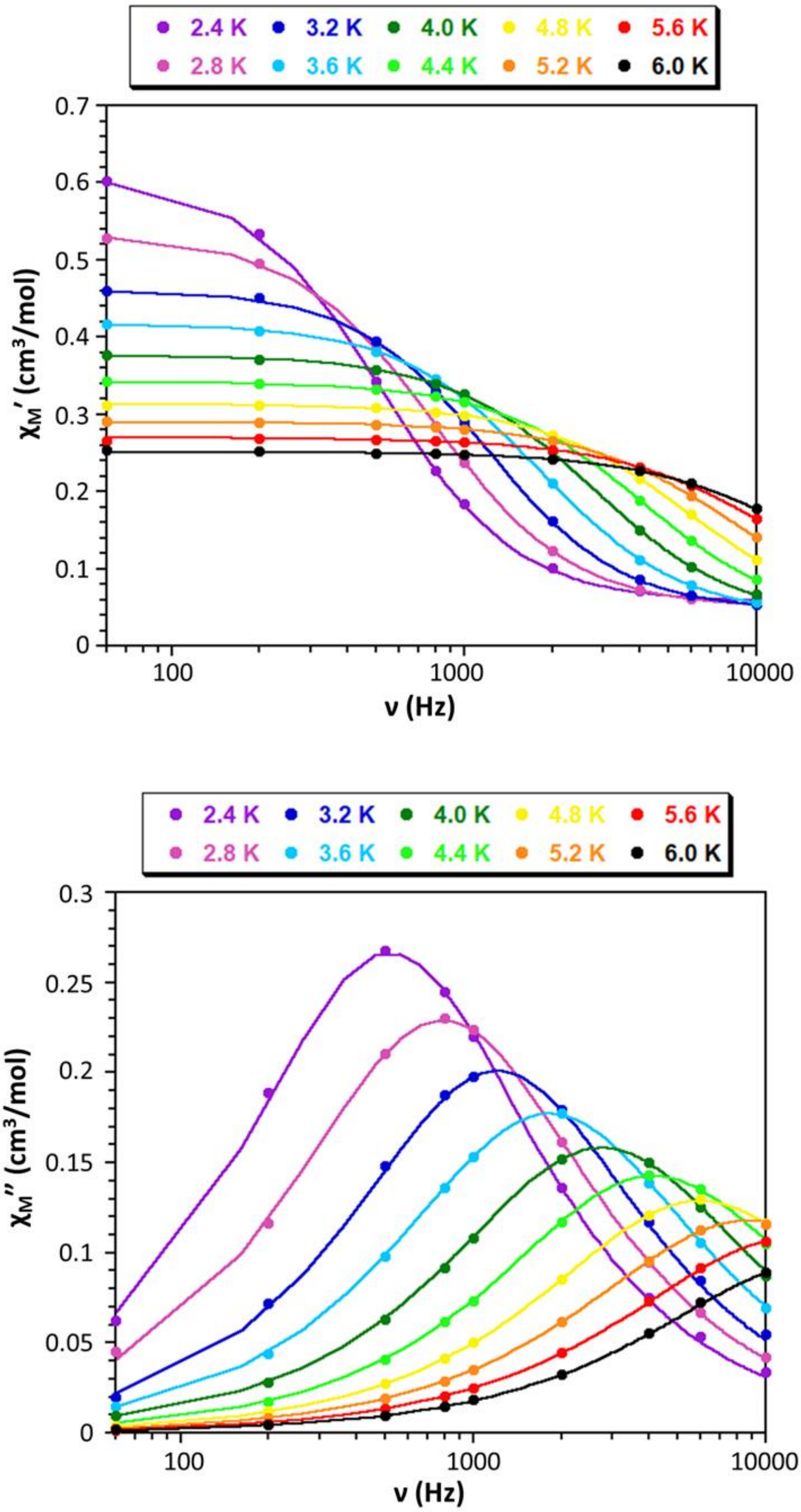

Figure S22. Variable temperature frequency dependence of the χ_M' (top) and χ_M'' (bottom) signals under 1000 Oe applied field for **1-Yb**. Solid lines represent the best fitting of experimental data to the Debye model.

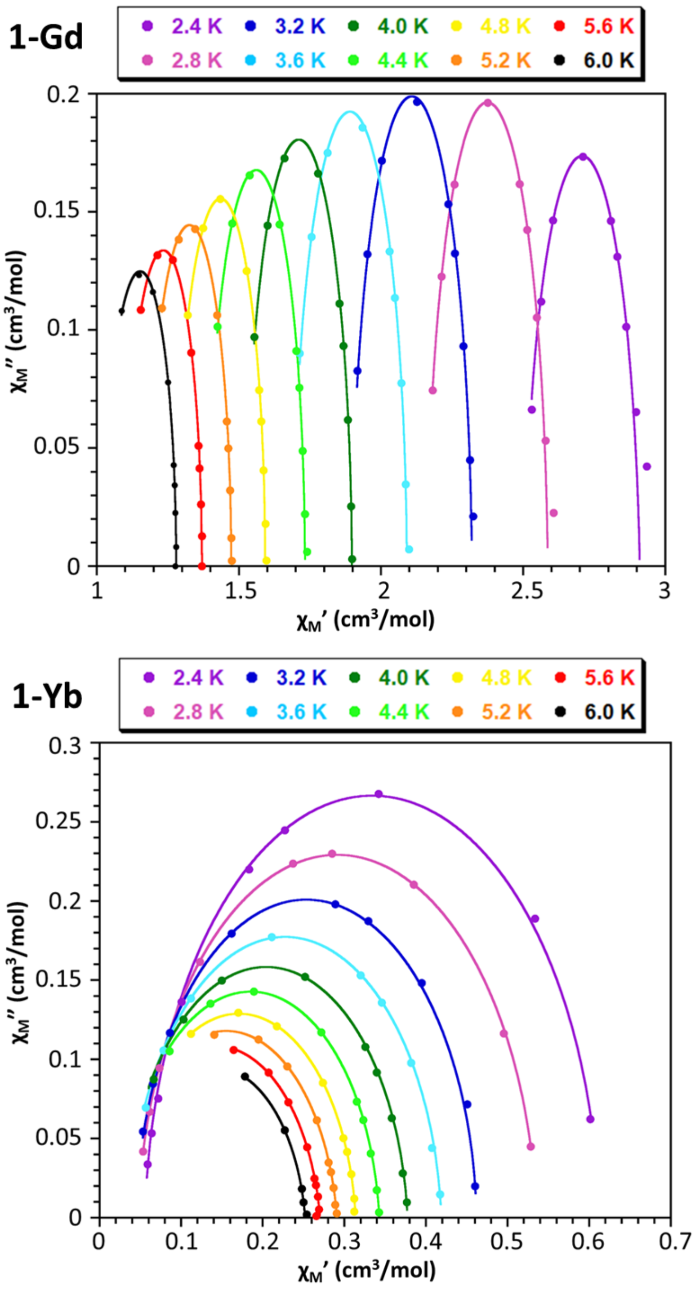

Figure S23. Cole-Cole plots for **1-Gd** (top) and **1-Yb** (bottom).

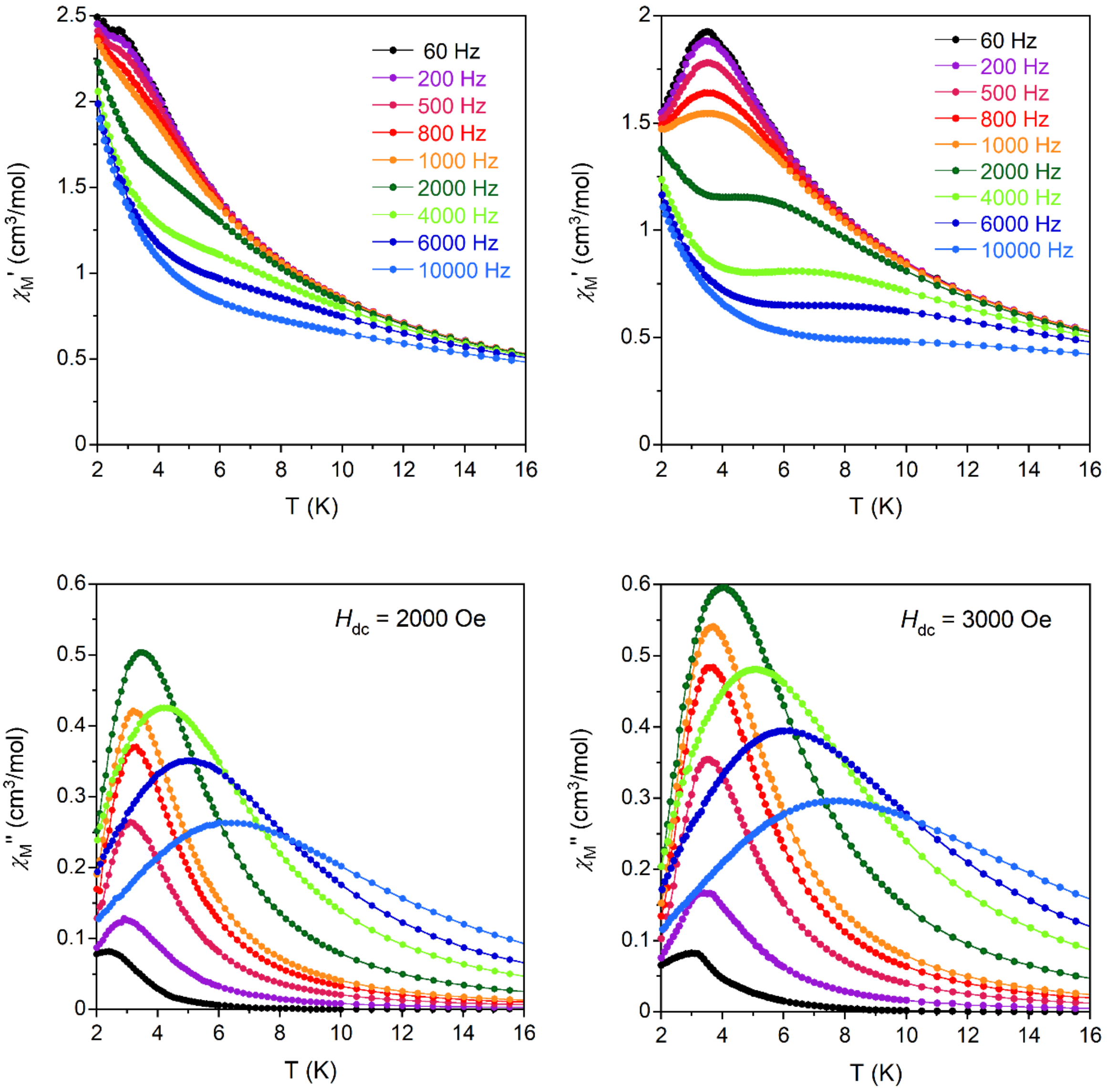

Figure S24. Temperature dependence of χ_M' (top) and χ_M'' (bottom) components of the *ac* susceptibility for **1-Gd** under external fields of 2000 Oe (left) and 3000 Oe (right).

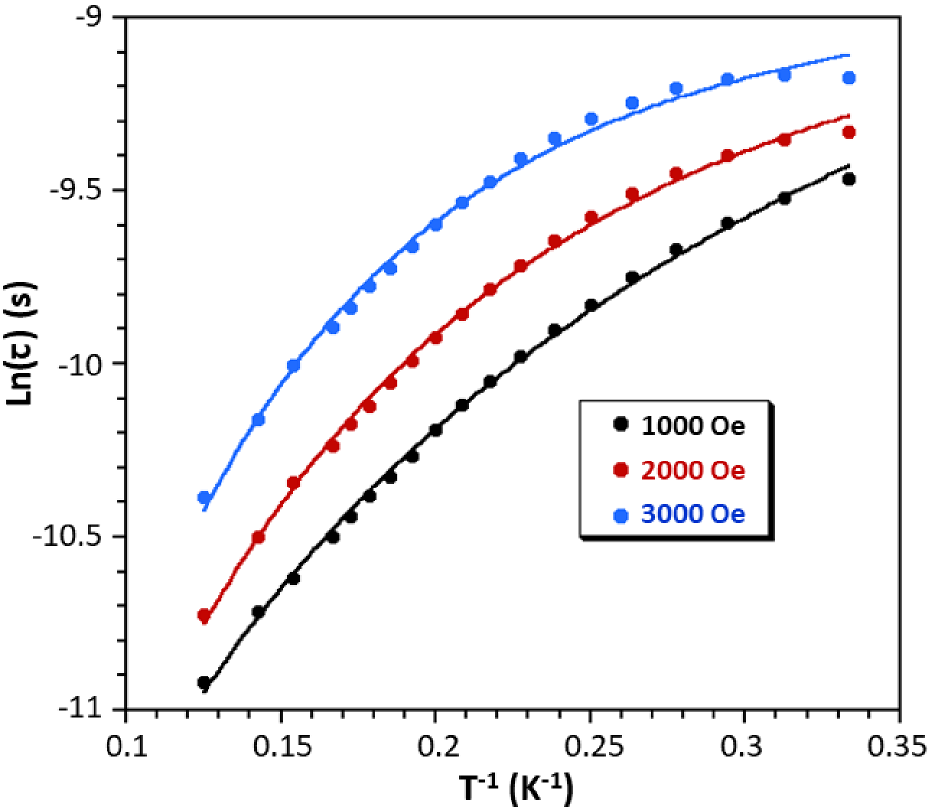

Figure S25. Relaxation times obtained under different applied fields for **1-Gd** with the best fitting of the data to eq. 4 (Eq. 4. $\tau^{-1} = CT^{n} + \tau_{QTM}^{-1}$).

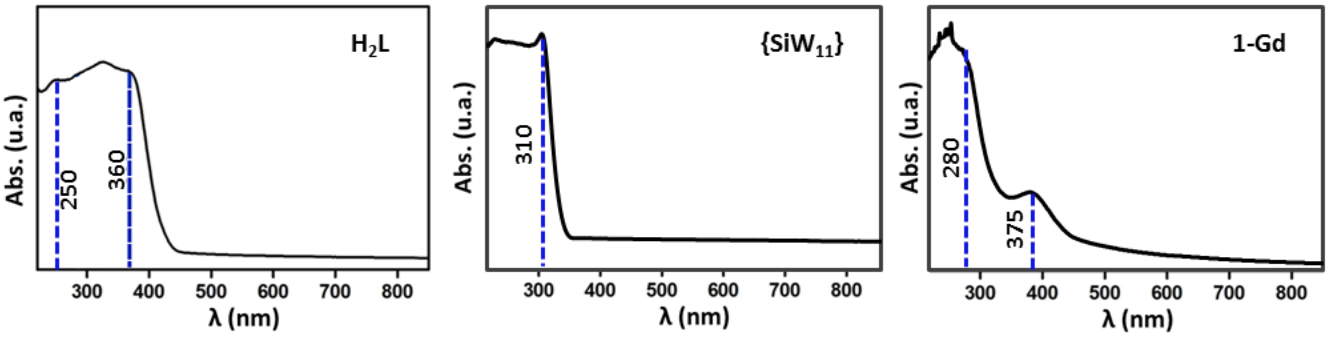

Figure S26. UV-Vis diffuse reflectance absorption spectra of H_2L ligand (left), $K_8[\alpha\text{-}SiW_{11}O_{39})]\cdot 13H_2O$ POM precursor (middle) and **1-Gd** (right).

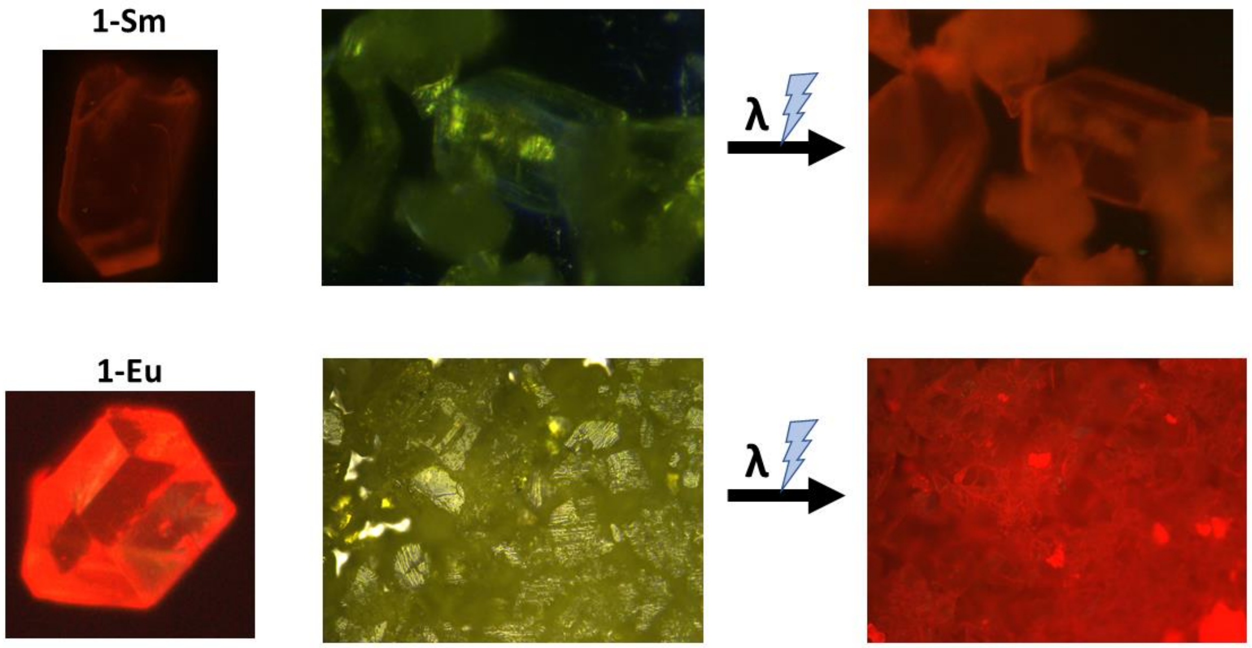

Figure S27. Left: Photographs of crystals of **1-Sm** (top) and **1-Eu** (bottom) under irradiation of UV light. Right: photographs of bulk crystalline samples before and after irradiation with UV light.

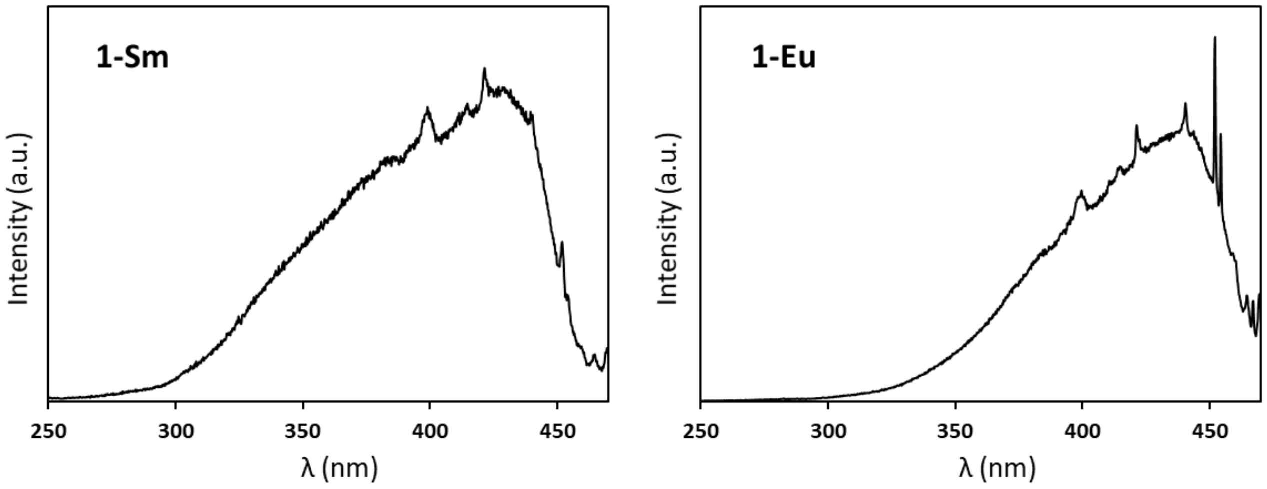

Figure S28. Low temperature (10K) solid state excitation spectra for **1-Sm** (left) and **1-Eu** (right) recorded for their most intense emission bands (600 and 614 nm, respectively).

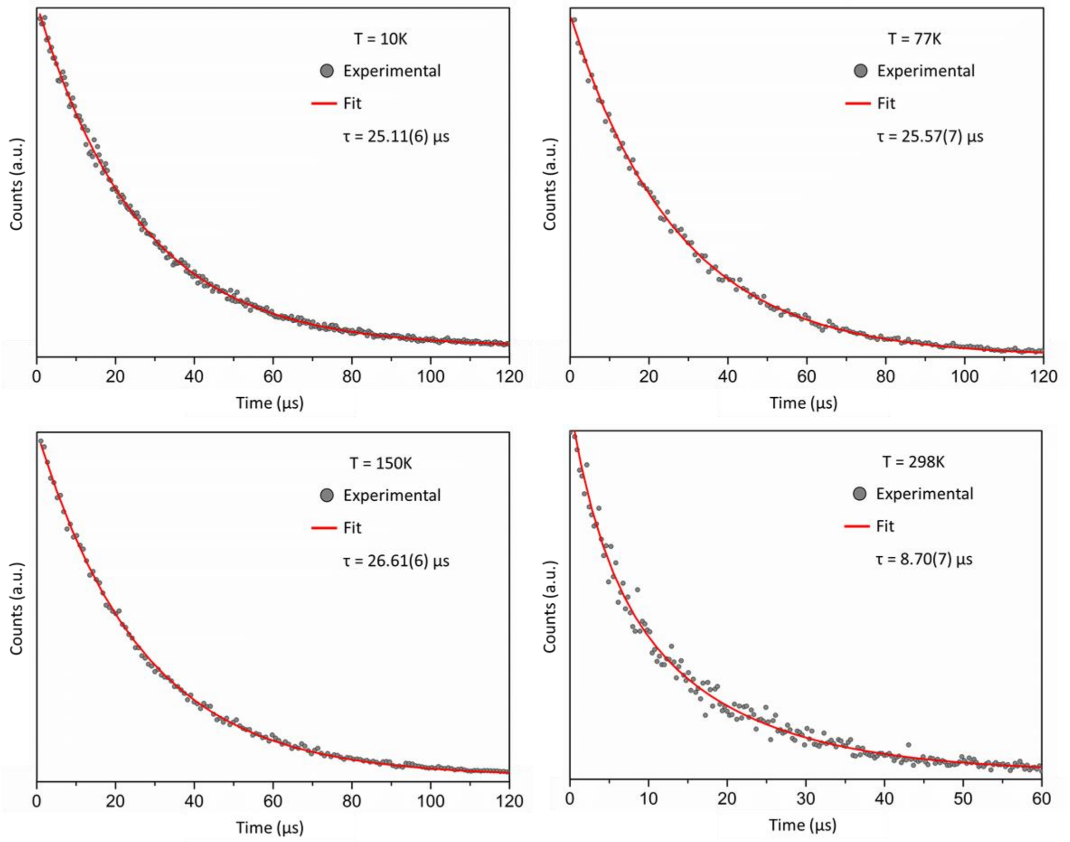

Figure S29. Luminescence decay curves for **1-Sm** at different temperatures upon excitation at 375 nm.

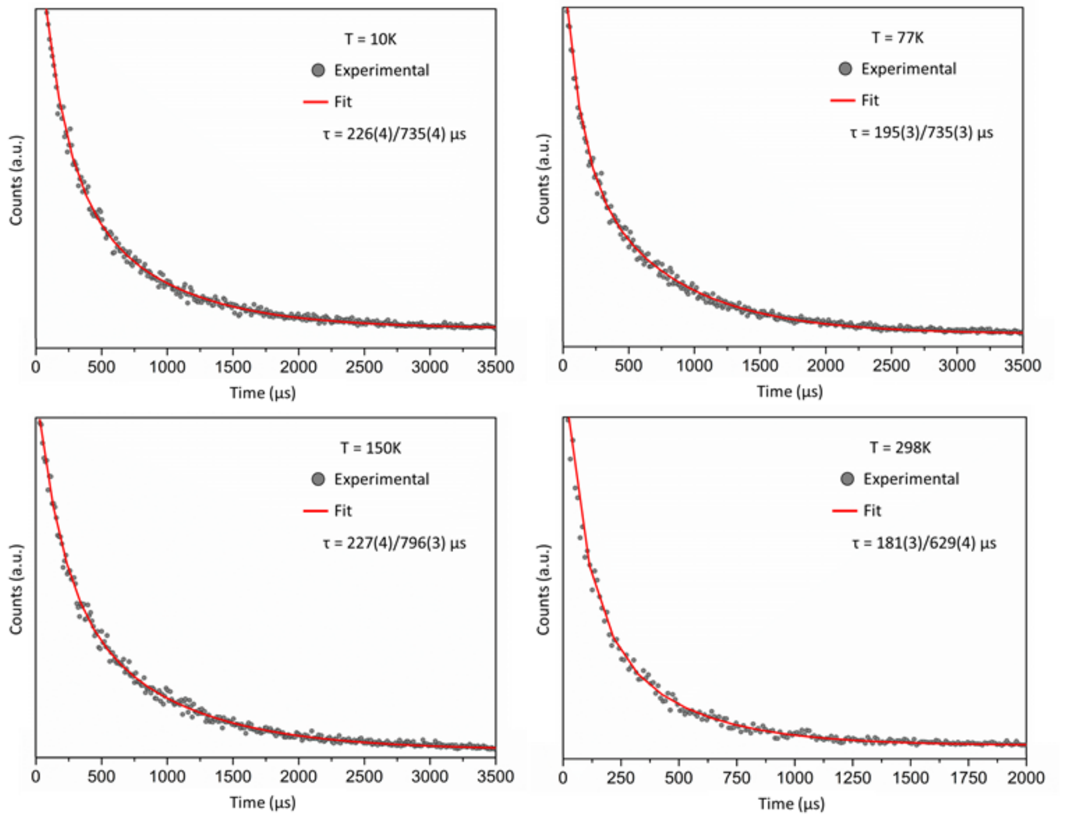

Figure S30. Luminescence decay curves for **1-Eu** at different temperatures upon excitation at 375 nm.

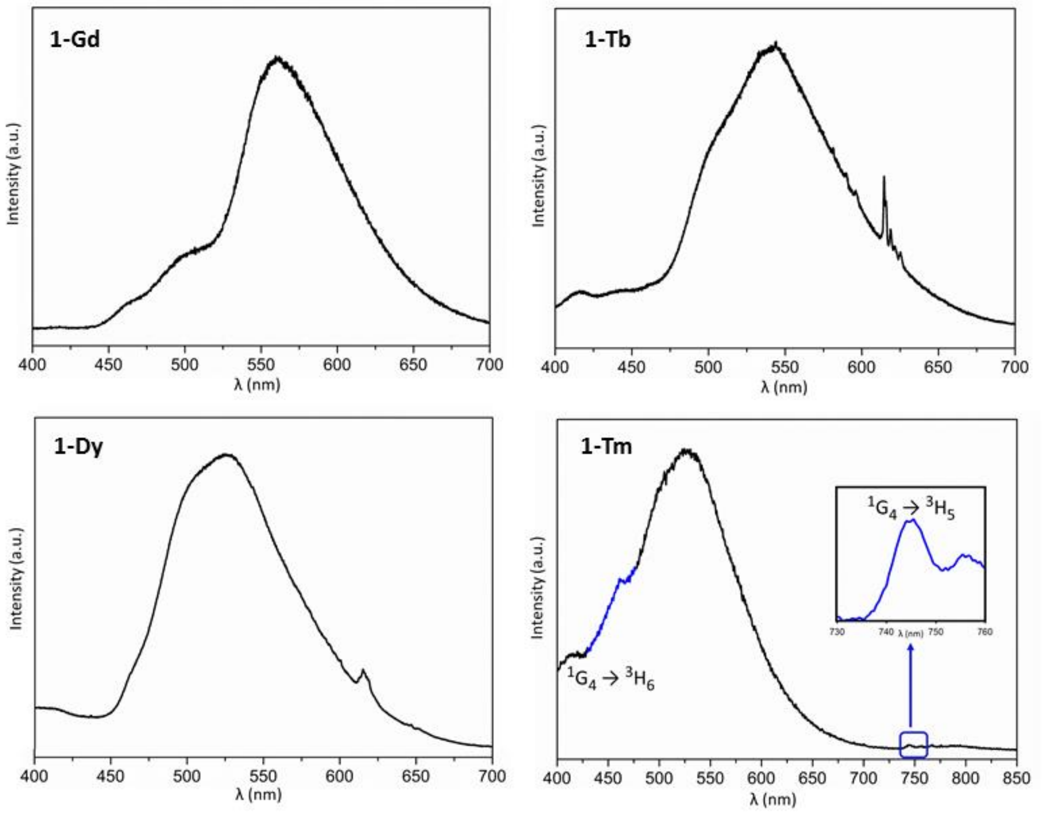

Figure S31. Solid state photoluminescence spectra of **1-Gd**, **1-Tb**, **1-Dy** and **1-Tm** recorded at 10K and upon excitation at 325 nm.

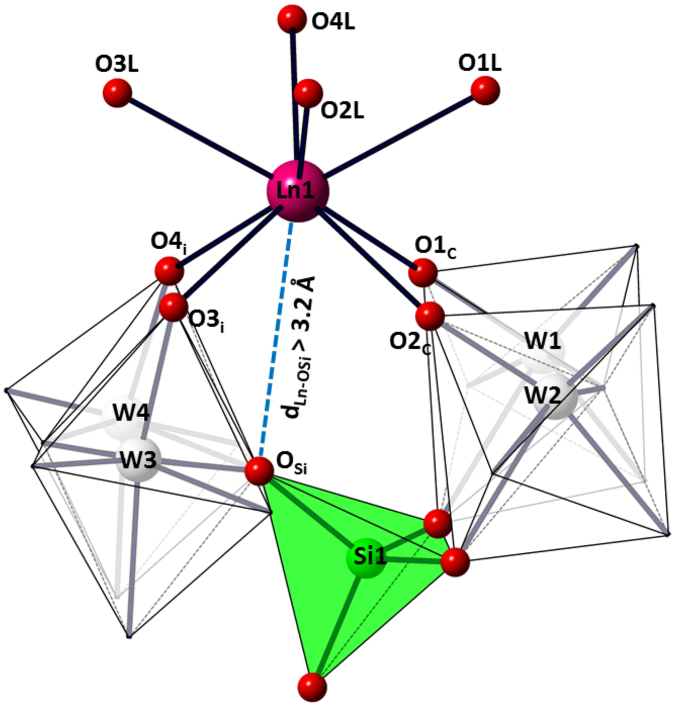

Figure S32. Scheme of coordination spheres of the emitting Ln centers in **1-Ln.** Labels O_c, O_i , and O_{Si} denote O atoms involved in corner sharing, those in intermediate situations between corner and edge sharing and central O atoms, respectively.

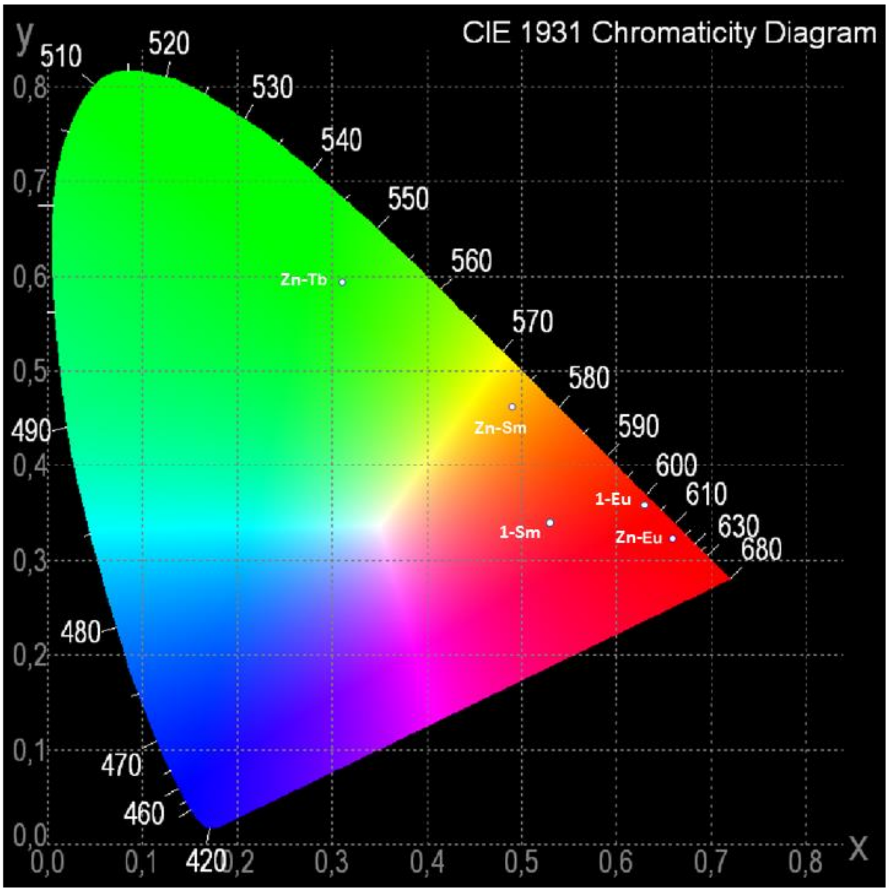

Figure S33. CIE 1931 x,y chromaticity coordinates as a function of the emission wavelengths for compounds **1-Ln** (Ln = Sm, Eu) compared to previously reported $[Zn(\mu\text{-}L)(\mu\text{-}OAc)Ln(NO_3)_2]\cdot MeCN$ (Ln = Sm, Eu, Tb) derivatives with the same H_2L ligand (Oyarzabal I. et al. *Dalton Trans.* **2016**, *45*, 9712).

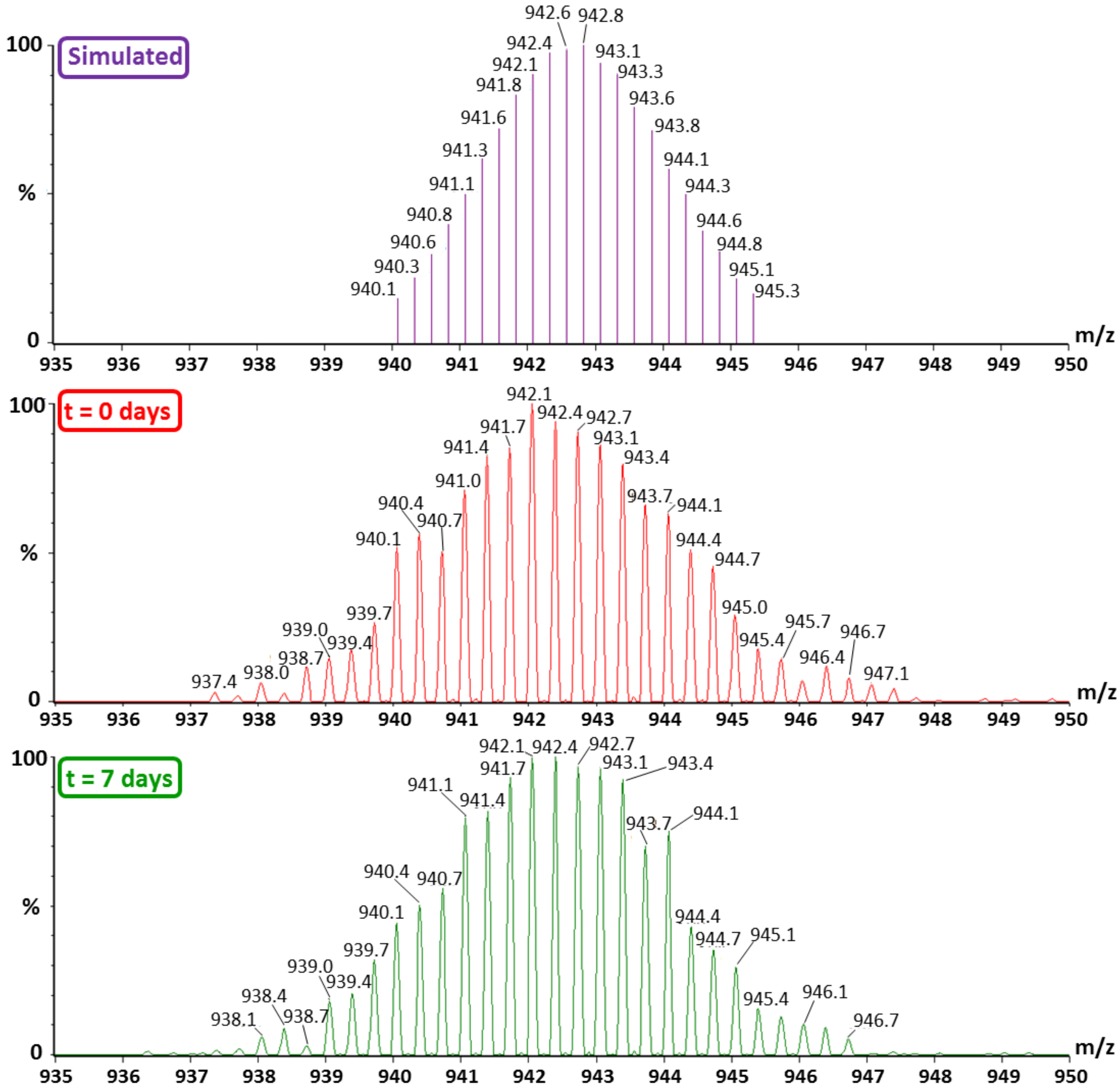

Figure S34. Expanded region of the negative ESI mass spectrum for the freshly prepared solution of **1-Tm** which corresponds to the **{Tm}$^{4-}$** series, compared to that recorded one week later and the simulated isotopic pattern for the $[Tm(H_2L)(SiW_{11}O_{39}) + K]^{4-}$ species.

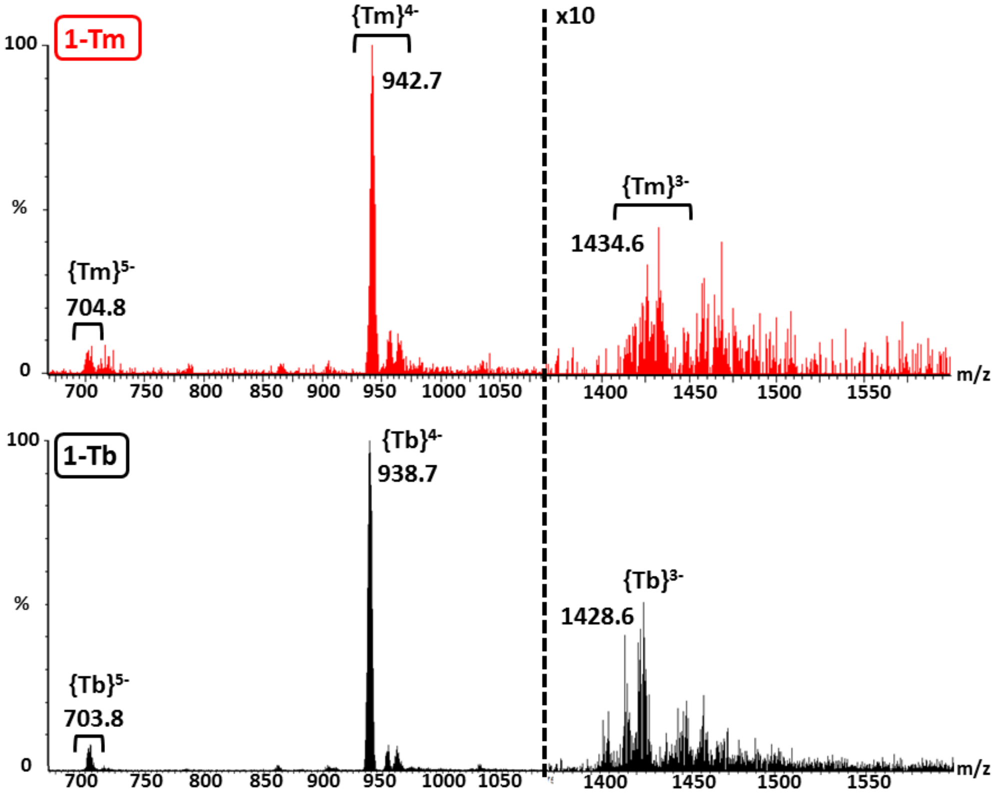

Figure S35. Negative ESI-MS spectra of **1-Tm** in H_2O/MeCN (1:1) mixture compared to that acquired for **1-Tb** derivative. The intensity of the m/z > 1300 region is increased (x10) for its better visualization.

Tables

Table S1. Thermal data for compounds **1-Ln**.

	**Dehydration (Δm, %)**		**Decomposition (°C)**	**Residue (m, %)**	
	**Δm_{calc} for $14H_2O$**	**Δm_{exp}**	**T_d**	**Calc. for $K_5LnO_{39}SiW_{11}$**	**Exp.**
**1-Sm**	6.7	5.9	665	20.2	20.3
**1-Eu**	6.7	6.7	740	20.2	20.7
**1-Gd**	6.7	7.0	690	20.2	21.0
**1-Tb**	6.6	6.7	750	20.2	20.7
**1-Dy**	6.6	6.9	705	20.2	21.1
**1-Ho**	6.6	5.9	670	20.2	20.1
**1-Er**	6.6	6.0	655	20.2	20.4
**1-Tm**	6.6	7.1	720	20.1	20.8
**1-Yb**	6.6	7.0	720	20.1	20.5
**1-Lu**	6.6	7.2	680	20.1	21.0

Table S2. Donor···acceptor distances (Å) for intramolecular N–H···O type hydrogen bonds in **1-Ln**.

Donor···Acceptor (Å)	**1-Sm**	**1-Eu**	**1-Gd**	**1-Tb**	**1-Dy**	**1-Ho**	**1-Er**	**1-Tm**	**1-Yb**	**1-Lu**
**N1A···O2LA**	2.67(3)	2.71(3)	2.69(2)	2.712(17)	2.65(3)	2.67(2)	2.76(3)	2.68(3)	2.69(2)	2.70(2)
**N2A···O3LA**	2.68(3)	2.68(3)	2.69(2)	2.676(18)	2.71(3)	2.70(2)	2.67(3)	2.70(3)	2.67(2)	2.70(2)
**N1B···O2LB**	2.70(4)	2.69(3)	2.69(2)	2.692(18)	2.71(3)	2.72(2)	2.67(3)	2.72(3)	2.71(2)	2.69(3)
**N2B···O3LB**	2.71(3)	2.73(3)	2.69(2)	2.684(19)	2.70(3)	2.67(2)	2.69(3)	2.69(3)	2.70(2)	2.68(2)
**N1C···O2LC**	2.66(3)	2.76(3)	2.70(2)	2.695(19)	2.71(3)	2.71(2)	2.67(3)	2.70(3)	2.73(2)	2.65(2)
**N2C···O3LC**	2.67(3)	2.69(3)	2.71(2)	2.670(17)	2.67(2)	2.65(2)	2.71(4)	2.70(3)	2.66(2)	2.70(2)

Table S3. Selected geometrical parameters for all the structures containing the H_2L ligand and lanthanide ions included in the CSD database: Intramolecular centroid···centroid (C_g···C_g) distances (Å) and dihedral angles (°) between aromatic rings.

1 Ligand			2 Ligands (Sandwich-type)			This work		
Refcode	Cg···Cg distance	Ring···ring angle	Refcode	Cg···Cg distance	Ring···ring angle		Cg···Cg distance	Ring···ring angle
Ln/Zn system			***Ln/M system (M = Cu, Zn)***					
BIPFIS	8.066	168.5	DONSAC	7.898	164.4		6.502	88.7
BIPFOY	8.050	168.8		7.837	151.3	**1-Sm**	6.471	97.3
BIPFUE	8.104	169.3	DONSEG	7.815	150.8		6.660	95.0
BIPGAL	8.080	169.6		7.893	164.2		6.578	88.5
BIPGEP	8.112	173.1	GALBUT	7.871	156.5	**1-Eu**	6.522	91.0
BIPGIT	8.192	164.8		7.895	161.6		6.621	91.7
BIQBIP	8.074	165.2	KAVXIR	7.856	157.5		6.442	87.6
	8.097	163.6		7.790	145.0	**1-Gd**	6.410	84.3
BIQBOV	8.062	165.1	KAVXOX	7.801	146.0		6.559	87.8
	8.090	164.2		7.838	147.1		6.363	85.5
BIQBUB	7.965	154.9	KAVXUD	7.833	153.7	**1-Tb**	6.492	87.3
	7.977	157.1		7.788	144.5		6.561	90.1
BIQCAI	8.017	161.3	KAVYAK	7.884	156.5		6.409	94.4
	7.945	155.8		7.908	161.6	**1-Dy**	6.435	88.2
IFUCER	8.045	163.7	KAWKOL	7.874	150.6		6.554	88.5
REJFIY	8.197	168.5		7.884	151.0		6.386	89.4
REJFOE	8.168	168.5	YUDVOK	7.878	151.9	**1-Ho**	6.444	90.5
REJGAR	8.126	166.5					6.572	84.8
REJGEV	8.113	167.9					6.388	87.7
REJGIZ	8.118	166.9				**1-Er**	6.339	85.4
REJGOF	8.094	165.9					6.514	87.5
REJGUL	8.086	166.4					6.496	89.0
REJHAS	8.062	165.4				**1-Tm**	6.460	90.4
REJHEW	8.054	165.5					6.406	87.8
Ln/Cu system							6.341	94.8
IMAGEL	8.096	164.3				**1-Yb**	6.397	91.1
IMAGIP	8.087	164.1					6.495	88.5
IMAGUB	8.072	164.0					6.391	86.5
IMAJAK	8.056	164.0				**1-Lu**	6.333	85.5
Ln/M system (M = Co, Ni)							6.478	88.3
IMAGOV	7.792	108.6						
IMAHAI	7.763	109.9						
IMAHEM	7.793	110.1						
IMAHOW	7.830	110.8						
IMAHUC	7.788	109.2						
IMAJEO	7.799	109.8						
IMAJIS	7.833	110.4						
IMAJUE	7.803	110.6						
IMAKAL	7.806	110.8						

Table S4. Continuous Shape Measurements (CSM) for the eight coordinated lanthanide atoms in compounds **1-Ln**.[a]

Atom	Shape	1-Sm	1-Eu	1-Gd	1-Tb	1-Dy	1-Ho	1-Er	1-Tm	1-Yb	1-Lu
Ln1A	SAPR-8	0.941	1.007	0.950	0.873	0.947	0.995	0.943	0.914	0.903	0.900
	TDD-8	1.624	1.466	1.610	1.648	1.723	1.764	1.674	1.437	1.796	1.663
	JBTPR-8	1.609	1.482	1.471	1.526	1.427	1.390	1.449	1.494	1.470	1.476
	BTPR-8	0.766	0.785	0.796	0.869	0.785	0.725	0.722	0.841	0.799	0.819
Ln1B	SAPR-8	1.059	1.015	1.066	0.974	0.943	0.901	0.997	0.867	0.907	0.896
	TDD-8	1.725	1.833	1.764	1.738	1.595	1.747	1.784	1.562	1.621	1.733
	JBTPR-8	1.394	1.286	1.384	1.349	1.508	1.500	1.395	1.404	1.529	1.379
	BTPR-8	0.785	0.696	0.710	0.671	0.812	0.865	0.685	0.781	0.828	0.763
Ln1C	SAPR-8	1.052	1.034	1.004	0.917	0.965	0.904	0.983	1.041	0.911	0.889
	TDD-8	1.602	1.419	1.583	1.488	1.516	1.543	1.529	1.853	1.633	1.511
	JBTPR-8	1.456	1.546	1.439	1.529	1.468	1.433	1.525	1.442	1.479	1.415
	BTPR-8	0.857	0.837	0.766	0.809	0.762	0.776	0.720	0.776	0.823	0.804

[a] *Abbreviations*. **SAPR:** square antiprism (D_{4d}); **TDD**: triangular dodecahedron (D_{2d}); **JBTPR:** Johnson biaugmented trigonal prism (C_{2v}) and; **BTPR:** biaugmented trigonal prism (C_{2v}).

Table S5. Lanthanide-Oxygen bond lengths (Å) and Ln···Ln distances (Å) in compounds **1-Ln.**

		1-Sm	1-Eu	1-Gd	1-Tb	1-Dy
**Ln1A-O1LA**	O_a	2.46(2)	2.50(2)	2.491(16)	2.476(13)	2.474(16)
**Ln1A-O4LA**		2.431(18)	2.405(15)	2.414(13)	2.406(10)	2.379(17)
**Ln1A-O2LA**	O_p	2.36(2)	2.36(2)	2.341(15)	2.325(13)	2.297(16)
**Ln1A-O3LA**		2.38(2)	2.372(18)	2.377(14)	2.355(12)	2.330(16)
**Ln1A-O1A**	O_{POM}	2.40(2)	2.35(2)	2.370(15)	2.325(11)	2.324(16)
**Ln1A-O2A**		2.389(19)	2.363(19)	2.367(16)	2.309(11)	2.336(17)
**Ln1A-O3A**		2.435(18)	2.412(16)	2.391(15)	2.376(11)	2.360(16)
**Ln1A-O4A**		2.353(17)	2.360(17)	2.352(15)	2.339(11)	2.351(16)
**Ln1B-O1LB**	O_a	2.451(19)	2.399(18)	2.402(15)	2.381(12)	2.447(18)
**Ln1B-O4LB**		2.556(18)	2.522(16)	2.492(13)	2.476(13)	2.378(16)
**Ln1B-O2LB**	O_p	2.372(16)	2.393(18)	2.352(14)	2.353(11)	2.286(18)
**Ln1B-O3LB**		2.339(18)	2.322(17)	2.313(14)	2.315(12)	2.331(16)
**Ln1B-O1B**	O_{POM}	2.363(18)	2.386(16)	2.345(12)	2.334(10)	2.330(17)
**Ln1B-O2B**		2.369(19)	2.367(17)	2.329(14)	2.321(11)	2.337(16)
**Ln1B-O3B**		2.387(17)	2.372(14)	2.368(13)	2.349(10)	2.382(16)
**Ln1B-O4B**		2.423(17)	2.399(17)	2.392(14)	2.395(10)	2.325(17)
**Ln1C-O1LC**	O_a	2.56(3)	2.51(2)	2.485(18)	2.457(13)	2.46(2)
**Ln1C-O4LC**		2.45(2)	2.42(2)	2.396(15)	2.399(12)	2.381(17)
**Ln1C-O2LC**	O_p	2.331(16)	2.329(15)	2.310(13)	2.303(10)	2.286(16)
**Ln1C-O3LC**		2.364(17)	2.348(17)	2.344(14)	2.345(10)	2.313(15)
**Ln1C-O1C**	O_{POM}	2.363(16)	2.364(15)	2.335(13)	2.333(10)	2.319(16)
**Ln1C-O2C**		2.34(2)	2.351(18)	2.334(14)	2.330(11)	2.323(16)
**Ln1C-O3C**		2.45(2)	2.43(2)	2.378(18)	2.396(11)	2.39(2)
**Ln1C-O4C**		2.379(18)	2.364(15)	2.353(17)	2.373(10)	2.336(17)
**O_a (average)**		2.485	2.459	2.447	2.433	2.420
**O_p (average)**		2.358	2.354	2.340	2.333	2.307
**O_{POM} (average)**		2.388	2.377	2.360	2.348	2.343
**LnA···LnB**		8.096(4)	8.040(3)	8.128(3)	8.180(2)	8.123(3)
**LnB···LnC**		8.213(12)	8.159(8)	8.244(9)	8.177(7)	7.993(7)
**LnC···LnA**		8.013(7)	8.024(5)	8.050(6)	8.001(5)	8.224(12)

Abbreviations: O_a: O atoms from the aldehyde group of the H_2L ligand; O_p: O atoms from the phenoxy group of the H_2L ligand; O_{POM}: O atoms delimiting the vacant site of the lacunary Keggin-type POM.

Table S5 (*continuation*). Lanthanide-Oxygen bond lengths (Å) and Ln···Ln distances (Å) in compounds **1-Ln.**

		**1-Ho**	**1-Er**	**1-Tm**	**1-Yb**	**1-Lu**
**Ln1A-O1LA**	O_a	2.466(13)	2.33(2)	2.34(2)	2.430(13)	2.320(13)
**Ln1A-O4LA**		2.358(15)	2.428(17)	2.46(2)	2.339(15)	2.383(18)
**Ln1A-O2LA**	O_p	2.277(13)	2.315(18)	2.264(18)	2.250(13)	2.288(16)
**Ln1A-O3LA**		2.319(14)	2.28(2)	2.257(18)	2.280(14)	2.225(18)
**Ln1A-O1A**	O_{POM}	2.327(12)	2.32(2)	2.28(2)	2.289(13)	2.274(17)
**Ln1A-O2A**		2.336(14)	2.33(2)	2.289(18)	2.311(14)	2.283(15)
**Ln1A-O3A**		2.375(13)	2.331(19)	2.295(19)	2.354(13)	2.288(15)
**Ln1A-O4A**		2.350(13)	2.377(17)	2.38(2)	2.308(14)	2.331(14)
**Ln1B-O1LB**	O_a	2.439(16)	2.36(2)	2.46(2)	2.401(16)	2.436(13)
**Ln1B-O4LB**		2.364(13)	2.46(2)	2.329(18)	2.328(13)	2.308(15)
**Ln1B-O2LB**	O_p	2.277(15)	2.310(16)	2.26(2)	2.253(15)	2.258(17)
**Ln1B-O3LB**		2.325(13)	2.30(2)	2.291(19)	2.283(14)	2.290(14)
**Ln1B-O1B**	O_{POM}	2.304(13)	2.325(18)	2.31(2)	2.290(15)	2.300(16)
**Ln1B-O2B**		2.290(13)	2.35(2)	2.28(2)	2.285(14)	2.309(15)
**Ln1B-O3B**		2.389(13)	2.332(18)	2.360(19)	2.345(13)	2.322(14)
**Ln1B-O4B**		2.315(14)	2.344(19)	2.30(2)	2.307(13)	2.315(14)
**Ln1C-O1LC**	O_a	2.455(16)	2.365(19)	2.34(2)	2.433(16)	2.335(16)
**Ln1C-O4LC**		2.364(14)	2.41(3)	2.466(18)	2.341(15)	2.426(18)
**Ln1C-O2LC**	O_p	2.252(13)	2.276(17)	2.28(2)	2.225(13)	2.277(13)
**Ln1C-O3LC**		2.312(13)	2.25(2)	2.26(2)	2.273(13)	2.211(13)
**Ln1C-O1C**	O_{POM}	2.305(13)	2.320(19)	2.307(19)	2.281(13)	2.286(16)
**Ln1C-O2C**		2.325(14)	2.313(18)	2.31(2)	2.280(14)	2.282(13)
**Ln1C-O3C**		2.379(15)	2.32(2)	2.358(19)	2.372(15)	2.290(15)
**Ln1C-O4C**		2.338(13)	2.38(2)	2.387(19)	2.322(13)	2.328(15)
**O_a (average)**		2.408	2.392	2.399	2.379	2.368
**O_p (average)**		2.294	2.289	2.269	2.261	2.258
**O_{POM} (average)**		2.336	2.337	2.321	2.312	2.301
**LnA···LnB**		8.051(3)	8.108(4)	8.054(10)	8.059(3)	8.051(4)
**LnB···LnC**		7.981(6)	8.215(13)	8.044(4)	7.990(7)	8.204(13)
**LnC···LnA**		8.216(2)	8.018(9)	8.165(13)	8.226(12)	8.004(10)

Abbreviations: O_a: O atoms from the aldehyde group of the H_2L ligand; O_p: O atoms from the phenoxy group of the H_2L ligand; O_{POM}: O atoms delimiting the vacant site of the lacunary Keggin-type POM.

Table S6. Geometrical parameters (Å, °) of intermolecular π–π interactions in **1-Ln**.

**Centroids**	**Cg···Cg**	**ANG**	**Slippage**	**Centroids**	**Cg···Cg**	**ANG**	**Slippage**
**1-Sm**				**1-Ho**			
Cg1···Cg3^{i}	3.52(2)	6	0.860	Cg1···Cg6^{xii}	3.564(11)	5	1.148
Cg2···Cg5^{ii}	3.531(18)	4	0.549	Cg2···Cg3^{xiii}	3.535(13)	5	0.547
Cg3···Cg1^{iii}	3.52(2)	6	0.512	Cg3···Cg2^{xiii}	3.536(13)	5	0.821
Cg4···Cg6^{iv}	3.538(15)	5	1.153	Cg4···Cg5^{xiv}	3.540(12)	2	0.605
Cg5···Cg2^{ii}	3.529(18)	4	0.762	Cg5···Cg4^{xiv}	3.540(12)	2	0.750
Cg6···Cg4^{v}	3.538(15)	5	0.907	Cg6···Cg1^{xii}	3.563(11)	5	0.888
**1-Eu**				**1-Er**			
Cg1···Cg3^{i}	3.550(17)	3	0.788	Cg1···Cg6^{xii}	3.532(18)	4	0.582
Cg2···Cg5^{vi}	3.539(16)	3	0.592	Cg2···Cg3^{vii}	3.524(19)	7	1.006
Cg3···Cg1^{iii}	3.551(17)	3	0.600	Cg3···Cg2^{vii}	3.526(19)	7	0.576
Cg4···Cg6^{iv}	3.540(14)	4	1.034	Cg4···Cg5^{xiv}	3.552(17)	6	1.168
Cg5···Cg2^{vi}	3.538(16)	3	0.774	Cg5···Cg4^{xiv}	3.552(17)	6	0.852
Cg6···Cg4^{v}	3.540(14)	4	0.813	Cg6···Cg1^{xii}	3.532(18)	4	0.809
**1-Gd**				**1-Tm**			
Cg1···Cg3^{i}	3.576(15)	7	0.921	Cg1···Cg6^{ix}	3.539(17)	5	0.853
Cg2···Cg5^{vii}	3.550(14)	4	0.578	Cg2···Cg4^{xv}	3.527(18)	5	0.855
Cg3···Cg1^{iii}	3.576(15)	7	0.494	Cg3···Cg5^{i}	3.56(2)	3	0.803
Cg4···Cg6^{iv}	3.591(11)	6	1.198	Cg4···Cg2^{xvi}	3.528(18)	5	0.601
Cg5···Cg2^{vii}	3.550(14)	4	0.776	Cg5···Cg3^{iii}	3.56(2)	3	0.612
Cg6···Cg4^{v}	3.593(11)	6	0.852	Cg6···Cg1^{ix}	3.541(17)	5	1.097
**1-Tb**				**1-Yb**			
Cg1···Cg3^{i}	3.568(11)	6	1.012	Cg1···Cg6^{xii}	3.589(12)	7	1.210
Cg2···Cg5^{viii}	3.618(11)	6.	0.473	Cg2···Cg3^{xiii}	3.551(13)	6	0.518
Cg3···Cg1^{iii}	3.568(11)	6	0.672	Cg3···Cg2^{xiii}	3.551(13)	6	0.869
Cg4···Cg6^{iv}	3.589(9)	5	1.088	Cg4···Cg5^{xiv}	3.541(13)	4	0.598
Cg5···Cg2^{viii}	3.618(11)	6	0.779	Cg5···Cg4^{xiv}	3.540(13)	4	0.834
Cg6···Cg4^{v}	3.589(9)	5	0.784	Cg6···Cg1^{xii}	3.588(12)	7	0.874
**1-Dy**				**1-Lu**			
Cg1···Cg6^{ix}	3.575(13)	6	1.189	Cg1···Cg6^{xvii}	3.532(14)	4	0.587
Cg2···Cg3^{x}	3.577(17)	6	0.589	Cg2···Cg4^{iii}	3.564(15)	5	0.907
Cg3···Cg2^{x}	3.577(17)	6	0.976	Cg3···Cg5^{v}	3.570(13)	6	1.191
Cg4···Cg5^{xi}	3.542(16)	4	0.529	Cg4···Cg2^{i}	3.563(15)	5	0.607
Cg5···Cg4^{xi}	3.540(16)	4	0.773	Cg5···Cg3^{iv}	3.569(13)	6	0.874
Cg6···Cg1^{ix}	3.576(13)	6	0.854	Cg6···Cg1^{xvii}	3.533(14)	4	0.790

Cgi = Centroid of the aromatic ring defined by the following atoms:
i = 1: C2A, C3A, C4A, C5A, C6A, C7A; i = 2: C12A, C13A, C14A, C15A, C16A, C17A; i = 3: C2B, C3B, C4B, C5B, C6B, C7B; i = 4: C12B, C13B, C14B, C15B, C16B, C17B; i = 5: C2C, C3C, C4C, C5C, C6C, C7C; i = 6: C12C, C13C, C14C, C15C, C16C, C17C.
Cg···Cg = distance between centroids; ANG = dihedral angle between planes containing both rings; Slippage = distance between one centroid and its perpendicular projection to the plane containing the second ring.
Symmetry codes: (i) x, -1+y, z; (ii) 3-x, 1-y, 1-z; (iii) x, 1+y, z; (iv) -1+x, y, z; (v) 1+x, y, z; (vi) 1-x, -1-y, -z; (vii) 2-x, 1-y, 1-z; (viii) 1-x, -y, -z; (ix) 1-x, 2-y, 1-z; (x) -x, 1-y, 1-z; (xi) -x, 1-y, -z; (xii) 2-x, 1-y, 2-z; (xiii) 1-x, -y, 2-z; (xiv) 1-x, -y, 1-z; (xv) x, 1+y, 1+z; (xvi) x, -1+y, -1+z; (xvii) 2-x, 2-y, 2-z.

Table S7. Br···Br distances (Å) in compounds **1-Ln.**

	**1-Sm[a]**	**1-Eu[b]**	**1-Gd[c]**	**1-Tb[d]**	**1-Dy[e]**	**1-Ho[f]**	**1-Er[g]**	**1-Tm[h]**	**1-Yb[j]**	**1-Lu[k]**
BrA···BrB	3.706(5)	3.817(5)	3.705(5)	3.759(4)	3.677(4)	3.653(4)	3.657(5)	3.907(5)	3.645(4)	3.656(4)
BrA···BrC	3.962(6)	4.016(5)	3.958(5)	3.722(4)	3.953(4)	3.903(3)	3.882(5)	3.826(7)	3.890(4)	3.924(4)
BrB···BrC	3.960(6)	3.914(5)	3.989(4)	4.086(3)	3.923(5)	3.967(3)	3.923(5)	3.741(6)	3.945(3)	3.866(4)

Symmetry codes: [a] Br2A···Br1B^{i}: 2-x, -y, 1-z; Br2A···Br2C^{ii}: -1+x, -1+y, z; Br1B···Br2C^{iii}: 3-x, 2-y, 1-z; [b] Br2A···Br1B^{iv}: -x, -1-y, -z; Br2A···Br2C^{ii}; Br1B···Br2C^{v}: 1-x, -y, -z; [c] Br2A···Br1B^{vi}: 1-x, 1-y, 1-z; Br2A···Br2C^{ii}; Br1B···Br2C^{vii}: 2-x, 2-y, 1-z; [d] Br2A···Br1B^{viii}: -x, -y, -z; Br2A···Br2C^{ii}; Br1B···Br2C^{ix}: 1-x, 1-y, -z; [e] Br2A···Br2B^{x}: 1+x, 1+y, 1+z; Br2A···Br2C^{xi}: x, y, 1+z; Br2B···Br2C^{ii}; [f] Br2A···Br2B^{x}; Br2A···Br2C^{xi}; Br2B···Br2C^{ii}; [g] Br1A···Br1B^{x}; Br1A···Br1C^{xii}: 1+x, 1+y, z; Br1B···Br1C^{xiii}: x, y, -1+z; [h] Br1A···Br2B^{vi}; Br1A···Br1C^{xi}; Br2B···Br1C^{ix}; [j] Br2A···Br2B^{x}; Br2A···Br2C^{xi}; Br2B···Br2C^{ii}; [k] Br1A···Br2B^{xiv}: 3-x, 2-y, 2-z; Br1A···Br1C^{xii}; Br2B···Br1C^{xv}: 2-x, 1-y, 2-z.

Table S8. Values of *C*, *n* and τ_{QTM}^{-1} parameters obtained from the best fitting of relaxation times for different applied fields in **1-Gd.**

	C	***n***	$\boldsymbol{\tau_{QTM}^{-1}}$ **(s^{-1})**
$\boldsymbol{\tau^{-1} = CT^{n}}$ $(\mathrm{Eq}.3)$			
1000 Oe	2217	1.54	
$\boldsymbol{\tau^{-1} = CT^{n} + \tau_{QTM}^{-1}}$ $(Eq.4)$			
1000 Oe	1470	1.73	2540
2000 Oe	348	2.28	6474
3000 Oe	98	2.69	7144

Table S9. Ln–O_{POM}–W bond angles in **1-Ln**. Note the difference between oxygen atoms involved in corner sharing (O_c) and those in intermediate situations between corner and edge sharing (O_i).

	1-Sm	1-Eu	1-Gd	1-Tb	1-Dy
**Ln—O1A$_C$—W1A**	155.1(10)	155.6(19)	153.6(7)	156.4(6)	155.7(10)
**Ln—O2A$_C$—W2A**	156.1(11)	157.2(11)	156.0(8)	157.3(6)	157.4(10)
**Ln—O3A$_i$—W3A**	128.3(11)	130.3(10)	130.9(9)	131.4(6)	132.7(9)
**Ln—O4A$_i$—W4A**	133.1(7)	134.3(8)	133.8(7)	133.9(5)	134.2(9)
**Ln—O1B$_C$—W1B**	156.5(11)	156.2(9)	156.7(7)	156.8(6)	155.7(9)
**Ln—O2B$_C$—W2B**	156.1(11)	155.0(10)	155.4(8)	154.6(6)	157.6(9)
**Ln—O3B$_i$—W3B**	133.7(10)	132.2(8)	132.0(7)	133.0(5)	130.5(9)
**Ln—O4B$_i$—W4B**	131.5(9)	130.3(8)	131.2(7)	129.7(5)	135.7(7)
**Ln—O1C$_C$—W1C**	155.2(9)	154.6(9)	156.0(8)	155.3(5)	155.0(9)
**Ln—O2C$_C$—W2C**	158.0(11)	155.6(9)	155.9(9)	156.9(6)	157.1(9)
**Ln—O3C$_i$—W3C**	131.4(8)	130.7(8)	130.4(8)	131.5(5)	131.5(8)
**Ln—O4C$_i$—W4C**	133.9(10)	134.7(10)	133.8(9)	133.8(5)	134.5(9)
	**1-Ho**	**1-Er**	**1-Tm**	**1-Yb**	**1-Lu**
**Ln—O1A$_C$—W1A**	156.5(7)	156.6(13)	158.5(12)	156.7(7)	157.9(9)
**Ln—O2A$_C$—W2A**	156.7(8)	154.0(10)	157.8(11)	156.8(8)	157.0(8)
**Ln—O3A$_i$—W3A**	132.0(7)	133.7(9)	136.1(10)	132.9(7)	136.5(7)
**Ln—O4A$_i$—W4A**	134.0(7)	132.2(9)	132.3(9)	135.6(8)	134.1(9)
**Ln—O1B$_C$—W1B**	156.3(7)	157.9(11)	153.7(11)	157.9(8)	154.4(9)
**Ln—O2B$_C$—W2B**	157.0(8)	154.1(13)	157.2(12)	158.0(8)	156.8(8)
**Ln—O3B$_i$—W3B**	131.8(8)	132.7(9)	132.8(11)	132.3(8)	133.2(8)
**Ln—O4B$_i$—W4B**	135.5(7)	132.0(10)	135.2(10)	136.2(7)	135.6(8)
**Ln—O1C$_C$—W1C**	155.1(7)	158.5(13)	158.0(11)	156.9(8)	156.7(8)
**Ln—O2C$_C$—W2C**	155.7(8)	154.5(9)	156.4(12)	156.5(7)	154.7(9)
**Ln—O3C$_i$—W3C**	130.5(7)	134.3(10)	135.2(11)	131.8(7)	135.6(8)
**Ln—O4C$_i$—W4C**	135.3(7)	131.4(11)	131.5(10)	135.2(7)	133.0(7)
